# Supplementary material for: Patterns of Reliability: Assessing the Reproducibility and Integrity of DNA Methylation Measurement
Source: Patterns (N Y). 2020 Apr 23;1(2):100014. doi: 10.1016/j.patter.2020.100014 (PMC7467214; doi:10.1016/j.patter.2020.100014)
Supplement: Document S1. Supplemental Experimental Procedures, Figures S1–S10, and Tables S1–S4 [file mmc1.pdf]

PATTER, Volume *1*

Supplemental Information

Patterns of Reliability:

Assessing the Reproducibility and Integrity

of DNA Methylation Measurement

Karen Sugden, Eilis J. Hannon, Louise Arseneault, Daniel W. Belsky, David L. Corcoran, Helen L. Fisher, Renate M. Houts, Radhika Kandaswamy, Terrie E. Moffitt, Richie Poulton, Joseph A. Prinz, Line J.H. Rasmussen, Benjamin S. Williams, Chloe C.Y. Wong, Jonathan Mill, and Avshalom Caspi

SUPPLEMENTAL EXPERIMENTAL PROCEDURES

Section S1: Describing the landscape of CpG probe reliability

This section relates to Main Text Results section entitled
'Reliability of CpG probes is low and highly variable'.

1.1 Reliability of CpG probes is low and highly variable. We began by assessing the distribution of probe-probe Intraclass Correlations (ICCs, henceforth 'reliability') across the 438,593 probes present on both the 450K and EPIC BeadChips in our data. Probe ICCs ranged from -0.28 to 1.00 (**Data S1,** https://osf.io/83ucs/). As shown in **Figure S1**, probe reliabilities were skewed towards zero, with a mean of 0.21 (median = 0.09). This is low reliability considering that, in the context of establishing reliable measurement, ICCs below .4 are considered "poor," those between .4 to .6 are considered "fair", between .6 to .75 "good", and above .75 "excellent".[1]

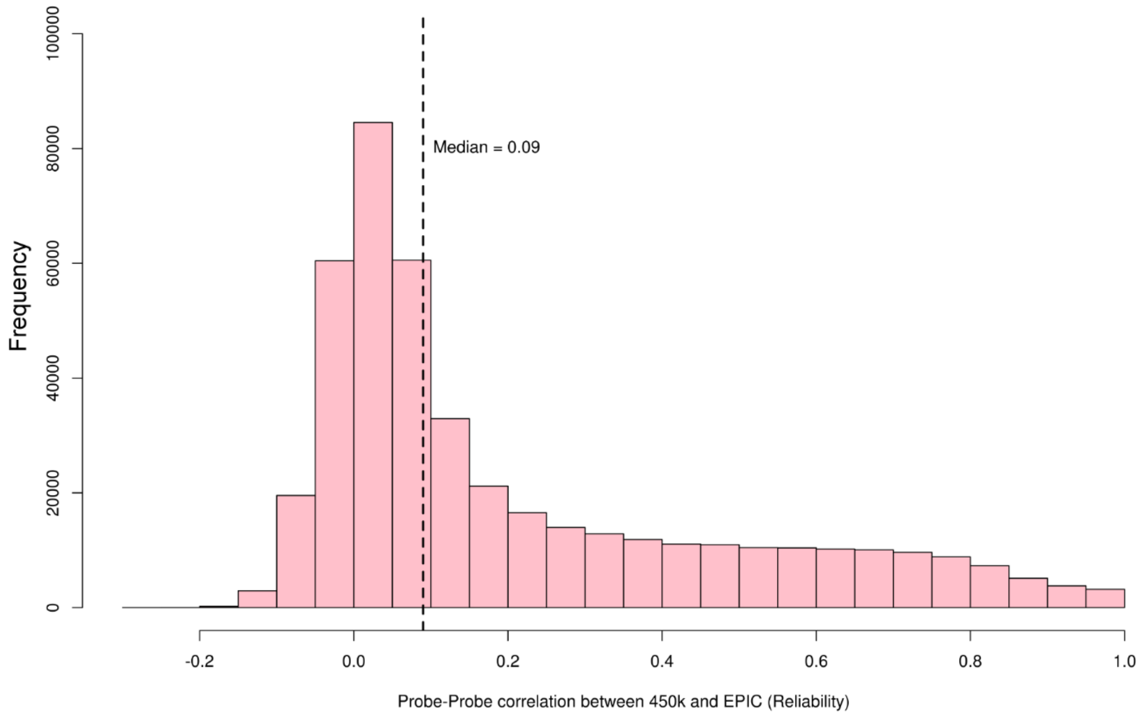

Figure S1: Distribution of reliability correlations for probes common to the 450K and EPIC BeadChips.

Low reliability might arise through experimental factors not related solely to poor probe performance. We therefore tested whether the pattern of reliabilities we observed might be due to such stochastic processes by comparing our reliabilities against those reported by Logue *et al.*[2], who also compared reliabilities of probes across 450K and EPIC BeadChips. The reliabilities were highly correlated (r = 0.86, p < 0.01, **Figure S2**), suggesting the reliabilities are reproducible and systematic in pattern.

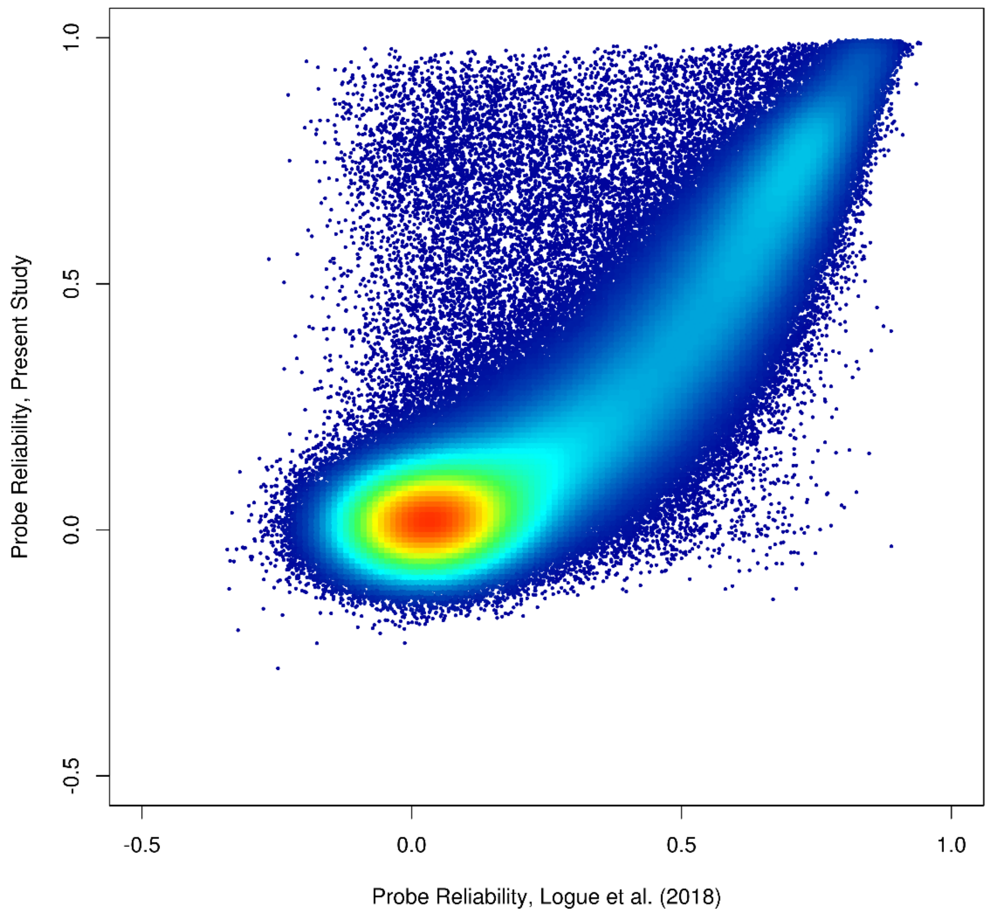

Figure S2. Differential probe reliabilities were consistent across studies. The y-axis plots probe reliabilities (as ICCs) in the present study, and the x-axis plots the reliabilities (as ICCs) reported by Logue *et al.*. Reliabilities were highly correlated (r = 0.86). Reliabilities were derived from comparisons between 450K and EPIC BeadChip.

An additional source of low reliability could be due to between-array (i.e. 450K vs EPIC) differences in probe performance. While this is unlikely since previous studies have documented low reliabilities in 450K-450K probe comparisons[3,4] and EPIC-EPIC probe comparisons[2], we nonetheless sought to independently determine whether within-array reliability followed similar patterns to between-array reliability. For this, we created a new reliability dataset comprised EPIC-EPIC (i.e. within-array) comparisons for a subset of Dunedin (N = 28) study samples (for comparison purposes, we restricted analysis to the ~440,000 probes overlapping with the 450K array as described throughout this manuscript). We sought to test if the distribution of reliabilities was similar between these two datasets.

We found that, like the between-array comparison, reliabilities for the within-array comparison were low and skewed towards zero (median = 0.26), and the two sets of reliabilities were significantly correlated with one another (*r* = 0.77, **Figure S3**). This suggests that differences between 450K and EPIC BeadChips are unlikely to be the sole cause of low probe reliability.

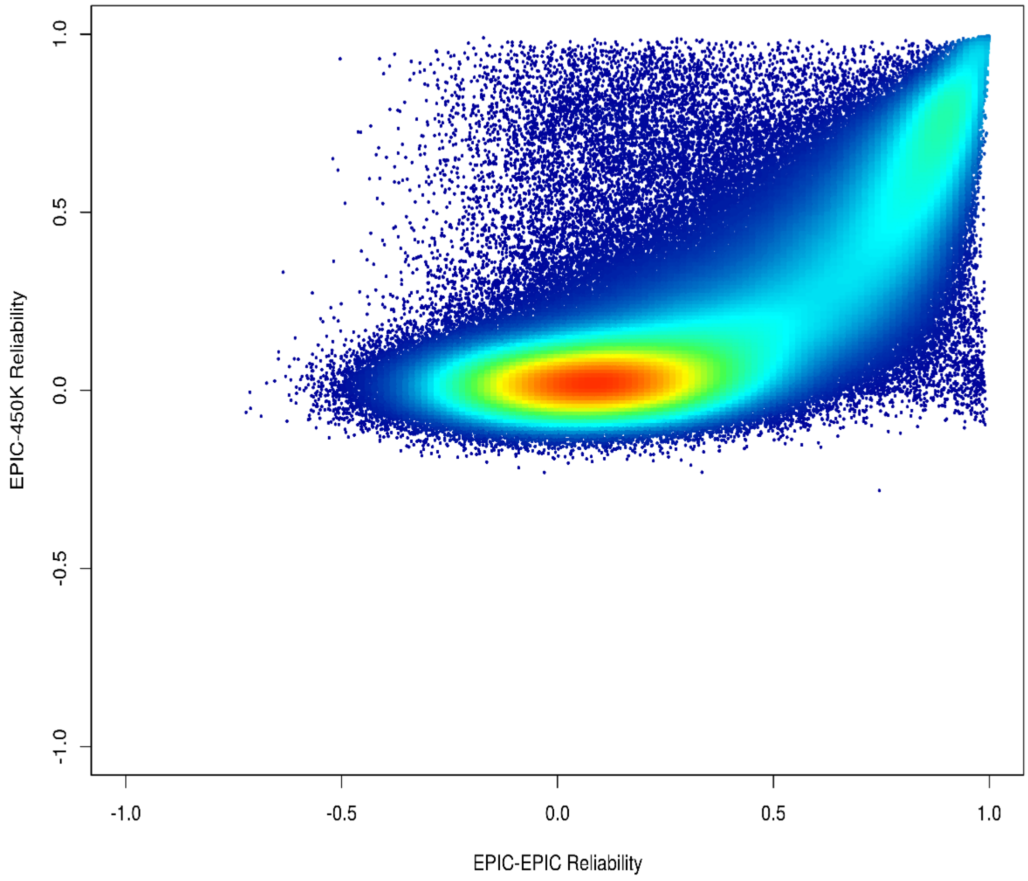

Figure S3. Between-array and within-array reliabilities are correlated. The y-axis plots the 450K-EPIC probe reliabilities used in the present study, and the x-axis plots EPIC-EPIC probe reliabilities from a subset of 28 individuals in the Dunedin Study. Reliabilities were highly correlated (*r* = 0.77), suggesting that unreliable probe measurement is systematic.

<u>1.2 Probe-specific characteristics are related to reliability</u>. Next, we tested if probe reliability was related to the mean and variance of methylation levels (β-values) at the site measured by the probe. Our analysis revealed three findings. First, probe-reliability had an inverse-U shaped relationship with mean β-values; the lowest-reliability probes were concentrated at either end of the distribution of methylation β-values (i.e. among hyper- and hypo-methylated probes), whereas the highest reliability probes were concentrated in the intermediate range of the distribution (**Figure S4A**). Second, the highest density of low reliability probes was found among probes with low β-value SD (**Figure S4B**). Third, β-value means and SDs were correlated (*r* = 0.15, *P*<0.01), and the most reliable probes were those with intermediate levels of methylation that varied most between individuals (**Figure S4C**). These observations confirm earlier reports of differential reliability as a function of site-specific characteristics[2-4].

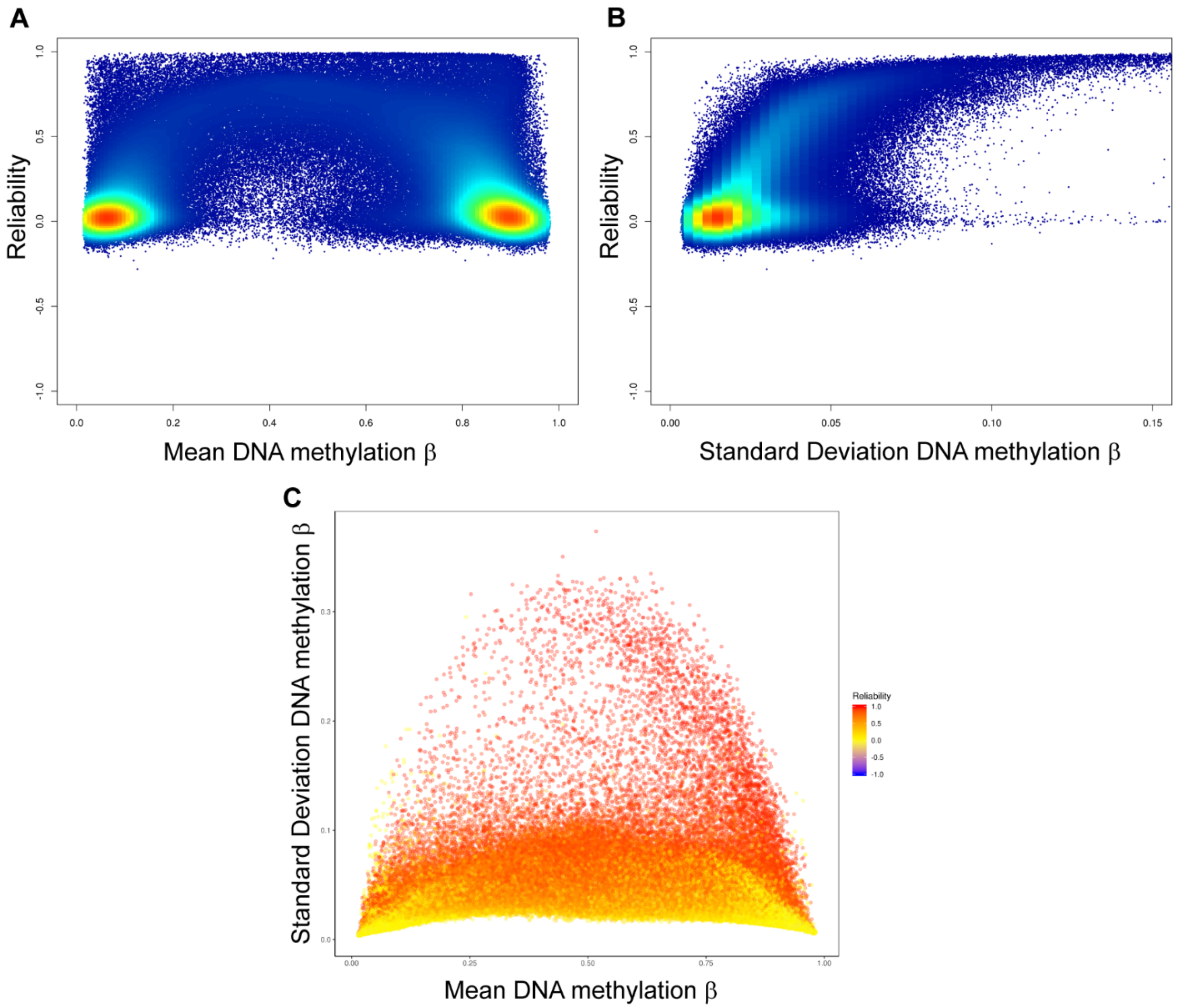

Figure S4: Probe-specific characteristics are related to the distribution of probe reliability. (A) shows a density heatmap of mean DNA methylation level (methylation β, range = 0-1; x-axis) plotted against reliability (Y-axis). This distribution follows an inverted U-shaped curve, where lowest reliabilities tend to be observed where mean β levels are close to either extreme, whereas the highest reliability probes were concentrated in the intermediate range of the distribution. **(B)** shows a density heatmap of the standard deviations of DNA methylation (x-axis) plotted against reliability (Y-axis). Lowest reliabilities tend to be observed where variation in β-levels is the lowest. **(C)** shows means (x-axis) and standard deviations (y-axis) of methylation β-values plotted as a function of reliability (color; red = highest, blue = lowest). Methylation β-level means and SDs are correlated (r=0.15, P<0.01) and show an inverse-U relationship with variability; the most variable probes tend to have mean levels of methylation around the center of the distribution. These variable, intermediately-methylated probes also tend to be most reliable.

1.3 Genomic annotation of probes is related to differential reliability. **Figure S5A** shows that there are regional differences in the distribution of probe reliability (**Data S1**). The transcription start site (TSS) had the highest aggregation of unreliable probes; the intergenic region had the lowest. In addition, CpG islands had a higher aggregation of unreliable probes than CpG shores (**Figure S5B**), a pattern consistent with previous reports[4,5]**.** This could be due to the fact that sites within CpG islands are more likely to be unmethylated[6] and are therefore more likely to be unreliably measured, or it could be because the proportion of Type I Infinium probes in CpG islands is greater than in CpG shores[7] (vs. Type II; the two probe types differ in the chemistry used to quantify methylation level), and Type I probes are more unreliable than Type II[4,5] (**Figure S5C**).

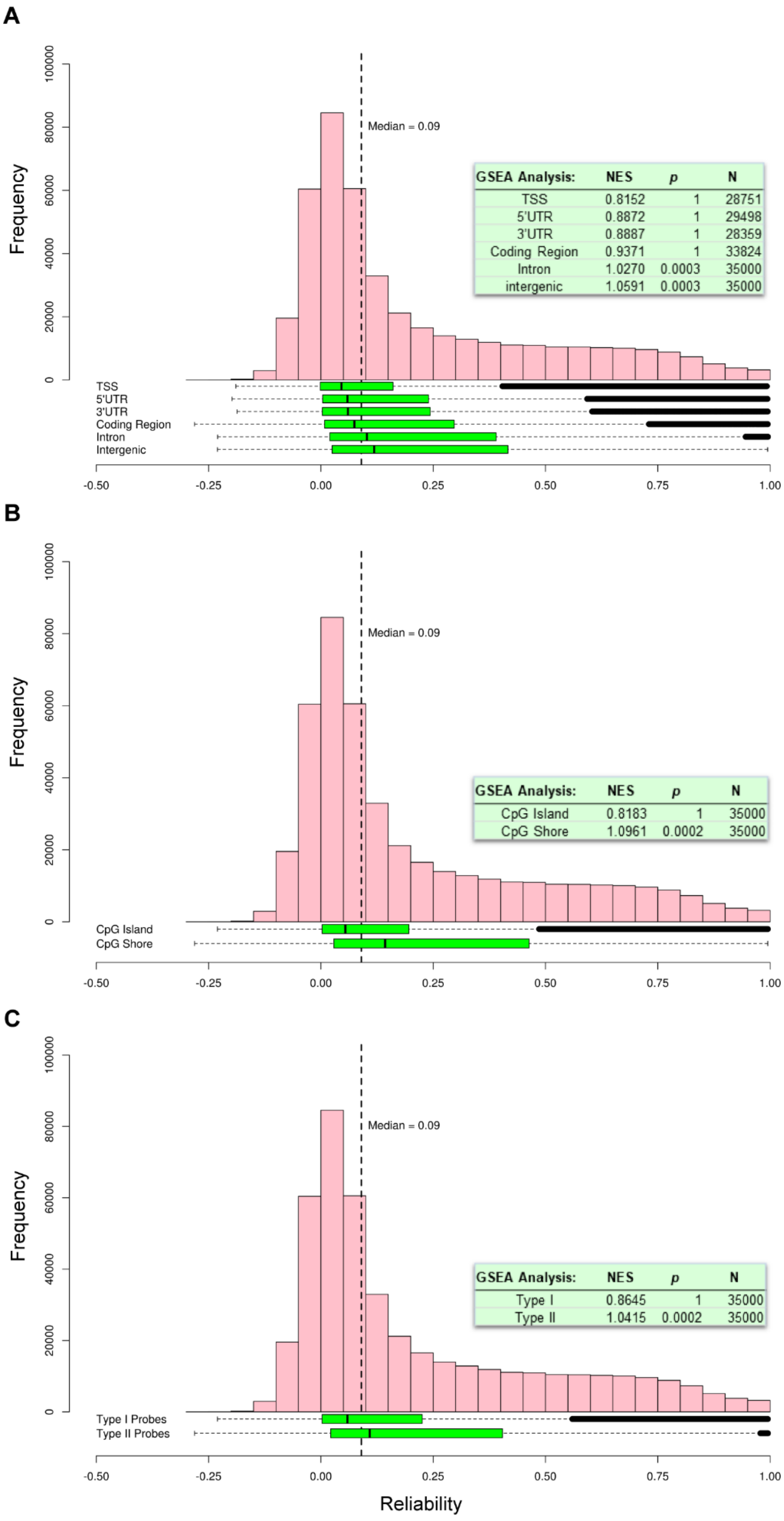
A
Frequency
Median = 0.09
GSEA Analysis: NES p N
TSS 0.8152 1 28751
5'UTR 0.8872 1 29498
3'UTR 0.8887 1 28359
Coding Region 0.9371 1 33824
Intron 1.0270 0.0003 35000
intergenic 1.0591 0.0003 35000
TSS
5'UTR
3'UTR
Coding Region
Intron
Intergenic
B
Frequency
Median = 0.09
GSEA Analysis: NES p N
CpG Island 0.8183 1 35000
CpG Shore 1.0961 0.0002 35000
CpG Island
CpG Shore
C
Frequency
Median = 0.09
GSEA Analysis: NES p N
Type I 0.8645 1 35000
Type II 1.0415 0.0002 35000
Type I Probes
Type II Probes
Reliability

Figure S5: Reliabilities of probes as a function of spatial characteristics. (A) plots the distributions of reliability coefficients as box and whisker plots for probes annotated to one of six genic regions: transcription start site (TSS), 5' untranslated region (5'UTR), 3' untranslated region ('3UTR), coding region, intronic region, and intergenic region. Boxes correspond to Inter-quartile range (IQR), and whiskers extend to 1.5 * IQR. Observations beyond the whiskers (outliers) are represented by individual points. The TSS has the greatest proportion of unreliable probes, the intergenic region the least. **(B)** shows the distribution of reliability coefficients for probes localized to CpG islands or CpG shores. Unreliable probes are more common in CpG islands than CpG shores. Also shown is the distribution of reliability correlations as a function of Infinium probe type; older Type I probes are less reliable than Type II probes **(C).** This could be due to the fact that sites within CpG islands are more likely to be unmethylated and are therefore more likely to be unreliably measured, or it could be because the proportion of Type I Infinium probes in CpG islands is greater than in CpG shores (vs. Type II; the two probe types differ in the chemistry used to quantify methylation level), and Type I probes are more unreliable than Type II. As a reference, the distribution (pink bars) and median (vertical dashed line) of all ~440,000 probe reliabilities in the E-Risk reliability dataset are shown above the box and whisker plots. The text box shows the results of Gene Set Enrichment Analysis (GSEA) for the each set of features; NES= Normalized Enrichment Score, *p*= p-value, N = number of probes. NESs greater than 1 indicate enrichment for reliable probes.

1.4: Low reliability is not artefactual. Previous methodological studies have drawn attention to three factors that might compromise the quality of methylation BeadChip data: probe invariance[8-10], potential probe hybridization problems[11], and skewness. We tested whether these features are sufficient to capture unreliability. They are not. **Figure S6A** and **S6B** document that probe unreliability exists in probes that are variable, and do not have potential probe hybridization problems. **Figure S6C** demonstrates that probe reliabilities calculated on β-values resemble the reliabilities of M-values, a method for transforming skewed probe distributions[12].

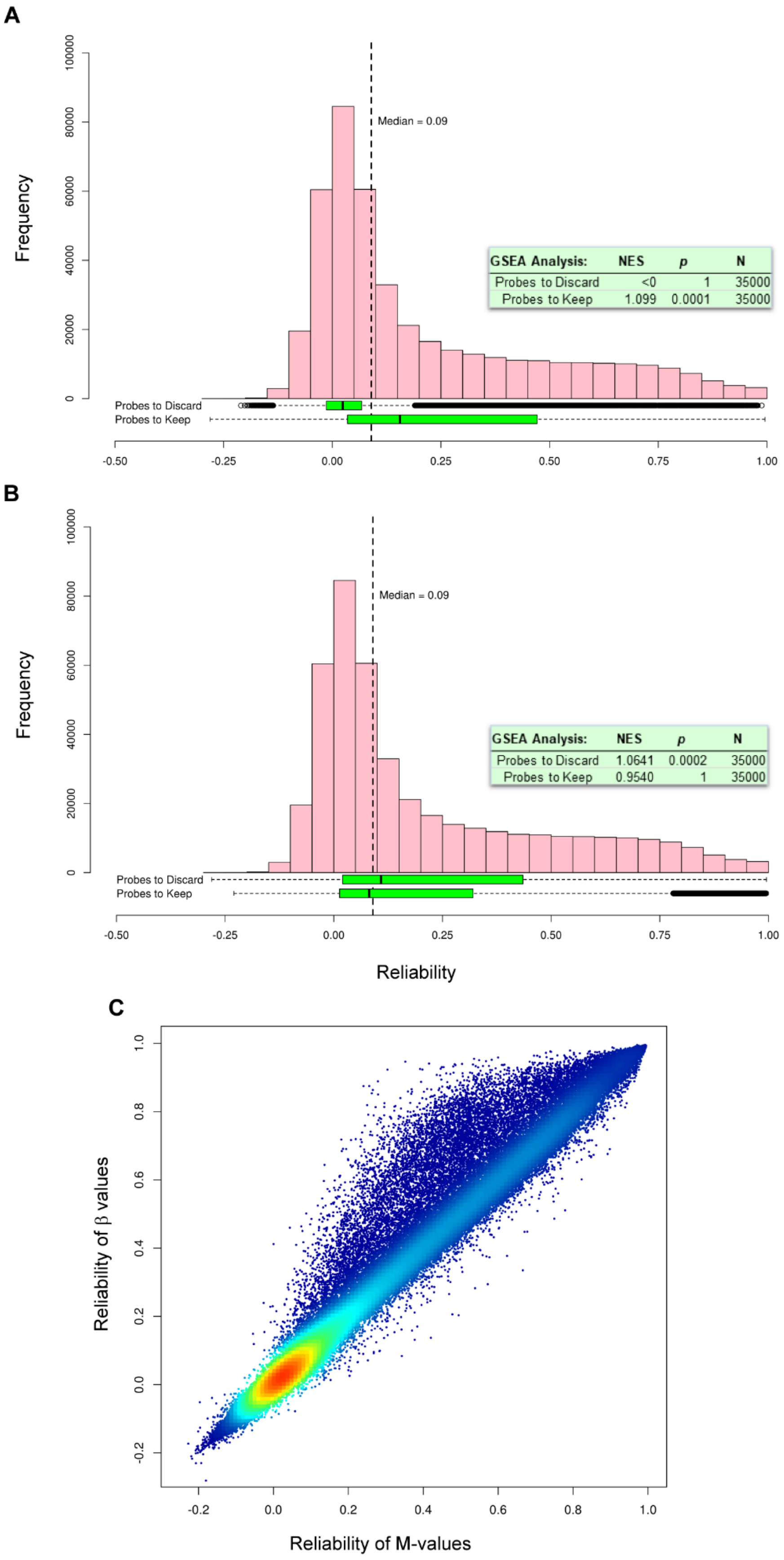
A
Frequency
Median = 0.09
GSEA Analysis: NES p N
Probes to Discard <0 1 35000
Probes to Keep 1.099 0.0001 35000
Probes to Discard
Probes to Keep
B
Frequency
Median = 0.09
GSEA Analysis: NES p N
Probes to Discard 1.0641 0.0002 35000
Probes to Keep 0.9540 1 35000
Probes to Discard
Probes to Keep
Reliability
C
Reliability of β values
Reliability of M-values

Figure S6: The distribution of reliabilities of probes identified as potentially problematic in previous studies. Distributions are depicted as box and whisker plots of the reliability coefficients of the probes identified as variant/invariant by Edgar *et al.* (**A;** probes to discard are invariant probes) or having potential hybridization problems as described by Naaem *et al.* (**B;** probes to discard are probes with hybridization problems). Boxes correspond to Inter-quartile range (IQR), and whiskers extend to 1.5 * IQR. Observations beyond the whiskers (outliers) are represented by individual points. Both variant and non-problematic probe lists ('probes to keep') contain unreliable probes, suggesting these factors alone are not sufficient to index reliability. As a reference, the distribution (pink bars) and median (vertical dashed line) of all ~440,000 probe reliabilities in the E-Risk dataset are shown above the box and whisker plots. The text box shows the results of Gene Set Enrichment Analysis (GSEA) for the suggested set of probes to keep or discard in each situation (NES= Normalized Enrichment Score, *p*= p-value, N = number of probes). NESs greater than 1 indicate enrichment for reliable probes. **(C)** compares the reliability of probes computed using β values against those using M-values. Transforming β values to M-values has little effect on estimates of reliability. These three methods of accounting for unreliable probe data are not fully satisfactory.

In summary, we replicated previous reports of low reliability across probes common to the 450K and EPIC BeadChips, demonstrating that, paradoxically, poor reliability is reproducible. Moreover, factors commonly thought to account for unreliability (such as genomic location, invariance and skewness) do not provide a satisfactory account of its ubiquity.

Section S2: Testing the sensitivity of associations with reliability in light of probe variability

This section relates to Main Text Discussion Section:

'Approaches to improve replicability via reliability assessment.'

We demonstrated that probe reliability is related to various properties of probe measurements (e.g. probe variability, **section S1.2** above). These observations might lead one to ask: are these properties the major drivers of reliability, such that it is unreasonable to assess reliability without their adequate consideration?

We tested this assumption using variability as a case in point. Our reasoning was that If variability is the major driver of reliability, then it follows that exclusion of invariant probes should increase the power to detect associations between reliability and the factors we outline in the main text of the manuscript. We subset our data to only those probes identified as not invariant in blood by Edgar *et al.*[8]. We then repeated our analysis of a) the association between probe reliability and estimates of genetic and environmental influences on DNA methylation, b) the association with mQTL probes, and c) the association with the extent of concordance in DNA methylation levels between blood and brain tissue.

We first tested if the probes identified as invariant by Edgar *et al.*[8] had the same distribution of reliabilities as probes that we independently determine as invariant within our own data. As shown in **Figure S7** (below), the overlap of reliabilities of the probes listed by Edgar *et al.*[8] and probes identified within our data is very high, suggesting that characteristics of individual probes (such as probe variance) are highly reproducible and unlikely to result from experimental-specific artifacts. As such, we went forward to subset our data to only those probes that were not invariant (i.e. 'variant') and repeated our tests of association outlined above.

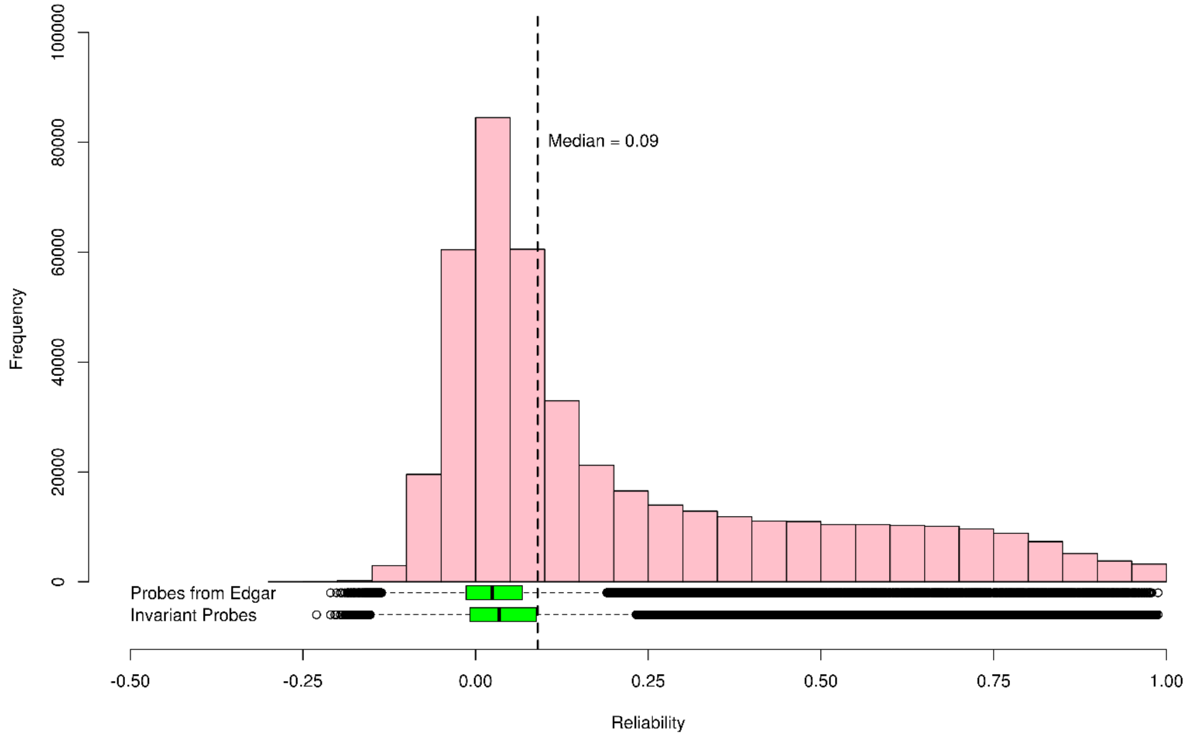

Figure S7. Comparison of reliabilities of invariant probes. Distributions are depicted as box and whisker plots of the reliability coefficients of the probes identified as invariant by Edgar *et al.,* (top box) or identified as invariant based on our own data (bottom box). Boxes correspond to Inter-quartile range (IQR), and whiskers extend to 1.5 * IQR. Observations beyond the whiskers (outliers) are represented by individual points. The distribution of reliability in both sets of invariant probes are similar, suggesting the lists are highly conserved across studies. As a reference, the distribution (pink bars) and median (vertical dashed line) of all ~440,000 probe reliabilities in the E-Risk dataset are shown above the box and whisker plots.

2.1: Associations between probe reliability and estimates of genetic and environmental influences on DNA methylation. In our manuscript, we report that estimates of additive genetic variation were positively correlated with reliability, and estimates of non-shared environmental variation (which also inlcudes measurement error) were negatively associated with reliabilty.

When restricting analysis to just those probes that are variable, we find little attenuation of the association between reliability and these estimates (**Table S1**, below). It is not purely variability driving the associations, since excluding invariant probes does not improve the power to detect associations.

Table S1. Correlations of reliability and ACE parameters

	**All probes (*N* = 430,802)**		**Variant probes only (*N* = 292,127)**	
	r	95% CI	*r*	95% CI
**Additive genetic variation (A)**	0.702	0.701, 0.0704	0.705	0.703, 0.706
**Shared environmental variation (C)**	-0.073	-0.076, -0696	-0.039	-0.042, -0.035
**Non-shared environmental variation (E)**	-0.583	-0.584, -0.5805	-0.657	-0.659, -0.655

<u>2.2: Associations between probe reliability and mQTL probes</u>. In our manuscript, we report that methylation Quantitative Trait Loci (mQTLs)--DNA sequence variants that are associated with differential DNA methylation--are more likely to be associated with reliable probes than unreliable probes.

When restricting our analysis to just those probes that are variable, we find little change in the extent to which the list of mQTL-associated probes is enriched for reliable probes (**Table S2**, below). It is not purely variability driving the ability to detect associations between sequence variants and differential DNA methylation.

Table S2. **GSEA (enrichment) analysis of mQTL- and non mQTL indexing probes**

	**All probes (*N* = 438,593)**		**Variant probes only (*N* = 334,449)**	
	Normalized Enrichment Score	*p* value	Normalized Enrichment Score	*p* value
**mQTL probes**	1.477	0.002	1.525	0.0002
**non-mQTL probes**	0.867	1.00	0.850	1.00

<u>2.3: Associations of probe reliability with the extent of concordance in DNA methylation levels between blood and brain tissue.</u> In our manuscript, we report that probes that show similar levels of DNA methylation in blood and any of four different brain regions ('blood-brain' concordance) are more likely to be reliably measured.

When restricting our analysis to just those probes that are variable, we find little attenuation of the association between reliability and blood-brain concordance (**Table S3**, below). It is not purely variability driving the ability to detect blood-brain concordance.

Table S3. correlations of reliability and concordance of methylation values between blood and each of four brain regions

	**All probes (*N* = 438,593)**		**Variant probes only (*N* = 334,449)**	
**Blood-brain region concordance**	*rho*	95% CI	*rho*	95% CI
**Prefrontal Cortex**	0.348	0.345, 0.351	0.362	0.359, 0.365
**Entorhinal Cortex**	0.315	0.312, 0.317	0.360	0.357, 0.363
**Superior Temporal Gyrus**	0.376	0.373, 0.379	0.390	0.387, 0.393
**Cerebellum**	0.218	0.215, 0.222	0.218	0.215, 0.221

In summary, variability, though highly related to reliability, is not sufficient to account for the challenges posed by unreliable DNA methylation measurement.

Section S3: Additional Experimental Procedures

S3.1: Sample description and data production

Environmental Risk (E-Risk) Longitudinal Twin Study

Sample Description. Participants were members of E-Risk, which tracks the development of a 1994-95 birth cohort of 2,232 British children[13]. Briefly, the E-Risk sample was constructed in 1999-2000, when 1,116 families (93% of those eligible) with same-sex 5-year-old twins participated in home-visit assessments. This sample comprised 56% monozygotic (MZ) and 44% dizygotic (DZ) twin pairs; sex was evenly distributed within zygosity (49% male). The study sample represents the full range of socioeconomic conditions in Great Britain, as reflected in the families' distribution on a neighborhood-level socioeconomic index (ACORN [A Classification of Residential Neighbourhoods], developed by CACI Inc. for commercial use): 25.6% of E-Risk families live in "wealthy achiever" neighborhoods compared to 25.3% nationwide; 5.3% vs. 11.6% live in "urban prosperity" neighborhoods; 29.6% vs. 26.9% in "comfortably off" neighborhoods; 13.4% vs. 13.9% in "moderate means" neighborhoods; and 26.1% vs. 20.7% in "hard-pressed" neighborhoods. E-Risk underrepresents "urban prosperity" neighborhoods because such households are often childless.

Home visits were conducted when participants were aged 5, 7, 10, 12 and most recently, 18 years (93% participation). The Joint South London and Maudsley and the Institute of Psychiatry Research Ethics Committee approved each phase of the study. Parents gave informed written consent and twins gave written assent between 5-12 years and then informed written consent at age 18.

At age 18, 2,066 participants were assessed, each twin by a different interviewer. The average age at the time of assessment was 18.4 years (SD = 0.36); all interviews were conducted after the 18th birthday.

Genome-wide quantification of DNA methylation. Our epigenetic study used DNA from a single tissue: blood. At age 18, whole blood was collected from 82% (N=1700) of the participants in 10mL K_2EDTA tubes. DNA was extracted from the buffy coat using a Flexigene DNA extraction kit (Qiagen, Hilden, Germany) following manufacturer's instructions. Study members who did not provide blood provided buccal swabs, but these were not included in our methylation analysis to avoid tissue-source confounds. Assays were run by the Complex Disease Epigenetics Group at the University of Exeter Medical School, and as described in full in previous publications[9,14]. 450K BeadChip data were available for 1658 study members.

Reliability dataset. For our reliability analysis we selected 350 individuals to assay with the EPIC BeadChip. ~500ng of DNA from each sample was treated with sodium bisulfite using the EZ-96 DNA Methylation kit (Zymo Research, CA, USA). DNA methylation was quantified using the Infinium MethylationEPIC ('EPIC') BeadChip run on an Illumina iScan System (Illumina, CA, USA) by the Complex Disease Epigenetics Group at the University of Exeter Medical School.

Reliability Dataset Processing and Normalization. The EPIC and 450K BeadChip data that comprise the reliability dataset were imported into the minfi Bioconductor package[15,16]. Probes were excluded if they had a detection p-value > 0.05 in at least 10% of the samples in either the EPIC or the 450K BeadChip datasets. Data were processed using the subset-quantile within array normalization

(SWAN) approach to eliminate systematic differences across the arrays. This method was chosen because it is currently one of the very few methods that allows normalization of 450K and EPIC BeadChip data together. Probes were kept for subsequent analysis if they passed the detection p-value threshold in both technologies, were shared between the two array platforms, and did not map to a sex chromosome.

Low reliability might arise through experimental factors not related solely to poor probe performance. We therefore tested two ways in which normalization might affect reliability estimates. First, low reliability could be due to data handling differences between datasets. To test this, we compared reliability coefficients after normalizing the datasets in two ways: (a) where data from 450K and EPIC BeadChips were normalized as separate datasets and (b) where they were normalized together as one dataset. The different normalization strategies had little effect on reliability estimates (**Figure S8A,** r = 1.00, $p < 0.01$), suggesting differential probe reliability was not a product of data-handling practices. The 'normalized separately' set is used for all analyses unless otherwise noted.

Second, low reliability could be due to differences in relative ranks of probes induced through use of specific normalization methods. To test this, we re-normalized our data using an alternative method (''Quantile") to that we have employed ("SWAN"), and compared the reliabilities generated using each. Normalization method had little effect on reliability measures (**Figure S8B**, r = 0.98, $p < 0.01$), suggesting our results are not affected by normalization strategy.

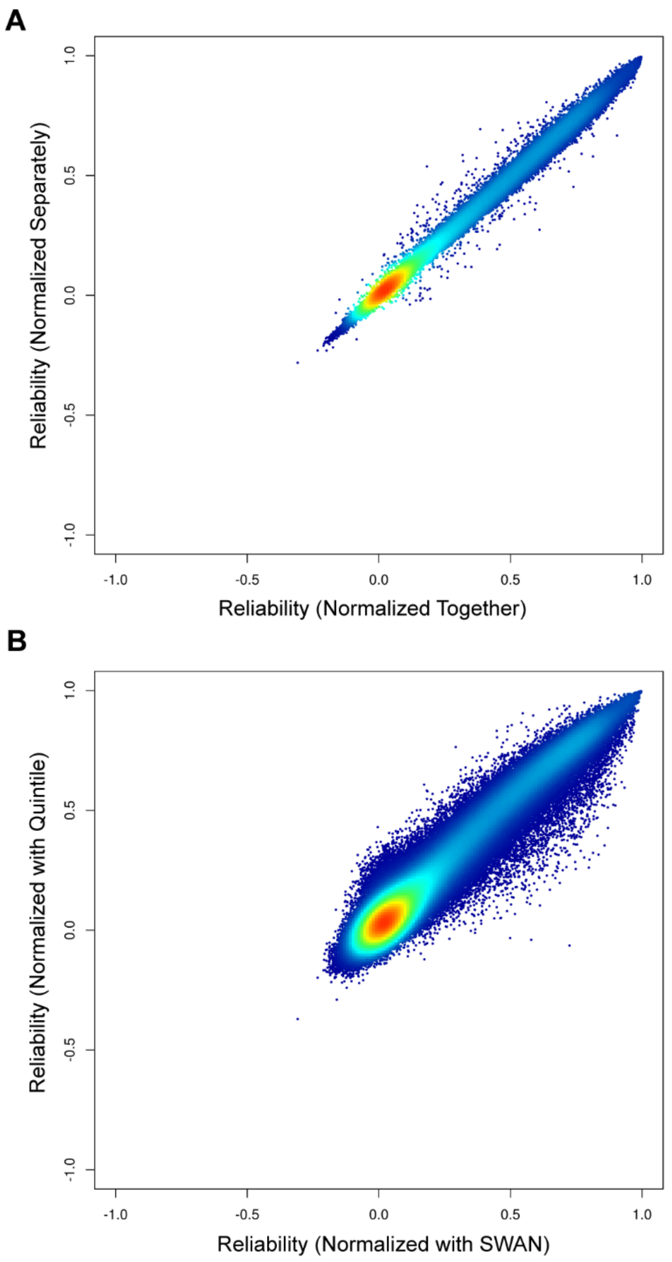

Figure S8: Reliability correlations for probes common to the 450K and EPIC BeadChips. (A) compares the reliability correlations generated when data for each BeadChip type were normalized together (x-axis) or normalized separately (y-axis). **(B)** compares the reliability correlations generated using ‘SWAN’, as reported in the main text of the manuscript (x-axis), and those generated using data normalized with ‘Quantile’ (y-axis). In either case, normalization strategy seems to have little effect on the distribution of probe-probe reliability correlations.

Dunedin Longitudinal Study

Sample description. Participants were members of the Dunedin Multidisciplinary Health and Development Study, a longitudinal investigation of health and behavior in a representative birth cohort[17]. Study members (n = 1,037; 91% of eligible births; 52% male) were all individuals born between April 1972 and March 1973 in Dunedin, New Zealand, who were eligible for the longitudinal study based on residence in the province at 3 years of age and who participated in the first follow-up assessment at 3 years of age. The cohort represented the full range of socioeconomic status on NZ's South Island. On adult health, the cohort matches the NZ National Health and Nutrition Survey (e.g., BMI, smoking, GP visits)[17]. The cohort is primarily white (93%); genetic analyses were restricted to non-Maori participants. Assessments were carried out at birth and at ages 3, 5, 7, 9, 11, 13, 15, 18, 21, 26, 32, 38 and 45 years, when 94% of the 997 study members still alive took part. The Otago Ethics Committee approved each phase of the study and informed consent was obtained from all study members.

Genome-wide quantification of DNA methylation using 450K BeadChips. Our epigenetic study used DNA from a single tissue: blood. Whole blood was collected in 10mL K_2EDTA tubes from N = 857 participants at age 38. DNA was extracted from the buffy coat using standard procedures[18,19]. Study members who did not provide blood provided buccal swabs, but these were not included in our methylation analysis to avoid tissue-source confounds.

We assayed 835 blood samples (out of 857); 22 samples were not useable. ~500ng of DNA from each sample was treated with sodium bisulfite using the EZ-96 DNA Methylation kit (Zymo Research, CA, USA). DNA methylation was quantified using the Illumina Infinium HumanMethylation450 BeadChip ("Illumina 450K BeadChip") run on an Illumina iScan System (Illumina, CA, USA) at the Molecular Genomics Core at the Duke Molecular Physiology Institute and are described in full in previous publications[14].

Genome-wide quantification of DNA methylation using EPIC BeadChips. To assay within-array reliability of the EPIC BeadChip, we selected 28 individuals from the Age 45 data collection phase of the Dunedin Study and assayed their DNA twice. DNA was collected from blood and extracted as above. ~500ng of DNA from each sample was treated with sodium bisulfite using the EZ-96 DNA Methylation kit (Zymo Research, CA, USA). DNA methylation was quantified using the Infinium MethylationEPIC ('EPIC') BeadChip run on an Illumina iScan System (Illumina, CA, USA) at the Molecular Genomics Core at the Duke Molecular Physiology Institute. Data were processed, underwent quality control filtering, and normalized as described above for the 350-sample reliability dataset.

Gene Expression. Expression data were generated from whole-blood RNA using the Affymetrix PrimeView Human Gene Chip (Affymetrix, CA, USA). Briefly, these arrays simultaneously interrogate more than 38,000 gene transcripts across the entire genome. Whole-blood RNA samples collected via PaxGene Blood RNA tubes (Qiagen, CA, USA) at age 38 were assayed. Samples were arranged into batches of 60. Array processing was performed by the Duke University Microarray Core Facility using the Affymetrix GeneChip system (Affymetrix). Prior to hybridization, total RNA was assessed for quality with Agilent 2100 Bioanalyzer G2939A (Agilent Technologies, Santa Clara, CA)) and Nanodrop 8000 spectrophotometer (Thermo Scientific/Nanodrop, Wilmington, DE). Samples with RIN ≥ 6 were then subject to globin mRNA depletion using the GLOBINClear –human kit (Ambion, Thermo Fisher Scientific, MA, USA). RNA samples from 843 individuals were assayed. Data quality control and RMA normalization were carried out using the *affy* Bioconductor package[20] in the R statistical programming environment. After QC, expression data were available for 836 individuals.

S3.2: Data analysis

Data analysis was performed in the R statistical programming environment, often using Bioconductor packages. Data handling was performed using the package *dplyr* [21] and descriptives were generated using the package *psych*[22]. Plots were produced in R using the packages *ggplot2*[23] and *ggpubr*[24] where appropriate. Density heatmaps were generated using the *KernSmooth* package[25]. Unless otherwise noted, correlations are reported as two-tailed Pearson product-moment correlation coefficients. Intraclass correlation was calculated using the *irr* package[26].

Probe reliabilities. Probe reliabilities are computed using Intraclass Correlations (ICC), calculated for each autosomal probe present on both the EPIC and 450K BeadChip (*N*=438,593). ICCs are an oft-used metric to assess reliability in test-retest situations[27], and many different models exist depending on the way in which the test-retest data are generated. Here, we calculated ICCs based on a mean-rating (k=2), absolute-agreement, 2-way random-effects model. We chose this model using the guidelines outlined in Koo and Li[27], where mean-rating (k=2) relates to the number of repeated measures (i.e., BeadChips per sample); absolute agreement requires that not only do the values across BeadChips correlate, but that values are in agreement; and 2-way random effects relates to the generalizability of the ICCs to any subsequent similarly characterized rater (where rater = BeadChip probe). To compare whether test-retest model choice had an effect on reliability estimates, we also computed Pearson product-moment correlation coefficients. Pearson correlation coefficients and ICC estimates of reliability were highly similar (r=1.00, P=<1x10^{-4}; **Figure S9**).

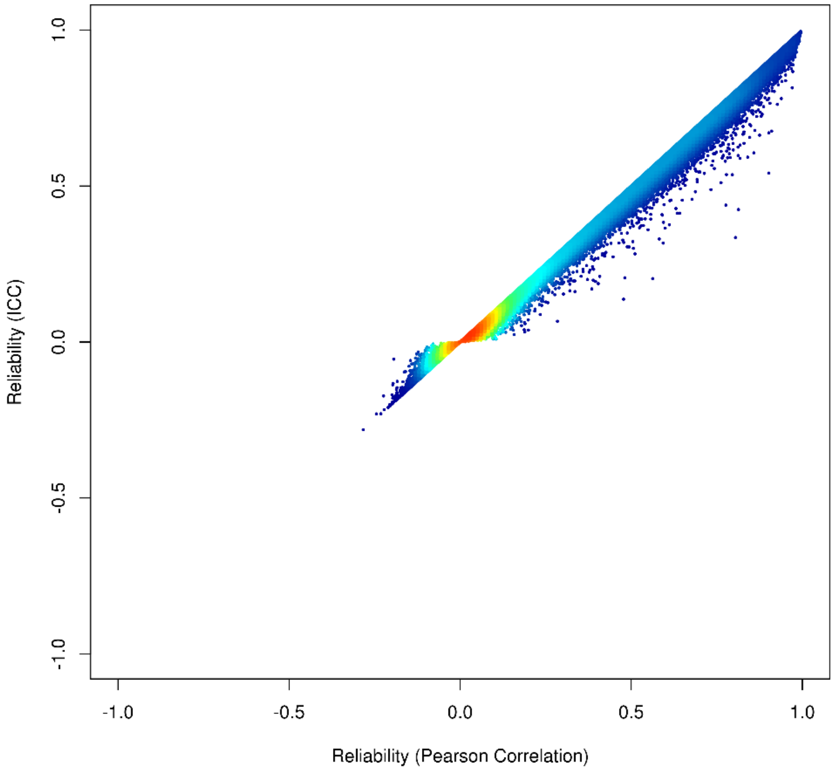

Figure S9: Reliabilities expressed as Pearson correlation coefficients and Intra-Class Coefficients are similar (Refers to Main Text Experimental Procedures section "Probe Reliabilities"). The y-axis plots probe reliabilities as Pearson correlation coefficients and the x-axis plots the Intra-Class Coefficients (ICC. Reliabilities were highly correlated (r = 1.00).

Gene Set Enrichment Analysis. Gene Set Enrichment Analysis (GSEA) was performed using the *fgsea* Bioconductor package[28] with 10,000 permutations. We tested if each probe list was significantly enriched for more highly reliable probes. Due to computing constraints, if a list had more than 35000 probes, it was truncated down to a random sampling of 35000 probes for the analysis.

Structural equation modelling. Biometrical modelling was applied to every probe passing QC on the Illumina 450K array. Specifically, an ACE model was fitted to calculate the proportion of variance in DNA methylation explained by additive genetic (A), shared environmental (C) and unshared or unique environmental (E) factors, the latter which also includes measurement error. The assumptions behind this model are that additive genetic factors are perfectly correlated between MZ twins (i.e. genetic correlation = 1) but are only 50% correlated between DZ twins (i.e. genetic correlation = 0.5) and that shared non-heritable influences are equally similar between MZ and DZ twin pairs. The model was fitted using structural equation modelling implemented with functions from the *OpenMx* R package[29,30].

Identification of Smoking-related DNA methylation probes. We identified 22 studies that reported an epigenome-wide analysis of current vs never smoking using the 450K BeadChip platform[31-37]. For each study, we obtained lists of probe IDs and direction-of-effect for probes that were significantly associated with current smoking (as determined by the study authors; total number of probes=3,724; *N* probes per study=84-2,441). We then determined the extent to which individual probes replicated across the 22 studies by summing the number of times each probe was listed with consistent direction-of-effect (i.e., consistent cross-study increases or decreases in methylation in response to smoking). Descriptions of the studies included are found in **Table S4**.

Table S4. Descriptions of the studies included in analysis of consistency of replication for DNA methylation-smoking associations (Refers to Main text Result item "*Probe reliability impacts association testing*"). Descriptives are derived from the original publications. Information on the 16 studies included in the meta-analysis by Joehanes *et al.,* (2016) is individually listed.

Publication	*Cohort*	*Sample Origin*	*N (% smokers)*	*% male*	*Age; mean (SD), where available*	*N probes significant**	*N probes with available reliability data*	*Additional Notes*
**Zellinger *et al.,* (2013)**	KORA F3 and F4	Whole Blood	1011 (26.0) and 468 (50.4)	60.3 and 49.4	56.96 (46-76)	187	174	sites replicated across F3 and F4
**Besingi *et al.,* (2014)**	NSPHS	Whole Blood	421 (10.2)	53.0	14 - 94	95	84	
**Dogan *et al.,* (2014)**	FACHS	PBMCs	111 (45.0)	0.0	48.1 +/- 7	910	840	African American participants
**Guida *et al.,* (2015)**	EPIC and NOWAC	Buffy coat	745 (23.8)	0.0	53.1 (7.4); 55.4 (4.3)	461	431	
**Dogan *et al.,* (2017)**	FHS	Buffy coat	1597 (7.6)	54.9	62.0 - 67.7 (6.5- 8.6)	525	482	current vs non-smoker
**Wilson *et al.,* (2017)**	KORA S4/F4	whole blood	1344 (20.38)	58.1	50.8 (7.8) - 55.1 (9.0)	590	557	
**Joehanes *et al.,* (2016); meta-analysis comprising 16 cohorts (listed individually); each cohort treated as an individual study for current analysis**						2,623**	2,441	
	ARIC	Buffy coat	2848 (25.3)	36.4	56.2 (5.8)			African American participants
	GTP	Whole Blood	286 (32.9)	29.0	43.4 (11.7)			African American participants
	CHS AA	Whole Blood	192 (15.6)	34.9	70.4 (4.9)			African American participants
	GENOA	Buffy coat	420 (18.3)	28.8	58.7 (7.9)			African American participants
	FHS	Whole Blood	2648 (10.3)	45.7	62.5 (7.8)			European American participants
	KORA F4	Whole Blood	1797 (14.6)	48.7	57.0 (7.0)			European American participants
	GOLDN	CD4+	992 (7.4)	47.8	44 (13)			European American participants
	LBC 1921	Whole Blood	445 (7.0)	39.6	79.2 (0.5)			European American participants

Publication	***Cohort***	***Sample Origin***	***N (% smokers)***	***% male***	***Age; mean (SD), where available***	***N probes significant****	***N probes with available reliability data***	***Additional Notes***
	LBC 1936	Whole Blood	920 (11.2)	50.5	69.5 (0.7)			European American participants
	NAS	Whole Blood	644 (4.0)	100.0	68.2 (6.1)			European American participants
	Rotterdam	Whole Blood	686 (24.6)	43.6	58.0 (6.8)			European American participants
	Inchianti	Whole Blood	508 (9.8)	45.1	58.9 / 16.8			European American participants
	CHS EA	Whole Blood	184 (12.5)	44.0	74.1 (4.2)			European American participants
	EPIC-Norfolk	Buffy coat	1183 (16.1)	49.6	58.3 (8.4)			European American participants
	MESA	CD14+	1256 (9.1)	48.6	65 (8)			European American, African American and Hispanic participants
	EPIC	Buffy coat	898 (21.8)	0.0	48.9 (8.8)			European American participants

** as identified by Study Authors*
***significant at α = 1×10^{-7} level*

Correlation of methylation with gene expression. Each probe in the Dunedin 450K methylation dataset was correlated with each probeset from the Dunedin PrimeView gene expression dataset using Spearman's rank correlation approach. To control for technical variation in the gene expression data, we regressed out the following microarray-based quality metrics described by Peters *et al.*[38]: mean of positive match probesets, mean of positive control probesets, standard deviation of positive control probesets, mean of negative control probesets, standard deviation of negative control probesets, mean of all probesets, standard deviation of all probesets, and relative log expression mean of all probesets, along with sex, array batch and RIN. To control for technical variation in the methylation data, we regressed out the first 32 principal components calculated from the control probes on the arrays. For both datasets, we controlled for cell type composition by regressing out white cell-type counts measured using flow cytometry (Sysmex Corporation, Japan) in whole blood samples taken concurrently with the DNA and RNA samples. Methylation probes that overlapped the transcription start site of at least one isoform of each gene represented by a gene expression probeset were kept for subsequent analysis. For each methylation probe, the gene expression probeset with the highest Spearman correlation coefficient was retained as the representative probeset for the expression level of that gene. Thus, each methylation probe is reported as correlated with a single gene expression probeset. A methylation-expression correlation coefficient was considered significant if it had a *p*-value <= $1x10^{-7}$.

Determination of the number of replicates needed to identify reliable probes. The 350 samples used for the reliability analysis were randomly ordered. Reliability was calculated on growing subsets of the data that were needed to consistently identify probes that had a reliability >= 0.75 in the full set of 350 samples (**Figure S10**).

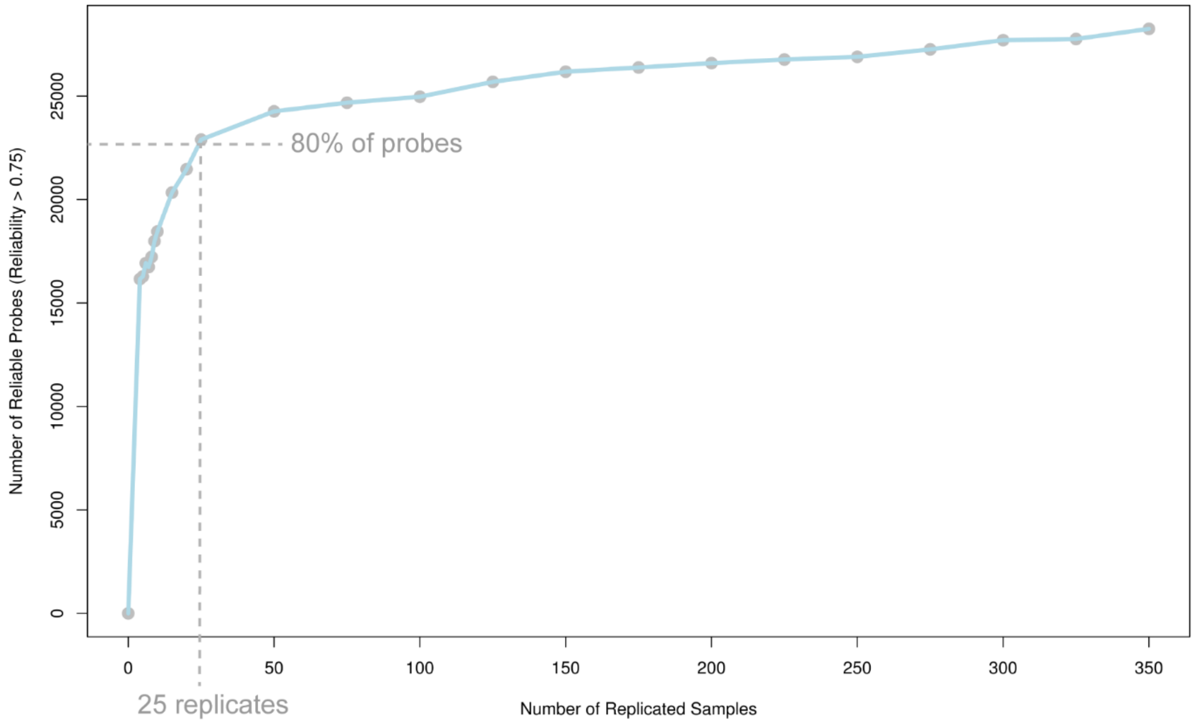

Figure S10: Simulation of the number of replicate BeadChips needed to identify reliable probes. Simulations suggests that 25 replicates would be sufficient to capture 80% of the probes with reliability > 0.75 observed in the dataset of 350.

References

1. Cicchetti, D.V., and Sparrow, S.A. (1981). Developing criteria for establishing interrater reliability of specific items: applications to assessment of adaptive behavior. Am J Ment Defic 86, 127-137.

2. Logue, M.W., Smith, A.K., Wolf, E.J., Maniates, H., Stone, A., Schichman, S.A., McGlinchey, R.E., Milberg, W., and Miller, M.W. (2017). The correlation of methylation levels measured using Illumina 450K and EPIC BeadChips in blood samples. Epigenomics 9, 1363-1371.

3. Dugue, P.A., English, D.R., MacInnis, R.J., Jung, C.H., Bassett, J.K., FitzGerald, L.M., Wong, E.M., Joo, J.E., Hopper, J.L., Southey, M.C.*, et al.* (2016). Reliability of DNA methylation measures from dried blood spots and mononuclear cells using the HumanMethylation450k BeadArray. Sci Rep 6, 30317.

4. Bose, M., Wu, C., Pankow, J.S., Demerath, E.W., Bressler, J., Fornage, M., Grove, M.L., Mosley, T.H., Hicks, C., North, K.*, et al.* (2014). Evaluation of microarray-based DNA methylation measurement using technical replicates: the Atherosclerosis Risk In Communities (ARIC) Study. BMC Bioinformatics 15, 312.

5. Solomon, O., MacIsaac, J., Quach, H., Tindula, G., Kobor, M.S., Huen, K., Meaney, M.J., Eskenazi, B., Barcellos, L.F., and Holland, N. (2018). Comparison of DNA methylation measured by Illumina 450K and EPIC BeadChips in blood of newborns and 14-year-old children. Epigenetics 13, 655-664.

6. Bibikova, M., Le, J., Barnes, B., Saedinia-Melnyk, S., Zhou, L., Shen, R., and Gunderson, K.L. (2009). Genome-wide DNA methylation profiling using Infinium(R) assay. Epigenomics 1, 177-200.

7. Dedeurwaerder, S., Defrance, M., Calonne, E., Denis, H., Sotiriou, C., and Fuks, F. (2011). Evaluation of the Infinium Methylation 450K technology. Epigenomics 3, 771-784.

8. Edgar, R.D., Jones, M.J., Robinson, W.P., and Kobor, M.S. (2017). An empirically driven data reduction method on the human 450K methylation array to remove tissue specific non-variable CpGs. Clin Epigenetics 9, 11.

9. Hannon, E., Knox, O., Sugden, K., Burrage, J., Wong, C.C.Y., Belsky, D.W., Corcoran, D.L., Arseneault, L., Moffitt, T.E., Caspi, A.*, et al.* (2018). Characterizing genetic and environmental influences on variable DNA methylation using monozygotic and dizygotic twins. PLoS Genet 14, e1007544.

10. van Dongen, J., Ehli, E.A., Slieker, R.C., Bartels, M., Weber, Z.M., Davies, G.E., Slagboom, P.E., Heijmans, B.T., and Boomsma, D.I. (2014). Epigenetic variation in monozygotic twins: a genome-wide analysis of DNA methylation in buccal cells. Genes (Basel) 5, 347-365.

11. Naeem, H., Wong, N.C., Chatterton, Z., Hong, M.K., Pedersen, J.S., Corcoran, N.M., Hovens, C.M., and Macintyre, G. (2014). Reducing the risk of false discovery enabling identification of biologically significant genome-wide methylation status using the HumanMethylation450 array. BMC Genomics 15, 51.

12. Du, P., Zhang, X., Huang, C.C., Jafari, N., Kibbe, W.A., Hou, L., and Lin, S.M. (2010). Comparison of Beta-value and M-value methods for quantifying methylation levels by microarray analysis. BMC Bioinformatics 11, 587.

13. Moffitt, T.E., and E-Risk Study Team (2002). Teen-aged mothers in contemporary Britain. J Child Psychol Psychiatry 43, 727-742.

14. Marzi, S.J., Sugden, K., Arseneault, L., Belsky, D.W., Burrage, J., Corcoran, D.L., Danese, A., Fisher, H.L., Hannon, E., Moffitt, T.E.*, et al.* (2018). Analysis of DNA Methylation in Young People: Limited Evidence for an Association Between Victimization Stress and Epigenetic Variation in Blood. Am J Psychiatry 175, 517-529.

15. Aryee, M.J., Jaffe, A.E., Corrada-Bravo, H., Ladd-Acosta, C., Feinberg, A.P., Hansen, K.D., and Irizarry, R.A. (2014). Minfi: a flexible and comprehensive Bioconductor package for the analysis of Infinium DNA methylation microarrays. Bioinformatics 30, 1363-1369.

16. Huber, W., Carey, V.J., Gentleman, R., Anders, S., Carlson, M., Carvalho, B.S., Bravo, H.C., Davis, S., Gatto, L., Girke, T.*, et al.* (2015). Orchestrating high-throughput genomic analysis with Bioconductor. Nat Methods 12, 115-121.

17. Poulton, R., Moffitt, T.E., and Silva, P.A. (2015). The Dunedin Multidisciplinary Health and Development Study: overview of the first 40 years, with an eye to the future. Soc Psychiatry Psychiatr Epidemiol 50, 679-693.

18. Bowtell, D.D. (1987). Rapid isolation of eukaryotic DNA. Anal Biochem 162, 463-465.

19. Jeanpierre, M. (1987). A rapid method for the purification of DNA from blood. Nucleic Acids Res 15, 9611.

20. Gautier, L., Cope, L., Bolstad, B.M., and Irizarry, R.A. (2004). affy--analysis of Affymetrix GeneChip data at the probe level. Bioinformatics 20, 307-315.

21. Wickham, H., François, R., Henry, L., and Müller, K. (2018). dplyr: A Grammar of Data Manipulation. R package version 0.7.4. .

22. Revelle, W. (2018). psych: Procedures for Personality and Psychological Research. R package version 1.7.8.

23. Wickham, H. (2009). ggplot2 (New York, New York, USA: Springer-Verlag New York).

24. Kassambara, A. (2018). ggpubr: 'ggplot2' Based Publication Ready Plots. R package version 0.1.6.9999

25. Wand, M. (2015). KernSmooth: Functions for Kernel Smoothing Supporting Wand & Jones (1995). R package version 2.23-15.

26. Gamer, M., Lemon, J., Fellows, I., and Singh, P. (2019). irr: Various Coefficients of Interrater Reliability and Agreement. R package version 0.84.1.

27. Koo, T.K., and Li, M.Y. (2016). A Guideline of Selecting and Reporting Intraclass Correlation Coefficients for Reliability Research. J Chiropr Med 15, 155-163.

28. Sergushichev, A.A. (2016). An algorithm for fast preranked gene set enrichment analysis using cumulative statistic calculation (bioRxiv).

29. Boker, S., Neale, M., Maes, H., Wilde, M., Spiegel, M., Brick, T., Spies, J., Estabrook, R., Kenny, S., Bates, T.*, et al.* (2011). OpenMx: An Open Source Extended Structural Equation Modeling Framework. Psychometrika 76, 306-317.

30. Neale, M.C., Hunter, M.D., Pritikin, J.N., Zahery, M., Brick, T.R., Kirkpatrick, R.M., Estabrook, R., Bates, T.C., Maes, H.H., and Boker, S.M. (2016). OpenMx 2.0: Extended Structural Equation and Statistical Modeling. Psychometrika 81, 535-549.

31. Dogan, M.V., Beach, S.R.H., and Philibert, R.A. (2017). Genetically contextual effects of smoking on genome wide DNA methylation. Am J Med Genet B Neuropsychiatr Genet 174, 595-607.

32. Besingi, W., and Johansson, A. (2014). Smoke-related DNA methylation changes in the etiology of human disease. Hum Mol Genet 23, 2290-2297.

33. Guida, F., Sandanger, T.M., Castagne, R., Campanella, G., Polidoro, S., Palli, D., Krogh, V., Tumino, R., Sacerdote, C., Panico, S., *et al.* (2015). Dynamics of smoking-induced genome-wide methylation changes with time since smoking cessation. Hum Mol Genet 24, 2349-2359.

34. Joehanes, R., Just, A.C., Marioni, R.E., Pilling, L.C., Reynolds, L.M., Mandaviya, P.R., Guan, W., Xu, T., Elks, C.E., Aslibekyan, S., *et al.* (2016). Epigenetic Signatures of Cigarette Smoking. Circ Cardiovasc Genet 9, 436-447.

35. Wilson, R., Wahl, S., Pfeiffer, L., Ward-Caviness, C.K., Kunze, S., Kretschmer, A., Reischl, E., Peters, A., Gieger, C., and Waldenberger, M. (2017). The dynamics of smoking-related disturbed methylation: a two time-point study of methylation change in smokers, non-smokers and former smokers. BMC Genomics 18, 805.

36. Dogan, M.V., Shields, B., Cutrona, C., Gao, L., Gibbons, F.X., Simons, R., Monick, M., Brody, G.H., Tan, K., Beach, S.R., *et al.* (2014). The effect of smoking on DNA methylation of peripheral blood mononuclear cells from African American women. BMC Genomics 15, 151.

37. Zeilinger, S., Kuhnel, B., Klopp, N., Baurecht, H., Kleinschmidt, A., Gieger, C., Weidinger, S., Lattka, E., Adamski, J., Peters, A., *et al.* (2013). Tobacco smoking leads to extensive genome-wide changes in DNA methylation. PLoS One 8, e63812.

38. Peters, M.J., Joehanes, R., Pilling, L.C., Schurmann, C., Conneely, K.N., Powell, J., Reinmaa, E., Sutphin, G.L., Zhernakova, A., Schramm, K., *et al.* (2015). The transcriptional landscape of age in human peripheral blood. Nat Commun 6, 8570.
