# Supplementary material for: Patterns of Reliability: Assessing the Reproducibility and Integrity of DNA Methylation Measurement
Source: Patterns (N Y). 2020 Apr 23;1(2):100014. doi: 10.1016/j.patter.2020.100014 (PMC7467214; doi:10.1016/j.patter.2020.100014)
Supplement: Document S2. Article plus Supplemental Information [file mmc3.pdf]

Patterns

Article

Patterns of Reliability: Assessing the Reproducibility and Integrity of DNA Methylation Measurement

Graphical Abstract

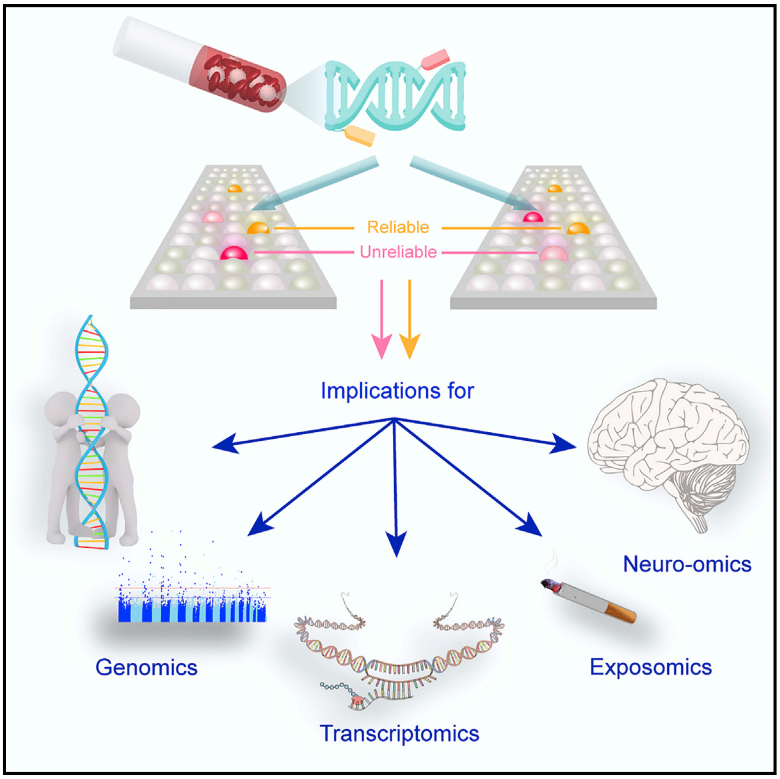

Highlights

- Measurements of DNA methylation made using BeadChip probes are differentially reliable
- Unreliable probes were less heritable, less replicable, and less functionally relevant
- This has serious implications for reporting and evaluating DNA methylation findings
- Reliability joins replicability and reproducibility to make three fundamental Rs of STEM

Authors

Karen Sugden, Eilis J. Hannon, Louise Arseneault, ..., Chloe C.Y. Wong, Jonathan Mill, Avshalom Caspi

Correspondence

karen.sugden@duke.edu

In Brief

DNA methylation is an important mechanism of gene regulation. The most popular method to measure methylation is to use BeadChips that contain probes to index hundreds of thousands of methylation sites at once. However, these probes are not equally reliable. In blood DNA, unreliable probes were less heritable and less likely to index gene expression, and associations were less replicable. This has serious downstream consequences for reproducible science and should serve as a caution for all data scientists regardless of discipline.

Sugden et al., 2020, Patterns *1*, 100014
May 8, 2020 © 2020 The Authors.
https://doi.org/10.1016/j.patter.2020.100014

CellPress

Patterns

CellPress
OPEN ACCESS

Article

Patterns of Reliability: Assessing the Reproducibility and Integrity of DNA Methylation Measurement

Karen Sugden,[1,2,9,*] Eilis J. Hannon,[3] Louise Arseneault,[4] Daniel W. Belsky,[5] David L. Corcoran,[2] Helen L. Fisher,[4] Renate M. Houts,[1] Radhika Kandaswamy,[4] Terrie E. Moffitt,[1,2,4,6] Richie Poulton,[7] Joseph A. Prinz,[2] Line J.H. Rasmussen,[1,8] Benjamin S. Williams,[1,2] Chloe C.Y. Wong,[4] Jonathan Mill,[3] and Avshalom Caspi[1,2,4,6]

[1]Department of Psychology and Neuroscience, Duke University, Grey Building, 2020 West Main Street, Suite 201, Durham, NC 27705, USA
[2]Center for Genomic and Computational Biology, Duke University, Durham, NC, USA
[3]Complex Disease Epigenetics Group, University of Exeter Medical School, Exeter, UK
[4]King's College London, Social, Genetic, and Developmental Psychiatry Research Centre, Institute of Psychiatry, Psychology, and Neuroscience, London, UK
[5]Department of Epidemiology & Butler Aging Center, Columbia University Mailman School of Public Health, New York, NY, USA
[6]Department of Psychiatry and Behavioral Sciences, Duke University School of Medicine, Durham, NC, USA
[7]Dunedin Multidisciplinary Health and Development Research Unit, University of Otago, Dunedin, New Zealand
[8]Clinical Research Centre, Copenhagen University Hospital Amager and Hvidovre, Hvidovre, Denmark
[9]Lead Contact
*Correspondence: karen.sugden@duke.edu
https://doi.org/10.1016/j.patter.2020.100014

THE BIGGER PICTURE Although DNA methylation data are used widely by researchers in many fields, the reliability of these data are surprisingly variable. Our findings remind us that, in an age of increasingly big data, research is only as robust as its foundations. We hope that our findings will improve the integrity of DNA methylation studies. We also hope that our findings serve as a cautionary reminder for those generating and implementing big data of any type: reliability is a fundamental aspect of replicability. Conducting analysis with reliable data will improve chances of replicable findings, which might lead to more actionable targets for further research. To the extent that reliable data improve replicability, the knock-on effect will be more public confidence in research and less effort spent trying to replicate findings that are bound to fail.

1 2 3 4 5 **Development/Pre-production:** Data science output has been rolled out/validated across multiple domains/problems

SUMMARY

DNA methylation plays an important role in both normal human development and risk of disease. The most utilized method of assessing DNA methylation uses BeadChips, generating an epigenome-wide "snapshot" of >450,000 observations (probe measurements) per assay. However, the reliability of each of these measurements is not equal, and little consideration is paid to consequences for research. We correlated repeat measurements of the same DNA samples using the Illumina HumanMethylation450K and the Infinium MethylationEPIC BeadChips in 350 blood DNA samples. Probes that were reliably measured were more heritable and showed consistent associations with environmental exposures, gene expression, and greater cross-tissue concordance. Unreliable probes were less replicable and generated an unknown volume of false negatives. This serves as a lesson for working with DNA methylation data, but the lessons are equally applicable to working with other data: as we advance toward generating increasingly greater volumes of data, failure to document reliability risks harming reproducibility.

INTRODUCTION

DNA methylation is an epigenetic mechanism that occurs by the addition of a methyl (CH_3) group to DNA, resulting in modification of genetic function without changes to DNA sequence. This mechanism plays an important role in human development and disease, primarily by regulating gene expression.[1] Because of the modifiable nature of epigenetic influence, research into

This is an open access article under the CC BY license (http://creativecommons.org/licenses/by/4.0/).

DNA methylation has heralded a new era in the elusive search for the route by which the external world might "get under the skin."[2] By its very nature, this question spans multiple disciplines; geneticists,[3] biologists,[4] computational scientists,[5] neuroscientists,[6] social scientists,[7] and philosophers[8] have been drawn to massive new data about the epigenome with an eye toward how it might explain health, disease, and our very nature. The promise of the epigenetics revolution has been sweeping.

In humans, DNA methylation occurs at specific sites across the genome (almost exclusively CpG sites, where a cytosine nucleotide is located next to a guanidine nucleotide), and there exist hundreds of thousands of such sites. Advances in technologies for quantifying site-specific DNA methylation have aided an explosion of research aimed at identifying associations between numerous environmental exposures, disease processes, and methylomic variation.[9–12] One such measurement technology, the Infinium BeadChip produced commercially by Illumina, has fueled much of the research in epigenetic epidemiology. This platform was developed to simultaneously assay thousands of DNA methylation targets in the genome. The relative ease of use, low cost, and modest sample requirements of this technology have enabled a new generation of researchers to add DNA methylation to their research programs, which only a few years ago would have posed an insurmountable challenge. We are among this new generation. This article reports our experience, excitement, and frustration, as a team of multidisciplinary scientists, trying to understand and use these data.

When we began to produce DNA methylation data, we reviewed the literature for best-practice information and guidelines to ensure the highest validity and downstream reproducibility. It was at this point we realized there was no consensus. We had generated data using the Infinium Methylation450 (450K) BeadChip, the gold standard for epigenome-wide DNA methylation data. This provides ~450,000 measurements per individual subject. However, we learned that a significant proportion of the thousands of data points do not yield the equivalent value when quantified twice from the same DNA sample.[13,14] This situation is compounded by the nature of our work, which involves repeated measurement of individuals studied longitudinally. This in itself raises an additional complication: measurement methods become obsolete and are superseded by new, improved products. In this case, the 450K BeadChip was recently replaced by the Infinium MethylationEPIC (EPIC) BeadChip, which contains most of the content (approximately 93%) of the 450K BeadChip augmented with probes covering an additional ~400,000 CpG sites. Published research has suggested that at the array level, DNA methylation values generated using both iterations of Illumina DNA methylation BeadChips are highly correlated, yielding correlations >0.9;[15–18] however, the reliability of individual-level probe measurements between the two arrays varies substantially. Using DNA derived from blood collected from 145 adults, one study[17] observed that reliability correlations between probes on the 450K and EPIC BeadChip ranged from −0.34 to 0.95 with a median value of 0.15, and only 2.6% of the ~420,000 probes assayed had reliability correlations above 0.8. Using DNA derived from blood collected from 109 newborns and 86 adolescents, a second study[18] observed similarly low correlations (median r = 0.23, only ~10% of probes with correlations >0.8).

These aforementioned reports documented *patterns* of uneven reliability in the repeated measurement of DNA methylation.[13,14,17,18] However, we were not prepared for the scarcity of information documenting the *consequences* of these patterns; consequences that, if shown to affect inferences made from DNA methylation data, would have widespread implications for reproducibility. Most research studies treat the ~450,000 observations as "equals," each as likely as the next to report true biological differences from a statistical point of view. However, to uncover consistent, replicable signals of DNA methylation dynamics, be it over time, between populations, or between exposures, measurement reliability is crucial. Analysis of probes that cannot be repeatedly measured with precision has the potential to yield irreproducible findings borne from spurious associations, and, just as importantly, may miss discoveries.

Here we share how we went about learning of the cross-disciplinary data challenges of high-throughput DNA methylation data and discuss the implications of these challenges for data processing, analysis, algorithm generation, and interpretation. Our goal is to promote communication about careful practices for working with the new data being generated in this important field.

We first performed test-retest measurement assessments to quantify the reliability of DNA methylation data. We assessed probe reliability between the two types of BeadChips using data on 350 DNA samples measured twice; once using the 450K BeadChip and again using the EPIC BeadChip. The individuals are participants in the E-Risk Study, a birth cohort of 2,232 twins born in 1994–1995 in the United Kingdom. DNA methylation was measured at age 18 years, when participants contributed whole blood for DNA analysis. Probe reliability was defined as the intraclass correlation (ICC) between repeat measures of individual probe β values measured on the two BeadChips. We then assessed the impact of differential reliability on numerous lines of enquiry of interest to many researchers, ourselves included. First, we tested how reliability influenced the ability to detect genetic and environmental effects on the epigenome through (1) analysis of heritability in the E-Risk twin sample and (2) analysis of methylation quantitative trait loci (mQTLs) identified in genome-wide association studies (GWAS) of DNA methylation. Second, we tested the implications of differential reliability for association testing by analyzing results of epigenome-wide association studies of tobacco smoking, one of the most harmful health risks in the modern world.[19] Third, we tested the implications of differential reliability for epigenetic biomarker development by analyzing multi-probe-algorithm-based measurements that are intended to capture information about aging (i.e., "DNA methylation clocks"). Finally, we tested the implications of differential reliability in ascribing biological function to DNA methylation by assessing the impact of reliability on (1) correlations between DNA methylation and gene expression and on (2) correlations between levels of DNA methylation measured in blood tissue and brain tissue.

RESULTS

Reliability of CpG Probes Is Low and Highly Variable

We use "reliability" to refer to the reproducibility of methylation probes' values. We measured probe values twice from the

same DNA source (DNA was sourced from a single blood draw via a single extraction). One set of measures was made using the 450K BeadChip, the other set using the EPIC BeadChip. Our analysis was restricted to probes found on both platforms (438,593 probes).

Probe reliabilities were computed using ICCs calculated for each of the 438,593 autosomal probes present on both the EPIC and 450K BeadChips that passed quality control. ICCs are an oft-used metric to assess reliability in test-retest situations,[20] and many different models exist depending on the way in which the test-retest data are generated. Here, we calculated ICCs based on a mean-rating ($k = 2$), absolute-agreement, 2-way random-effects model. We chose this model using the guidelines outlined by Koo and Li,[20] where mean-rating ($k = 2$) relates to the number of repeated measures (i.e., BeadChips per sample); absolute agreement requires that not only do the values across BeadChips correlate but that values are in agreement; and 2-way random effects relates to the generalizability of the ICCs to any subsequent similarly characterized rater (where rater = BeadChip probe).

ICCs between probes ranged from −0.28 to 1.00 (Supplemental Information, Section 1.1; Figure S1; Data S1). Probe reliabilities were skewed toward zero, with a mean of 0.21 (median = 0.09). This is low reliability considering that, in the context of establishing reliable measurement, ICCs below 0.4 are considered "poor," those between 0.4 and 0.6 are considered "fair," between 0.6 and 0.75 "good," and above 0.75 "excellent."[21]

The reliabilities that we observed in our data were highly correlated with the reliabilities observed by Logue et al.,[17] who also compared probes across 450K and EPIC BeadChips ($r = 0.86$, $p < 0.01$, Supplemental Information, Section 1.1; Figure S2). This suggests that the low reliabilities that we observed across the arrays are reproducible in other datasets. Importantly, the low reliabilities that we observed were unlikely to be solely due to differences between 450K and EPIC BeadChip probes. First, previous studies have documented similar low reliabilities in 450K-450K probe comparisons[13,14] and EPIC-EPIC probe comparisons.[17] Second, we conducted EPIC-EPIC array comparisons for a subset of Dunedin Study samples ($n = 28$) (for comparison purposes, we restricted analysis to the ~440,000 probes overlapping with the 450K array as described throughout this paper). Several noteworthy details emerged. (1) The median reliability in our EPIC-EPIC comparison was 0.26. This is higher than the median reliability (0.09) observed in our 450K-EPIC comparisons, but still falls squarely in what is considered to be "poor" reliability.[21] (2) It is not clear what accounts for the higher EPIC-EPIC reliability; it could be due to consistency of the platform or it could be due the fact that, unlike probes for the 450K-EPIC comparisons, probes for the EPIC-EPIC were assayed at the same time, using the same reagents, equipment, and so forth. (3) The correlations between the EPIC-EPIC reliabilities estimated by us in the Dunedin Study with the 450K-EPIC reliabilities (estimated by us in the E-Risk Study) was 0.77 (Figure S3). (4) When performing the analyses set forth in this manuscript using EPIC-EPIC ICCs rather than 450K-EPIC ICCs, we arrive at the same conclusions: we found that, like between-array reliability, within-array reliability is low, skewed toward zero, and has detrimental effects on research findings, and that differences in 450K and EPIC BeadChip probes are unlikely to be the sole cause of between-array unreliability (Supplemental Information, Section 1.1).

As a sanity check, we also sought to replicate previously observed associations between reliability and (1) the mean and standard deviation (SD) of methylation levels (β values)[13,14,17] (Supplemental Information, Section 1.2) and (2) the genomic annotation (location) of probes[13,18] (Supplemental Information, Section 1.3). We observed the same associations as previously reported. Taken together, this suggests BeadChip-wide differential reliabilities are reproducible and systematic in pattern.

Previous methodological studies have drawn attention to three factors that might compromise the quality of methylation BeadChip data: probe invariance,[22–24] potential probe hybridization problems,[25] and skewness.[26] We tested whether these features are sufficient to capture unreliability. They are not. Probe unreliability exists in probes that are variable or do not have potential probe hybridization problems, and probe reliabilities calculated on β values resemble the reliabilities of M values, a method for transforming skewed probe distributions[26] (Supplemental Information, Section 1.4).

In summary, we replicated previous reports of low reliability across probes common to the 450K and EPIC BeadChips, demonstrating that, paradoxically, poor reliability is reproducible. Moreover, factors commonly thought to account for unreliability (such as invariance) do not provide a satisfactory account of its ubiquity.

Evaluating the Consequences of Unreliable Probe Measurements

Our data suggest that the majority of probes we tested have low test-retest reliability. We now examine the practical implications of this observation for epigenetic research by applying our 450K-EPIC reliabilities to the results of previously published epigenetic studies. In all cases, these previously published studies were based on data derived using 450K BeadChips because (1) the EPIC BeadChip is relatively new, and most published research is based on the 450K BeadChip, (2) the probes common to the EPIC and 450K BeadChips reflect almost all (~93%[16]) of the probes unchanged from the 450K BeadChip, and (3) earlier 450K-450K comparisons showed patterns of reliabilities similar to those of the 450K-EPIC comparison.[13,14]

Estimates of Genetic and Environmental Effects on DNA Methylation Are Affected by Unreliable Measurement

Genetic and environmental effects on a phenotype can be estimated by comparing the relative phenotypic differences between monozygotic (MZ) and dizygotic (DZ) twins. The assumptions behind this model are that additive genetic factors are perfectly correlated between MZ twins (i.e., genetic correlation = 1) but are only 50% correlated between DZ twins (i.e., genetic correlation = 0.5) and that shared non-heritable influences are equally similar between MZ and DZ twin pairs. We previously reported the probe-specific genetic and environmental architecture of DNA methylation.[24] Using our twin design, we decomposed variation in each probe into three variance components: additive genetic effects (labeled "A"), shared environmental effects ("C"; environmental effects that each twin in a twin pair share, making twins more similar to each other), and non-shared (or unique) environmental effects ("E"; environmental effects that are specific to each twin within a pair, making twins less similar to

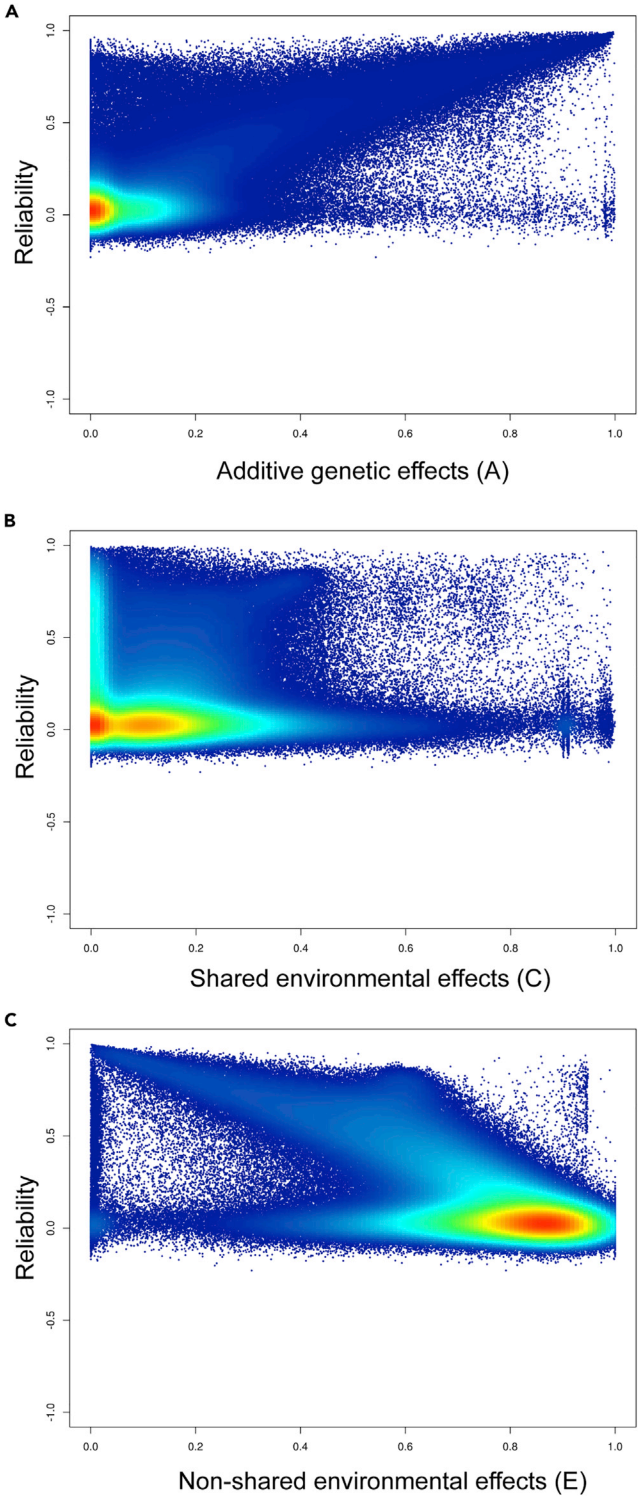

Figure 1. Density Heatmap of Probe Reliability Plotted against Estimates of Genetic and Environmental Effects on DNA Methylation
(A) Additive genetic effects (denoted as "A"), (B) shared environmental effects (denoted as "C"), and (C) non-shared (or unique) environmental effects (denoted as "E"). The variance component is plotted on the x axis and the reliability is plotted on the y axis. Probes with the highest reliability have the highest value of A and lowest value of E. Density is depicted on a spectral scale from low (dark blue) to high (red).

each other). Figure 1 shows the association between probe reliability and estimates of A (Figure 1A), C (Figure 1B), and E (Figure 1C). Reliability was significantly correlated with higher heritability ($r = 0.70$, $p < 0.01$, Figure 1A). In contrast, low-reliability probes tended to be suffused with more non-shared environmental variance ($r = -0.58$, $p = 1.00$, Figure 1C). Given that the non-shared environmental variance component in biometric models also includes measurement error, these probes are possibly less likely to reflect true environmental effects than they are to reflect unreliable measurement. (The correlation between reliability and estimates of shared environmental variance [C] was low, $r = -0.07$, possibly reflecting the fact that the classical twin design has limited power to identify precise estimates of shared environmental influence.[27])

We further examined how unreliability affects discovery research about the genetic etiology of DNA methylation. A recent GWAS of DNA methylation identified ~55,000 methylation mQTLs, DNA sequence variants that are associated with differential DNA methylation.[28] Figure 2 shows that the reliability of probes indexed by mQTLs in our data (N = 50,900) is higher than the reliability of probes that are not (N = 387,693).

In summary, given that a significant proportion of probes are suffused with unreliability (as indicated by poor test-retest reliability and as further indexed by high E-components in biometric models), the ability to detect associations between DNA methylation levels and genetic influences will be compromised.

Probe Reliability Affects Association Testing

We hypothesized that reliability is related to the likelihood that associations between environmental exposures and specific probes would replicate across independent studies. To test this, we focused on one of the most robust findings in epigenetic epidemiology: the effect of tobacco smoking on DNA methylation. We identified 22 studies that reported an epigenome-wide analysis of current versus never smoking using the 450K BeadChip platform[12,29–34] (Table S4). For each study, we obtained lists of probe IDs and direction of effect for probes that were significantly associated with current smoking (as determined by the study authors; total number of probes = 3,724; number of probes per study = 84–2,441). We then determined the extent to which individual probes replicated across the 22 studies by summing the number of times each probe was listed with consistent direction of effect (i.e., consistent cross-study increases or decreases in methylation in response to smoking). The number of individual replications across studies was associated with reliability ($r = 0.52$, $p < 0.001$, Figure 3). The mean number of replications for low-reliability probes (here defined as reliability <0.4) was 6.84 (median = 1, SD = 6.78, n = 1,630 probes), whereas the mean for high-reliability probes (reliability >0.75) was 13.1 (median = 15, SD = 5.11, n = 391 probes).

In summary, the likelihood of replicating associations between exposures and DNA methylation probes is significantly greater when studying reliable probes. Unreliable probes are likely to generate false positives and to mask true associations and are less likely to be reproducible.

Publicly Available DNA Methylation Aging Algorithms Contain Unreliable Measurements

There is enormous interest in developing and applying algorithms that use DNA methylation to index biological aging.[35] A critical component of the success of these "DNA methylation

CellPress
OPEN ACCESS

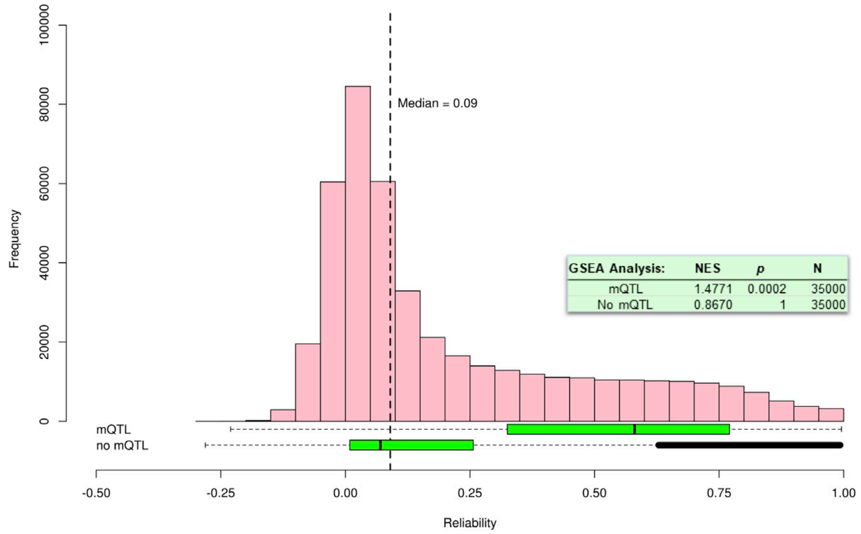

Figure 2. The Distribution of Reliabilities of Probes Identified in a Large-Scale mQTL Analysis Compared with Non-mQTL Probes

Distributions are depicted as box-and-whisker plots of the reliability coefficients of the probes identified as having mQTLs (“mQTL”) and the remainder not included in the mQTL list (“no mQTL”). Boxes correspond to interquartile range (IQR), and whiskers extend to 1.5 × IQR. Observations beyond the whiskers (outliers) are represented by individual points. As a reference, the distribution (pink bars) and median (vertical dashed line) of all ∼440,000 probe reliabilities in the E-Risk dataset is shown above the box-and-whisker plots. The text box shows the results of gene set enrichment analysis (GSEA; NES, normalized enrichment score; N, number of probes); probes associated with mQTLs are enriched for reliable probes, suggesting that reliable probe measurement is important for uncovering genetic effects on methylation.

clocks” is that probes comprising the algorithms are reliably measured so that they might be applied to any external dataset. We tested the hypothesis that these algorithms are more likely to capture reliable probes than unreliable probes. Figure 4 shows the distribution of probe reliabilities for three established DNA methylation aging-associated clocks: (1) the “Hannum clock,”[36] (number of probes = 63), (2) the “Horvath clock”[37] (number of probes = 334), and (3) the “Levine clock”[38] (number of probes = 512; the number of probes reflects those available in our data). Each aging algorithm had median probe reliabilities higher than that of the background distribution. However, the distribution for all three algorithms was not solely composed of reliable probes; each algorithm contained many probes whose β values were unreliable.

In summary, externally validated DNA methylation algorithms are generally composed of reliable probes. However, their performance could be improved by utilizing more reliable DNA methylation measurements. This perhaps emphasizes the point that algorithms of this type necessitate careful, extensive external validation; we hypothesize that algorithms over-represented by unreliable probes will, by their very nature, fail to perform well under varied testing situations.

Reliability Influences the Association between DNA Methylation and Gene Expression

A goal of epigenetic discovery is to assign biological meaning to the observed patterns of DNA methylation (e.g., Schubeler[2] and Teschendorff and Relton[39]). To this end, we tested the hypothesis that DNA methylation probes with higher reliability were more likely to index variation in gene expression, the process by which the information encoded in a gene is used to direct the assembly of a protein molecule. We used two approaches.

First, we used the results of global DNA methylation-gene expression correlation patterns described by Kennedy et al.,[40] wherein 36,485 and 114,536 unique DNA methylation probes were associated with gene expression across two cohorts (GTP and MESA, respectively; $p < 1 \times 10^{-5}$). Figure 5A shows that these significantly correlated methylation probes were more likely to be reliable (median reliability in GTP = 0.21, proportion of these probes with reliability >0.75 = 11.2%; median reliability in MESA = 0.20, proportion of these probes with reliability >0.75 = 10.1%; gene set enrichment analysis [GSEA] enrichment $p < 1 \times 10^{-4}$ in each) than methylation probes that were not discovered to be related to gene expression. Furthermore, probes that were significantly correlated with gene expression in both datasets had higher reliabilities than those identified in only one dataset (median reliability = 0.36 versus 0.17, proportion of probes with reliability >0.75 = 14.7% versus 9.4% for both datasets versus one dataset, respectively; GSEA enrichment $p < 1 \times 10^{-4}$). This suggests that reliability of DNA methylation probes influences the ability to detect correlates of biological function in a reproducible manner.

Second, using gene expression data available in the Dunedin Study, we calculated the correlation between gene expression probeset values with DNA methylation β values for every CpG probe localized to the transcription start site (TSS) of that gene. We restricted analysis to probes within the TSS, as these are hypothesized to have direct effects upon expression of the localized gene. As shown in Figure 5B, DNA methylation probes that significantly correlated with expression probesets ($\alpha = 1 \times 10^{-7}$, n = 278) had significantly higher reliabilities than DNA methylation probes that did not (n = 23,261; median reliability of correlated probes = 0.64, proportion of these probes with reliability >0.75 = 36.0%; median reliability of non-correlated probes = 0.04, proportion of these probes with reliability >0.75 = 3.4%; Figure 5C).

In summary, DNA methylation probes were more likely to correlate with transcriptional variation if they were reliably measured. Reliable probes are more likely to index reproducible biological correlates, whereas unreliable probes may mislead about biological function.

Reliability Influences the Concordance of Blood and Brain Methylation Levels

Most epidemiological investigations into exposure-related differential DNA methylation are undertaken using DNA derived from whole blood. This is an expedient choice due to the relative ease of collecting blood in population-based studies. However, many exposures in which epidemiologists are interested are hypothesized to have their effects (or consequences) in other

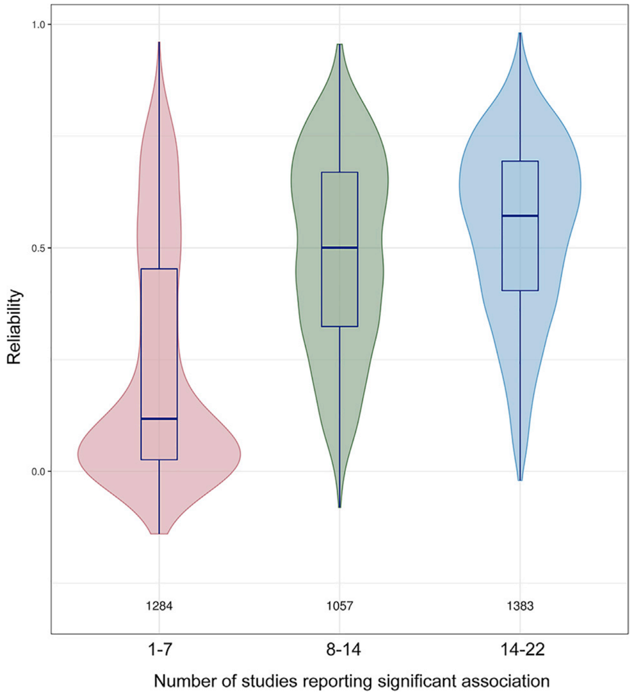

Figure 3. Probes Consistently Associated with Smoking across Studies Have Higher Reliabilities Than Probes that Are Not

We identified 22 epigenome-wide association studies of smoking and DNA methylation. For ease of visualization, probes have been binned into three groups representing 1–7 replications (pink), 8–14 replications (green), and 15–22 replications (blue). The values above the x axis represent the number of probes per group. In the 1–7 replication bin, the highest density of probes was at the low-reliability end of the distribution, and the median reliability (as depicted by the median line of the box plot within the violin) was the lowest of the three groups. Boxes correspond to IQR and whiskers extend to 1.5 × IQR.

tissues, such as the brain, raising the question of whether peripheral blood is a problematic surrogate tissue. Previously, we evaluated the similarity of methylation levels between blood DNA and DNA from four brain regions (prefrontal cortex, entorhinal cortex, superior temporal gyrus, and cerebellum) using the 450K BeadChip, and showed that only a small proportion of probes measured in blood correlate with methylation levels in the brain.[41]

We hypothesized that these small numbers of probes that register similar levels of DNA methylation in blood and brain tissue would be over-represented by high-reliability probes. To test this, we cross-referenced the correlations between DNA methylation levels in blood and each of four brain regions ("blood-brain concordance") with our 450K-EPIC probe reliabilities. Blood-brain concordance was related to reliability (rho = 0.22–0.38, $p < 0.01$ across the four brain regions). Figure 6 shows the distribution of reliability across low- (<0.4), mid- (0.4–0.75), and high-concordance (>0.75) probes in four brain regions. Median reliabilities for probes with low blood-brain concordance were 0.08 regardless of brain region, while median reliabilities for probes with high blood-brain concordance were 0.90 across the four brain regions. Moreover, probes that showed high blood-brain concordance in all four brain tissues were the most reliable (median reliability = 0.92, number of probes = 6,774, proportion of these probes with reliability >0.75 = 78.7%) while probes that had low blood-brain concordance in each of the four brain tissues were the least reliable (median reliability = 0.08, number of probes = 397,091, proportion of these probes with reliability >0.75 = 3.1%).

In summary, reliable probes are more likely to exhibit cross-tissue concordance in DNA methylation. Unreliable probes may be less likely to prove useful in developing blood-based biomarkers of brain dysregulation.

DISCUSSION

The reliability of probe-level DNA methylation measurement is highly variable across the ~440,000 sites indexed on the 450K and EPIC BeadChips. This differential reliability has detrimental downstream implications: it undermines published research and masks potential new discoveries.

First, we demonstrated that detection of both environmental and genetic effects on DNA methylation is related to differential probe reliability. The extent to which DNA methylation responds to environmental influences is under intense investigation and is thought to be one route via which environmental exposures "get under the skin."[2] There is also much interest in the relationship between DNA sequence variation and DNA methylation.[23,24] Here, we showed that the most reliable probes tend to be under significant genetic influence, whereas the least reliable probes are suffused with non-shared environmental variation (which also includes variation arising due to measurement error). These findings suggest that for a proportion of sites that indicate high sensitivity to environmental input, identification of true signal might be hindered by the relatively higher probability of imprecise measurement and that insights into the genetic basis of methylation may be missed due to the poor reliability of DNA methylation.

Second, we demonstrated the implications of differential reliability for epigenome-wide association testing. To achieve this we focused on tobacco smoking, one of the most replicable findings in epigenetic epidemiology. Here we showed that the likelihood of replication across studies increases with probe reliability. We also showed how unreliable probes may slow biomarker discovery. Arguably, "DNA methylation clocks" have been one of the major success stories of epigenetic epidemiology.[36–38] We found that these clocks are enriched for reliable probes but that the algorithms also contain noisy measurements, and it is possible that applying machine learning to uniformly reliable data will improve precision in this and other areas.

Third, we demonstrated the implications of differential reliability for integrating DNA methylation data with sequence and transcriptomic data. Here we showed that probe reliability is necessary to accurately estimate genetic contributions to DNA methylation, to identify gene expression correlates, and to detect correlated DNA methylation signatures across tissues. If the goal is robust and replicable biological inference from site-specific DNA methylation, it is necessary to restrict analysis to those probes that can be reliably measured.

There are some caveats to this study. First, these findings are restricted to DNA derived from blood. However, findings described here will be of use to the majority of researchers in

CellPress
OPEN ACCESS

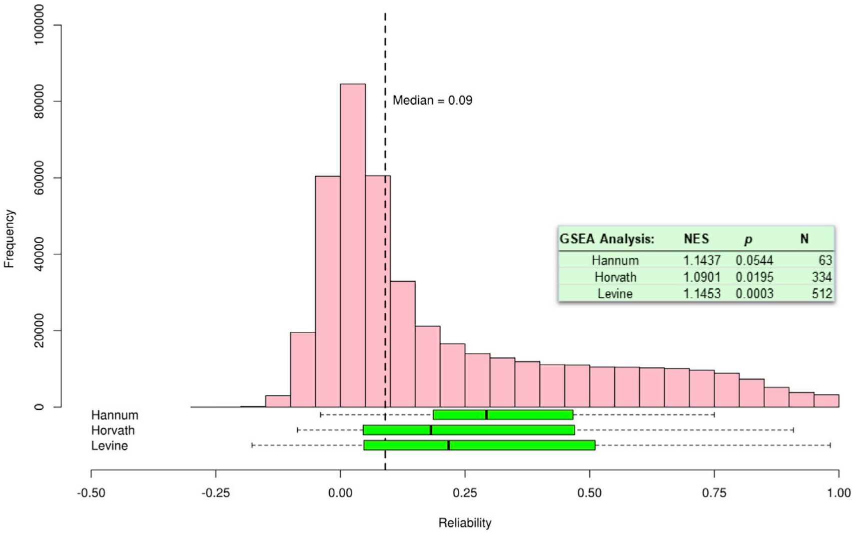

Figure 4. Reliabilities of Probes Included in Established, Publicly Available DNA Methylation Algorithms ("Clocks")

Distributions are depicted as box-and-whisker plots of the reliability coefficients of the probe constituents of the Hannum et al.[36] aging clock (63 probes), Horvath[37] DNAmAge clock (334 probes), and Levine et al.[38] biological aging clock (512 probes). Boxes correspond to IQR and whiskers extend to 1.5 × IQR. Observations beyond the whiskers (outliers) are represented by individual points. As a reference, the distribution (pink bars) and median (vertical dashed line) of all ~440,000 probe reliabilities in the E-Risk dataset is shown above the box-and-whisker plots. The aging clocks are enriched for reliable probes (values to the right of the figure; NES, normalized enrichment score; N, number of probes). Median reliabilities of probes included in aging clocks are higher than those of the general distribution; however, each algorithm contained many unreliable probes.

epigenetic epidemiology and to researchers looking for clinical application of epigenetic findings, since blood is the most common substrate from which DNA is derived and biomarkers are developed. Second, our study comprises young adults; it is possible that age-related change in DNA methylation at certain sites in the genome influences the pattern of reliability. That said, the pattern of reliability coefficients observed in our study is consistent with that seen in newborns,[18] 14-year-olds,[18] and ~30-year-olds.[17] Third, findings are restricted to the ~440,000 probes common to both the 450K and EPIC BeadChips. However, Logue et al.[17] reported similar reliability distributions for EPIC-EPIC comparisons in 11 individuals and we found better, but overall poor reliability for EPIC-EPIC comparisons in our data as well. Moreover, for the probes overlapping the two arrays, the EPIC-EPIC reliabilities were highly correlated with the 450K-EPIC reliabilities. The reason we emphasize between-array probe comparisons is that the goal of many researchers' work is to both make discoveries and replicate discoveries made by others. Given rapid advances in technologies and the proliferation of available data, it is increasingly the case that

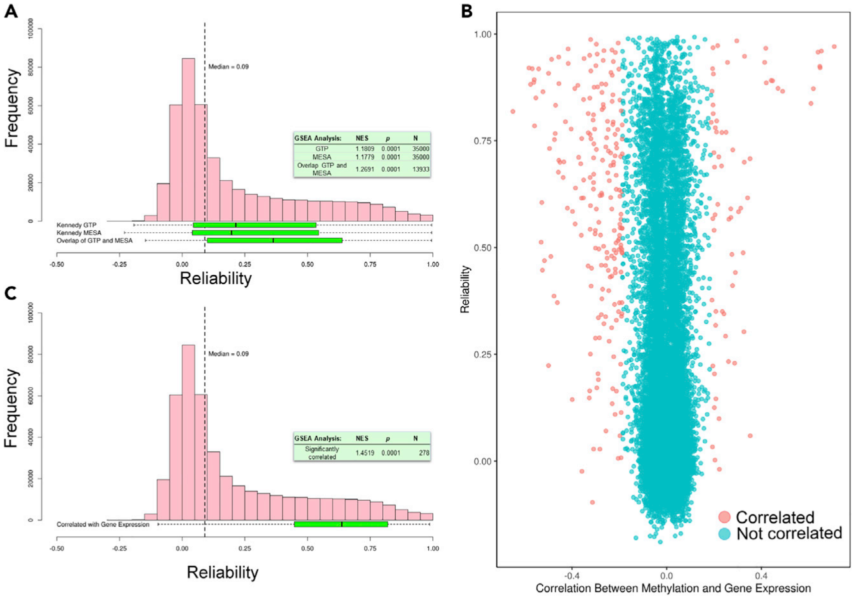

Figure 5. Reliabilities of Probes Significantly Correlated with Gene Expression Have Higher Reliabilities Than Non-correlated Probes

(A) Distributions of the reliability coefficients of the probes identified as correlated with gene expression by Kennedy et al.[40] in the GTP and MESA cohorts (N probes = 36,485 and 114,536, respectively). Probes that are correlated with gene expression in both cohorts are shown in the bottom-most box-and-whisker plot. Boxes correspond to IQR and whiskers extend to 1.5 × IQR. As a reference, the distribution (pink bars) and median (vertical dashed line) of all ~440,000 probe reliabilities in the E-Risk dataset is shown above the box-and-whisker plots. The text box shows the results of GSEA for the GTP cohort, MESA cohort, and the intersection of both cohorts (NES, normalized enrichment score; N, number of probes). Each cohort's set of significantly correlated DNA methylation probe-gene expression pairs is enriched for reliable probes; pairs that are significantly correlated in both datasets are further enriched.

(B) TSS-localized DNA methylation probe-gene expression probeset correlation (x axis) plotted against DNA methylation probe reliability (y axis) in the Dunedin Study dataset. Probes that were significantly correlated with gene expression are shown in pink (n = 278) and those not correlated are shown in blue.

(C) Distribution of reliabilities of these significantly correlated DNA methylation probes as a box-and-whisker plot. The text box shows the results of GSEA (NES, normalized enrichment score; N, number of probes); DNA methylation probes that were significantly correlated with expression probesets are enriched for reliable probes.

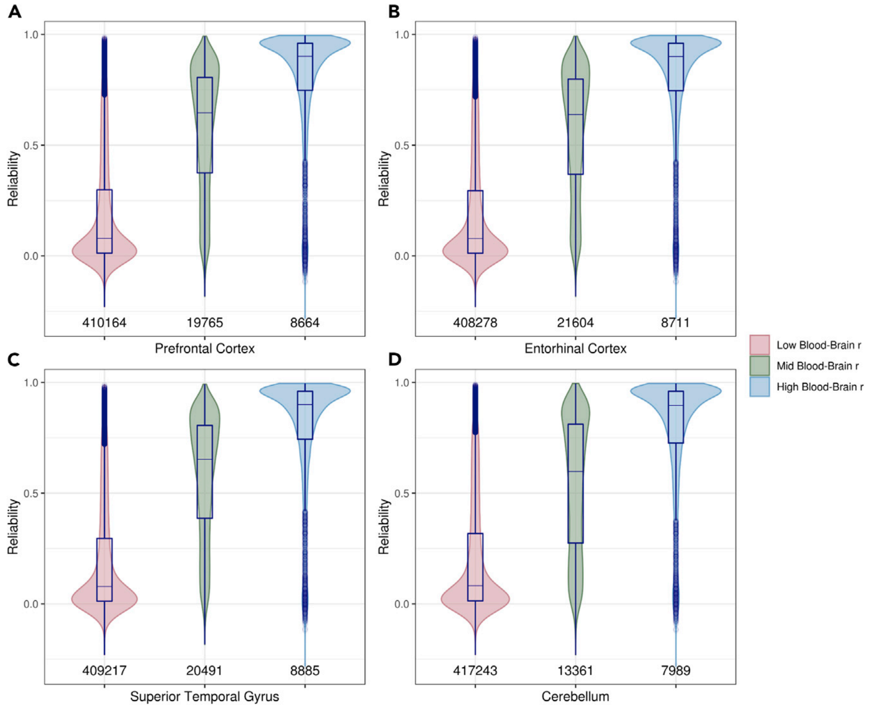

Figure 6. Violin Plots of the Distribution of Reliability in Probes with Low (<0.4, Pink), Medium (0.4–0.75, Green), and High (>0.75, Blue) Blood-Brain Correlation in DNA Methylation

Distributions are shown across four brain regions: prefrontal cortex (A), entorhinal cortex (B), superior temporal gyrus (C), and cerebellum (D). Number of probes per group is listed above the x axis. Box-and-whisker plots of the distribution are plotted within violin plots. Values below each violin correspond to the number of probes in that group. Probes with high blood-brain concordance are concentrated at the high-reliability end of the distribution. Boxes correspond to IQR and whiskers extend to 1.5 × IQR.

researchers need to integrate data that have been created using different arrays; indeed, although the 450K chip is no longer available, the vast bulk of DNA methylation research to date has used this array. As such, an important challenge for data scientists is how to integrate data from different arrays, whether this is in the service of evaluating targets for further scientific interrogation or in meta-analysis (e.g., one needs to know whether published results generated by 450K data are generalizable to new EPIC data and, ultimately, whether EPIC data will be generalizable to new technologies in future). In this case, between-array reliability is the relevant metric.

Taken together, at the very least unreliable probes are uninformative. At worst, they hinder scientific progress. In the GWAS world, much has been done to improve replicability of research, from increasing sample sizes to standardizing data pipelines (e.g., Visscher et al.[42]). In the epigenetic world, researchers have adopted many similar considerations (e.g., Lehne et al.[43] and Yan et al.[44]), but unreliability in the quantitative measurement of DNA methylation is a unique challenge. We list possible responses below.

First, to approximate reliable measurements, it is possible to filter data based on intrinsic properties of probes, such as invariance or hybridization properties. However, restricting analysis to variant probes or to probes without sequence-related performance issues is not sufficient to guarantee reliability; we found that these probes were not uniformly reliable (Supplemental Information, Section S1.4). Furthermore, restricting analysis to only variant probes conveys no enhancement of power to detect associations between reliability and (1) estimates of genetic and environmental influences on DNA methylation, (2) mQTL probes, and (3) concordance in DNA methylation levels between blood and brain tissue (Supplemental Information, Section S2). Second, it is possible to return to the practice, once routine, of using alternative technologies such as pyrosequencing to perform post-analysis validation of positive DNA methylation findings. This approach comes with two caveats, one of which is that it can only detect false positives; false negatives would remain undetected. A second caveat is that as science is shifting toward a culture of open-access and publicly available data, more and more researchers are becoming endpoint data users and as such are not involved in experimental data production. In this scenario, the task of experimental validation of individual findings, potentially in the thousands, is resource heavy, logistically impractical, and financially prohibitive. A third response is to generate pre-analysis reliability metrics, as we did in this report. Indeed, for publicly available data, this is currently the only feasible method of providing individual probe reliability metrics to end-users. To aid standardization, we have made available our reliability metrics for all measured probes (Data S1). Going forward, we suggest that researchers make the assessment of reliability standard practice when designing and measuring DNA methylation. This is because, despite evidence that our individual probe reliabilities correlate highly with those reliabilities reported previously,[17] we do not yet know the full extent to which demographic (e.g., age), measurement (e.g., batch), and source (e.g., tissue) factors may influence measurement reliability. Additionally, specific experimental designs (e.g., longitudinal designs and meta-analyses requiring incorporation of data from different sources, array types and batches, or cross-sectional single time-point designs) would determine which type of reliability metric to employ (e.g., within-array versus between-array); the reliability metrics reported here might not be the most suitable. By subsetting our repeated samples and calculating reliability, we determined that running just 25 replicates will identify 80% of the reliable probes (reliability >0.75) identified when using 350 replicates (Supplemental Information, Section S3.2). Fulfilling this recommendation would require additional investments during project planning along with commitment of support from funding agencies. The effort associated with incorporating reliability assessment into routine quality control, as we propose, is far outweighed by the benefits to science from improved replicability. The goal would be, at the

very least, to report the reliability associated with any probe for which conclusions are drawn; this will allow readers to make independent assessments of the confidence in the probe measurements. Even better would be to filter data, before analysis, on the basis of reliability metrics. Subsetting data in this way should help reduce false positives (by reducing the probability of spurious associations) and possibly false negatives. Although familywise error-rate corrections would not be greatly affected (e.g., within the data we present here, Bonferroni correction would reduce the testing threshold from $\alpha = 1.14 \times 10^{-7}$ for $\sim$440,000 probes to $\alpha = 1.77 \times 10^{-6}$ for $\sim$28,000 probes with reliability >0.75), false-discovery-rate corrections may be affected.

Researchers from diverse disciplines have been drawn by the promise of DNA methylation as a convenient vector by which the social environment might exert its effects on an organism's biology. They are also drawn to the relative simplicity of Illumina BeadChip data in both content and comprehensiveness. Anecdotally, we have encountered two reactions to the phenomenon of differential reliability. First, some researchers have expressed little surprise at its existence, coupled with a belief in the self-correcting power of the field to purge false negatives and positives over time. Our response to this is that better use of intellectual and financial resources might be made in analysis of data that are pre-validated, rather than cycling through replication attempts using unreliable measures that are bound to fail. Second, others have expressed shock and alarm that this phenomenon exists at all; these researchers are often new to the field and are not intimately familiar with the nuances of how data are produced or their biological meaning. Our response here is that DNA methylation data are not universally unusable—their suitability for analysis is contextual. Determination of reliability gives researchers confidence in the data they are using, be they new adopters, end-users, or seasoned experts.

Open-access availability of data is accelerating with encouragement from journal publishers and funding agencies. More and more researchers are using these big data; DNA methylation data are only one example of such. End-users rely on providers to verify the integrity of data, but just because data are massive in scale does not preclude the need for careful evaluation of their precision. The reproducibility crisis in science has drawn attention to two Rs: reproducibility (the extent to which consistent results are obtained when an experiment is repeated with the exact same inputs) and replicability (the extent to which consistent results are obtained when an experiment is repeated with the same design but with inputs from other sources). Here, we highlight a potential third "R," reliability. Reliability is a fundamental aspect of replicability. If desired inputs do not yield the same value when the source differs, replication is impossible. In this sense, test-retest reliability is a tool that has widespread applicability to the entire data-science community, especially where big data are used. The National Academies of Sciences, Engineering, and Medicine recently published a report[45] on the state of reproducibility and replicability in science, along with suggestions for improvement: "…[(r]esearchers should, as applicable to the specific study, provide an accurate and appropriate characterization of relevant uncertainties when they report or publish their research …". Unreliable probe measurement is one such uncertainty. We hope that our findings will improve the integrity of DNA methylation studies and serve as a cautionary reminder for those generating and implementing big data of any type.

EXPERIMENTAL PROCEDURES

Full details are provided in Supplemental Information, Section S3.

Samples

We report data from two samples. The Environmental Risk (E-Risk) Longitudinal Twin Study tracks the development of a 1994–1995 birth cohort of 2,232 British children followed to age 18 years.[46] The Dunedin Study tracks the development of a 1972–1973 birth cohort of 1,037 New Zealand children followed to age 45 years.[47]

DNA Methylation

In E-Risk, DNA was derived from peripheral blood drawn at age 18 years. In Dunedin, peripheral whole blood was drawn at 38 and 45 years. In E-Risk, DNA from 350 study members was selected for analysis using both Infinium MethylationEPIC (EPIC; Illumina, CA, USA) and Illumina Infinium HumanMethylation450 BeadChip (450K BeadChip; Illumina). The remainder of the cohort (n = 1,308) was assayed using the 450K BeadChip only, as previously described.[48] In Dunedin at age 38, DNA from 819 study members was assayed using the 450K BeadChip, as previously described.[48] In Dunedin at age 45, DNA from 28 study members was assayed twice using the EPIC BeadChip. E-Risk DNA methylation assays were run by the Complex Disease Epigenetics Group at the University of Exeter Medical School (UK) (www.epigenomicslab.com), and Dunedin assays were run by the Molecular Genomics Shared Resource at the Duke Molecular Physiology Institute, Duke University (USA).

Gene Expression

RNA was derived from peripheral blood drawn into PAXGene RNA tubes at age 38 years in Dunedin. Expression data were generated from RNA using the Affymetrix PrimeView Human Gene Chip (Affymetrix, CA, USA) by the Duke University Microarray Core Facility. Data quality control and RMA (robust multichip average) normalization were carried out using the *affy* Bioconductor package in the R statistical programming environment. After quality control, expression data were available for 836 individuals.

Probe Reliabilities

Probe reliabilities are computed using intraclass correlation (ICC) estimates, calculated for each autosomal probe present on both the EPIC and 450K BeadChip (N = 438,593). ICCs are an oft-used metric to assess reliability in test-retest situations,[20] and many different models exist depending on the way in which the test-retest data are generated. Here, we calculated ICCs based on a mean-rating ($k = 2$), absolute-agreement, 2-way random-effects model. To compare whether test-retest model choice had an effect on reliability estimates, we also computed Pearson product-moment correlation coefficients. Pearson correlation coefficients and ICC estimates of reliability were highly similar ($r = 1.00$, $p < 1 \times 10^{-4}$; Figure S9).

Statistical Analysis

All analyses were performed in the R statistical programming environment, often using Bioconductor packages. Unless otherwise noted, correlations are reported as Pearson correlation coefficients. Summary statistics, such as probe mean and SD, were based on the 350 samples processed on the 450K array. GSEA was performed using the *fgsea* Bioconductor package[49] with 10,000 permutations. The proportion of variance in DNA methylation explained by heritable (A), shared environmental (C), and unshared or unique environmental (E) factors was estimated using structural equation modeling implemented with functions from the *OpenMx* R package.[50]

DATA AND CODE AVAILABILITY

E-Risk 450K DNA methylation data are accessible from the Gene Expression Omnibus (accession code GEO: GSE105018). Data from the Dunedin Study are not publicly available due to lack of informed consent and ethical approval

for open access, but are available on request by qualified scientists. Requests require a concept paper describing the purpose of data access, ethical approval at the applicant's institution, and provision for secure data access. We offer secure access on the Duke University, Otago University, and King's College London campuses. All data on probe reliability and characteristics for the 450K-EPIC comparison (Data S1) are available at https://osf.io/83ucs/. The data underlying analysis of consequences of unreliability on heritability and blood-brain concordance are available from https://www.epigenomicslab.com/online-data-resources/. Code is available on request from the corresponding author.

SUPPLEMENTAL INFORMATION

Supplemental Information can be found online at https://doi.org/10.1016/j.patter.2020.100014.

ACKNOWLEDGMENTS

We thank the E-Risk Study and Dunedin Study members, research staff, and Dunedin Study founder Phil Silva. The E-Risk Study is funded by the Medical Research Council (G1002190 to T.E.M., L.A., and A.C.), the National Institute of Child Health and Human Development (HD077482 to A.C.), a joint UK Economic and Social Research Council (ESRC) and Biotechnology and Biological Sciences Research Council (BBSRC) grant (ES/N000277/1 to C.C.Y.W.), and an MQ Fellows Award (MQ14F40 to H.L.F.). The Dunedin Longitudinal Study is funded by the New Zealand Health Research Council, the New Zealand Ministry of Business, Innovation, and Employment (to R.P.), the National Institute on Aging (AG032282 to T.E.M. and A.C. and AG049789 to T.E.M.), and the Medical Research Council (MR/P005918/1 to T.E.M.). Additional support was provided by the Jacobs Foundation (to T.E.M. and A.C.), Charles Lafitte Foundation Duke Faculty Seed Grant (to A.C.), and by a Distinguished Investigator Award from the American Asthma Foundation to J.M. L.A. is the Mental Health Leadership Fellow for the UK Economic and Social Research Council, D.W.B. is a Jacobs Foundation Fellow, and H.L.F. is supported by a British Academy Mid-Career Fellowship (MD\170005). This work used a high-performance computing facility partially supported by grant 2016-IDG-1013 ("HARDAC+: Reproducible HPC for Next-generation Genomics") from the North Carolina Biotechnology Center. We would like to acknowledge the assistance of the Duke Molecular Physiology Institute Molecular Genomics core for the generation of data for this paper.

AUTHOR CONTRIBUTIONS

K.S., D.L.C., and A.C. devised the project and the main conceptual ideas. K.S., E.J.H., and D.L.C. processed and generated datasets. K.S. and D.L.C. performed the analyses and designed the figures. L.A., H.L.F., T.E.M., R.P., C.C.Y.W., J.M. and A.C. acquired funding and supervised project activities. K.S. and A.C. drafted the manuscript. All authors provided critical feedback and helped shape the research, analysis, and manuscript.

DECLARATION OF INTERESTS

The authors declare no competing interests.

Received: November 8, 2019
Revised: January 29, 2020
Accepted: February 27, 2020
Published: April 23, 2020

REFERENCES

1. Robertson, K.D. (2005). DNA methylation and human disease. Nat. Rev. Genet. *6*, 597–610.
2. Schubeler, D. (2015). Function and information content of DNA methylation. Nature *517*, 321–326.
3. Velasco, G., and Francastel, C. (2019). Genetics meets DNA methylation in rare diseases. Clin. Genet. *95*, 210–220.
4. Burggren, W.W., and Crews, D. (2014). Epigenetics in comparative biology: why we should pay attention. Integr. Comp. Biol. *54*, 7–20.
5. Ruskin, H.J., and Barat, A. (2018). Recent advances in computational epigenetics. Adv. Genomics Genet. *8*, 12.
6. Tognini, P., Napoli, D., and Pizzorusso, T. (2015). Dynamic DNA methylation in the brain: a new epigenetic mark for experience-dependent plasticity. Front. Cell. Neurosci. *9*, 331.
7. Chung, E., Cromby, J., Papadopoulos, D., and Tufarelli, C. (2017). Social epigenetics: a science of social science? Socio. Rev. *64*, 168–185.
8. Van Speybroeck, L. (2002). Philosophers and biologists exploring epigenetics. Biol. Philos. *17*, 743–746.
9. Klutstein, M., Nejman, D., Greenfield, R., and Cedar, H. (2016). DNA methylation in cancer and aging. Cancer Res. *76*, 3446–3450.
10. Verma, M., Rogers, S., Divi, R.L., Schully, S.D., Nelson, S., Joseph Su, L., Ross, S.A., Pilch, S., Winn, D.M., and Khoury, M.J. (2014). Epigenetic research in cancer epidemiology: trends, opportunities, and challenges. Cancer Epidemiol. Biomarkers Prev. *23*, 223–233.
11. Jones, M.J., Goodman, S.J., and Kobor, M.S. (2015). DNA methylation and healthy human aging. Aging Cell *14*, 924–932.
12. Joehanes, R., Just, A.C., Marioni, R.E., Pilling, L.C., Reynolds, L.M., Mandaviya, P.R., Guan, W., Xu, T., Elks, C.E., Aslibekyan, S., et al. (2016). Epigenetic signatures of cigarette smoking. Circ. Cardiovasc. Genet. *9*, 436–447.
13. Bose, M., Wu, C., Pankow, J.S., Demerath, E.W., Bressler, J., Fornage, M., Grove, M.L., Mosley, T.H., Hicks, C., North, K., et al. (2014). Evaluation of microarray-based DNA methylation measurement using technical replicates: the Atherosclerosis Risk in Communities (ARIC) Study. BMC Bioinformatics *15*, 312.
14. Dugue, P.A., English, D.R., MacInnis, R.J., Jung, C.H., Bassett, J.K., FitzGerald, L.M., Wong, E.M., Joo, J.E., Hopper, J.L., Southey, M.C., et al. (2016). Reliability of DNA methylation measures from dried blood spots and mononuclear cells using the HumanMethylation450k BeadArray. Sci. Rep. *6*, 30317.
15. Moran, S., Arribas, C., and Esteller, M. (2016). Validation of a DNA methylation microarray for 850,000 CpG sites of the human genome enriched in enhancer sequences. Epigenomics *8*, 389–399.
16. Pidsley, R., Zotenko, E., Peters, T.J., Lawrence, M.G., Risbridger, G.P., Molloy, P., Van Djik, S., Muhlhausler, B., Stirzaker, C., and Clark, S.J. (2016). Critical evaluation of the Illumina MethylationEPIC BeadChip microarray for whole-genome DNA methylation profiling. Genome Biol. *17*, 208.
17. Logue, M.W., Smith, A.K., Wolf, E.J., Maniates, H., Stone, A., Schichman, S.A., McGlinchey, R.E., Milberg, W., and Miller, M.W. (2017). The correlation of methylation levels measured using Illumina 450K and EPIC BeadChips in blood samples. Epigenomics *9*, 1363–1371.
18. Solomon, O., MacIsaac, J., Quach, H., Tindula, G., Kobor, M.S., Huen, K., Meaney, M.J., Eskenazi, B., Barcellos, L.F., and Holland, N. (2018). Comparison of DNA methylation measured by Illumina 450K and EPIC BeadChips in blood of newborns and 14-year-old children. Epigenetics *13*, 655–664.
19. Ioannidis, J.P.A. (2019). Neglecting major health problems and broadcasting minor, uncertain issues in lifestyle science. JAMA. https://doi.org/10.1001/jama.2019.17576.
20. Koo, T.K., and Li, M.Y. (2016). A guideline of selecting and reporting Intraclass correlation coefficients for reliability research. J. Chiropr. Med. *15*, 155–163.
21. Cicchetti, D.V., and Sparrow, S.A. (1981). Developing criteria for establishing interrater reliability of specific items: applications to assessment of adaptive behavior. Am. J. Ment. Defic. *86*, 127–137.
22. Edgar, R.D., Jones, M.J., Robinson, W.P., and Kobor, M.S. (2017). An empirically driven data reduction method on the human 450K methylation array to remove tissue specific non-variable CpGs. Clin. Epigenetics *9*, 11.
23. van Dongen, J., Ehli, E.A., Slieker, R.C., Bartels, M., Weber, Z.M., Davies, G.E., Slagboom, P.E., Heijmans, B.T., and Boomsma, D.I. (2014).

CellPress
OPEN ACCESS

Epigenetic variation in monozygotic twins: a genome-wide analysis of DNA methylation in buccal cells. Genes (Basel) *5*, 347–365.

24. Hannon, E., Knox, O., Sugden, K., Burrage, J., Wong, C.C.Y., Belsky, D.W., Corcoran, D.L., Arseneault, L., Moffitt, T.E., Caspi, A., et al. (2018). Characterizing genetic and environmental influences on variable DNA methylation using monozygotic and dizygotic twins. PLoS Genet. *14*, e1007544.
25. Naeem, H., Wong, N.C., Chatterton, Z., Hong, M.K., Pedersen, J.S., Corcoran, N.M., Hovens, C.M., and Macintyre, G. (2014). Reducing the risk of false discovery enabling identification of biologically significant genome-wide methylation status using the HumanMethylation450 array. BMC Genomics *15*, 51.
26. Du, P., Zhang, X., Huang, C.C., Jafari, N., Kibbe, W.A., Hou, L., and Lin, S.M. (2010). Comparison of Beta-value and M-value methods for quantifying methylation levels by microarray analysis. BMC Bioinformatics *11*, 587.
27. Burt, S.A. (2009). Rethinking environmental contributions to child and adolescent psychopathology: a meta-analysis of shared environmental influences. Psychol. Bull. *135*, 608–637.
28. McRae, A.F., Marioni, R.E., Shah, S., Yang, J., Powell, J.E., Harris, S.E., Gibson, J., Henders, A.K., Bowdler, L., Painter, J.N., et al. (2018). Identification of 55,000 replicated DNA methylation QTL. Sci. Rep. *8*, 17605.
29. Zeilinger, S., Kuhnel, B., Klopp, N., Baurecht, H., Kleinschmidt, A., Gieger, C., Weidinger, S., Lattka, E., Adamski, J., Peters, A., et al. (2013). Tobacco smoking leads to extensive genome-wide changes in DNA methylation. PLoS One *8*, e63812.
30. Besingi, W., and Johansson, A. (2014). Smoke-related DNA methylation changes in the etiology of human disease. Hum. Mol. Genet. *23*, 2290–2297.
31. Guida, F., Sandanger, T.M., Castagne, R., Campanella, G., Polidoro, S., Palli, D., Krogh, V., Tumino, R., Sacerdote, C., Panico, S., et al. (2015). Dynamics of smoking-induced genome-wide methylation changes with time since smoking cessation. Hum. Mol. Genet. *24*, 2349–2359.
32. Wilson, R., Wahl, S., Pfeiffer, L., Ward-Caviness, C.K., Kunze, S., Kretschmer, A., Reischl, E., Peters, A., Gieger, C., and Waldenberger, M. (2017). The dynamics of smoking-related disturbed methylation: a two time-point study of methylation change in smokers, non-smokers and former smokers. BMC Genomics *18*, 805.
33. Dogan, M.V., Shields, B., Cutrona, C., Gao, L., Gibbons, F.X., Simons, R., Monick, M., Brody, G.H., Tan, K., Beach, S.R., et al. (2014). The effect of smoking on DNA methylation of peripheral blood mononuclear cells from African American women. BMC Genomics *15*, 151.
34. Dogan, M.V., Beach, S.R.H., and Philibert, R.A. (2017). Genetically contextual effects of smoking on genome wide DNA methylation. Am. J. Med. Genet. B Neuropsychiatr. Genet. *174*, 595–607.
35. Horvath, S., and Raj, K. (2018). DNA methylation-based biomarkers and the epigenetic clock theory of ageing. Nat. Rev. Genet. *19*, 371–384.
36. Hannum, G., Guinney, J., Zhao, L., Zhang, L., Hughes, G., Sadda, S., Klotzle, B., Bibikova, M., Fan, J.B., Gao, Y., et al. (2013). Genome-wide methylation profiles reveal quantitative views of human aging rates. Mol. Cell *49*, 359–367.
37. Horvath, S. (2013). DNA methylation age of human tissues and cell types. Genome Biol. *14*, R115.
38. Levine, M.E., Lu, A.T., Quach, A., Chen, B.H., Assimes, T.L., Bandinelli, S., Hou, L., Baccarelli, A.A., Stewart, J.D., Li, Y., et al. (2018). An epigenetic biomarker of aging for lifespan and healthspan. Aging (Albany NY) *10*, 573–591.
39. Teschendorff, A.E., and Relton, C.L. (2018). Statistical and integrative system-level analysis of DNA methylation data. Nat. Rev. Genet. *19*, 129–147.
40. Kennedy, E.M., Goehring, G.N., Nichols, M.H., Robins, C., Mehta, D., Klengel, T., Eskin, E., Smith, A.K., and Conneely, K.N. (2018). An integrated -omics analysis of the epigenetic landscape of gene expression in human blood cells. BMC Genomics *19*, 476.
41. Hannon, E., Lunnon, K., Schalkwyk, L., and Mill, J. (2015). Interindividual methylomic variation across blood, cortex, and cerebellum: implications for epigenetic studies of neurological and neuropsychiatric phenotypes. Epigenetics *10*, 1024–1032.
42. Visscher, P.M., Wray, N.R., Zhang, Q., Sklar, P., McCarthy, M.I., Brown, M.A., and Yang, J. (2017). 10 Years of GWAS discovery: biology, function, and translation. Am. J. Hum. Genet. *101*, 5–22.
43. Lehne, B., Drong, A.W., Loh, M., Zhang, W., Scott, W.R., Tan, S.T., Afzal, U., Scott, J., Jarvelin, M.R., Elliott, P., et al. (2015). A coherent approach for analysis of the Illumina HumanMethylation450 BeadChip improves data quality and performance in epigenome-wide association studies. Genome Biol. *16*, 37.
44. Yan, H., Tian, S., Slager, S.L., Sun, Z., and Ordog, T. (2016). Genome-wide epigenetic studies in human disease: a primer on -omic technologies. Am. J. Epidemiol. *183*, 96–109.
45. The National Academies of Sciences, Engineering, and Medicine (2019). Reproducibility and Replicability in Science (National Academies Press).
46. Moffitt, T.E.; E-Risk Study Team (2002). Teen-aged mothers in contemporary Britain. J. Child Psychol. Psychiatry *43*, 727–742.
47. Poulton, R., Moffitt, T.E., and Silva, P.A. (2015). The Dunedin multidisciplinary health and development study: overview of the first 40 years, with an eye to the future. Soc. Psychiatry Psychiatr. Epidemiol. *50*, 679–693.
48. Marzi, S.J., Sugden, K., Arseneault, L., Belsky, D.W., Burrage, J., Corcoran, D.L., Danese, A., Fisher, H.L., Hannon, E., Moffitt, T.E., et al. (2018). Analysis of DNA methylation in young people: limited evidence for an association between victimization stress and epigenetic variation in blood. Am. J. Psychiatry *175*, 517–529.
49. Sergushichev, A.A. (2016). An algorithm for fast preranked gene set enrichment analysis using cumulative statistic calculation. bioRxiv. https://doi.org/10.1101/060012.
50. Neale, M.C., Hunter, M.D., Pritikin, J.N., Zahery, M., Brick, T.R., Kirkpatrick, R.M., Estabrook, R., Bates, T.C., Maes, H.H., and Boker, S.M. (2016). OpenMx 2.0: extended structural equation and statistical modeling. Psychometrika *81*, 535–549.

PATTER, Volume *1*

Supplemental Information

Patterns of Reliability:

Assessing the Reproducibility and Integrity

of DNA Methylation Measurement

Karen Sugden, Eilis J. Hannon, Louise Arseneault, Daniel W. Belsky, David L. Corcoran, Helen L. Fisher, Renate M. Houts, Radhika Kandaswamy, Terrie E. Moffitt, Richie Poulton, Joseph A. Prinz, Line J.H. Rasmussen, Benjamin S. Williams, Chloe C.Y. Wong, Jonathan Mill, and Avshalom Caspi

SUPPLEMENTAL EXPERIMENTAL PROCEDURES

Section S1: Describing the landscape of CpG probe reliability

This section relates to Main Text Results section entitled
'Reliability of CpG probes is low and highly variable'.

1.1 Reliability of CpG probes is low and highly variable. We began by assessing the distribution of probe-probe Intraclass Correlations (ICCs, henceforth 'reliability') across the 438,593 probes present on both the 450K and EPIC BeadChips in our data. Probe ICCs ranged from -0.28 to 1.00 (**Data S1,** https://osf.io/83ucs/). As shown in **Figure S1**, probe reliabilities were skewed towards zero, with a mean of 0.21 (median = 0.09). This is low reliability considering that, in the context of establishing reliable measurement, ICCs below .4 are considered "poor," those between .4 to .6 are considered "fair", between .6 to .75 "good", and above .75 "excellent".[1]

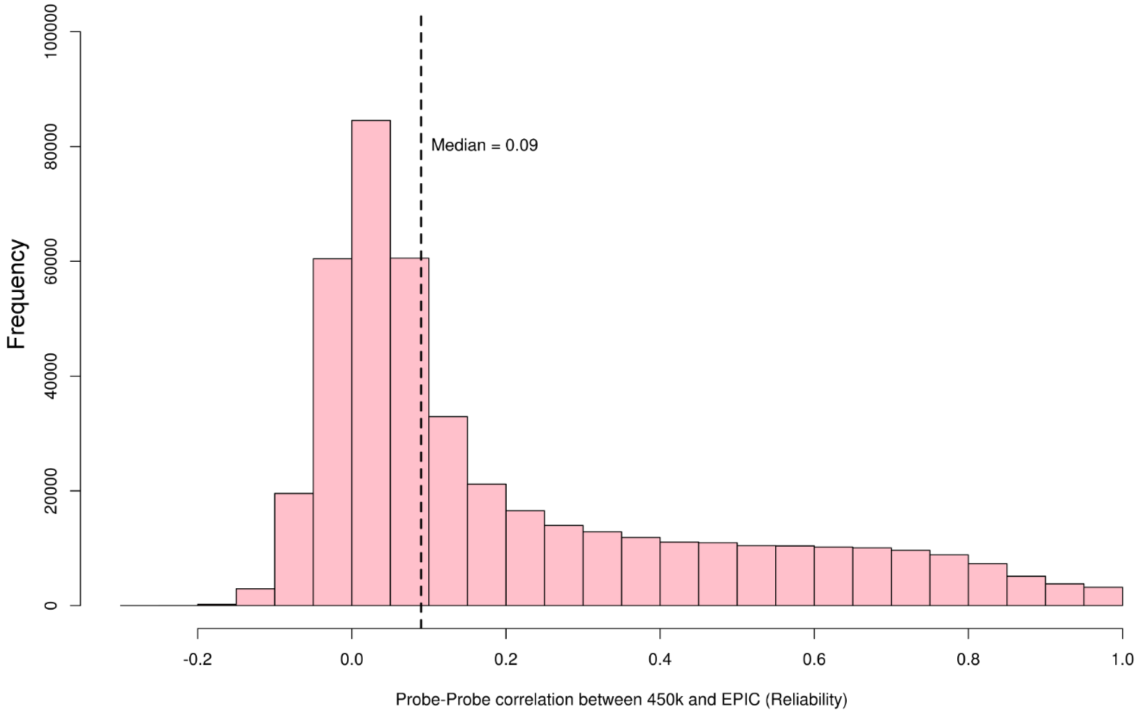

Figure S1: Distribution of reliability correlations for probes common to the 450K and EPIC BeadChips.

Low reliability might arise through experimental factors not related solely to poor probe performance. We therefore tested whether the pattern of reliabilities we observed might be due to such stochastic processes by comparing our reliabilities against those reported by Logue *et al.*[2], who also compared reliabilities of probes across 450K and EPIC BeadChips. The reliabilities were highly correlated ($r = 0.86$, $p < 0.01$, **Figure S2**), suggesting the reliabilities are reproducible and systematic in pattern.

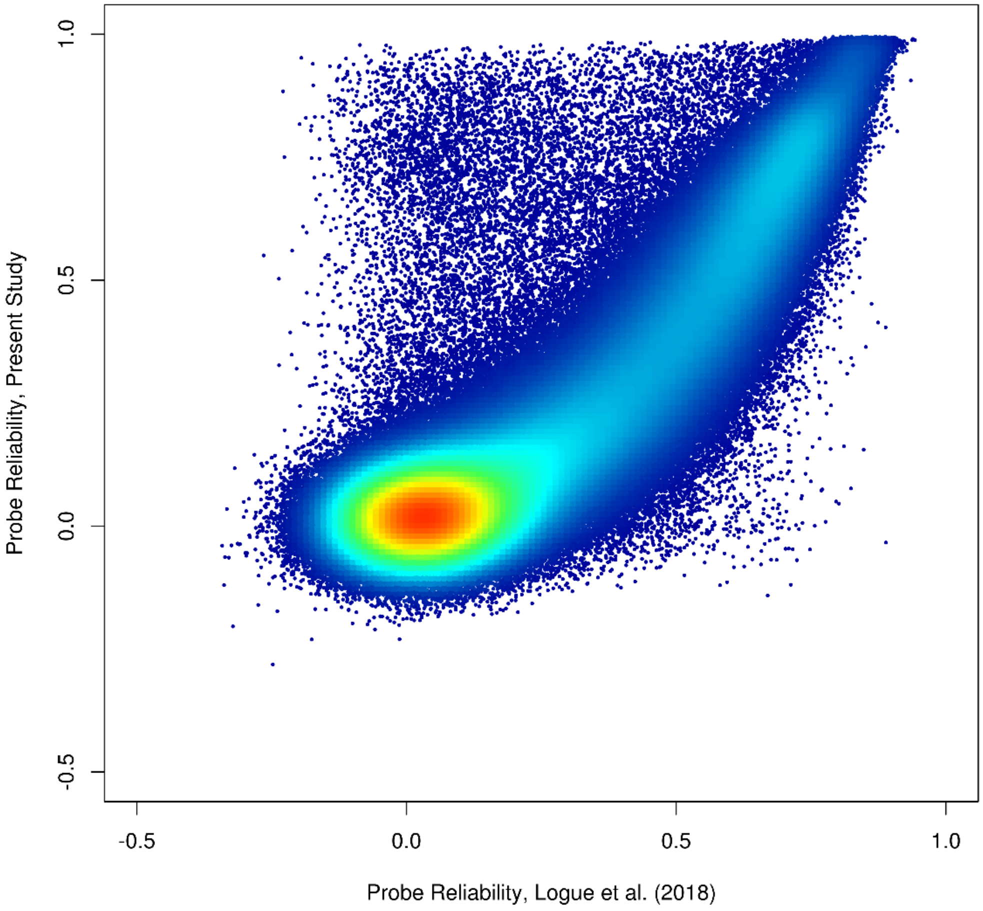

Figure S2. Differential probe reliabilities were consistent across studies. The y-axis plots probe reliabilities (as ICCs) in the present study, and the x-axis plots the reliabilities (as ICCs) reported by Logue *et al.*. Reliabilities were highly correlated ($r = 0.86$). Reliabilities were derived from comparisons between 450K and EPIC BeadChip.

An additional source of low reliability could be due to between-array (i.e. 450K vs EPIC) differences in probe performance. While this is unlikely since previous studies have documented low reliabilities in 450K-450K probe comparisons[3,4] and EPIC-EPIC probe comparisons[2], we nonetheless sought to independently determine whether within-array reliability followed similar patterns to between-array reliability. For this, we created a new reliability dataset comprised EPIC-EPIC (i.e. within-array) comparisons for a subset of Dunedin ($N = 28$) study samples (for comparison purposes, we restricted analysis to the ~440,000 probes overlapping with the 450K array as described throughout this manuscript). We sought to test if the distribution of reliabilities was similar between these two datasets.

We found that, like the between-array comparison, reliabilities for the within-array comparison were low and skewed towards zero (median = 0.26), and the two sets of reliabilities were significantly correlated with one another (r = 0.77, **Figure S3**). This suggests that differences between 450K and EPIC BeadChips are unlikely to be the sole cause of low probe reliability.

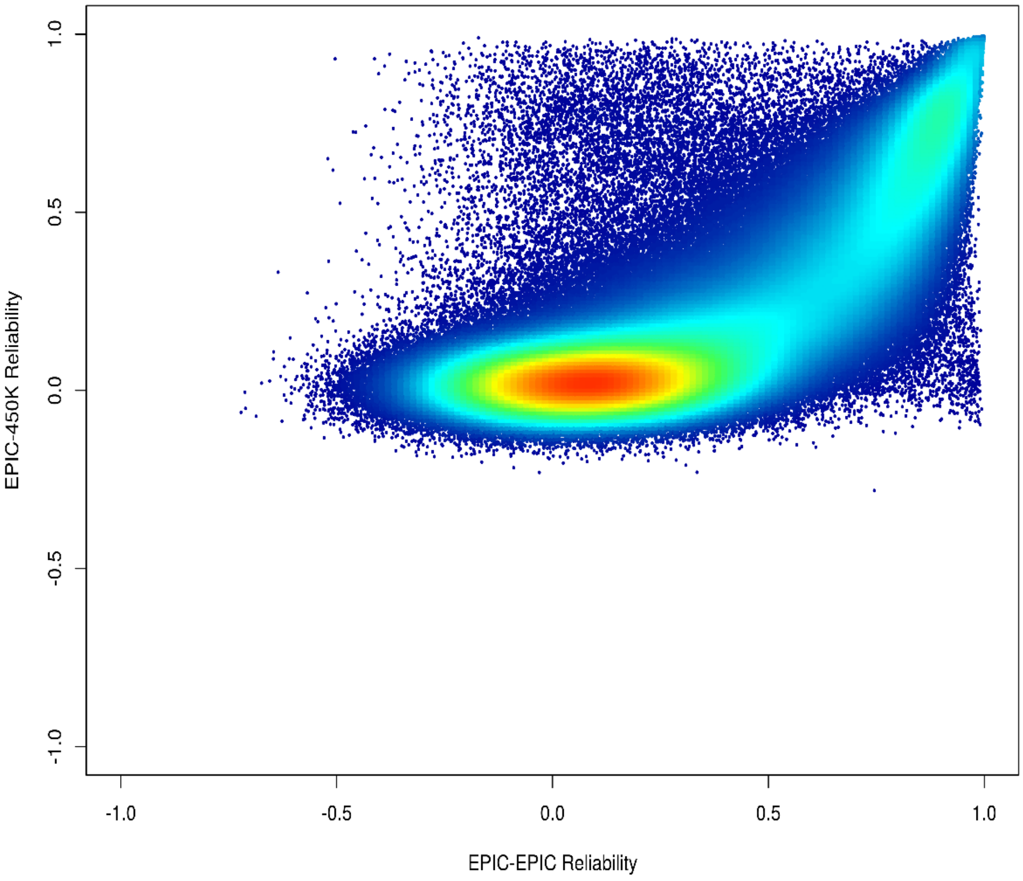

Figure S3. Between-array and within-array reliabilities are correlated. The y-axis plots the 450K-EPIC probe reliabilities used in the present study, and the x-axis plots EPIC-EPIC probe reliabilities from a subset of 28 individuals in the Dunedin Study. Reliabilities were highly correlated (r = 0.77), suggesting that unreliable probe measurement is systematic.

1.2 Probe-specific characteristics are related to reliability. Next, we tested if probe reliability was related to the mean and variance of methylation levels (β-values) at the site measured by the probe. Our analysis revealed three findings. First, probe-reliability had an inverse-U shaped relationship with mean β-values; the lowest-reliability probes were concentrated at either end of the distribution of methylation β-values (i.e. among hyper- and hypo-methylated probes), whereas the highest reliability probes were concentrated in the intermediate range of the distribution (**Figure S4A**). Second, the highest density of low reliability probes was found among probes with low β-value SD (**Figure S4B**). Third, β-value means and SDs were correlated (r = 0.15, P<0.01), and the most reliable probes were those with intermediate levels of methylation that varied most between individuals (**Figure S4C**). These observations confirm earlier reports of differential reliability as a function of site-specific characteristics[2-4].

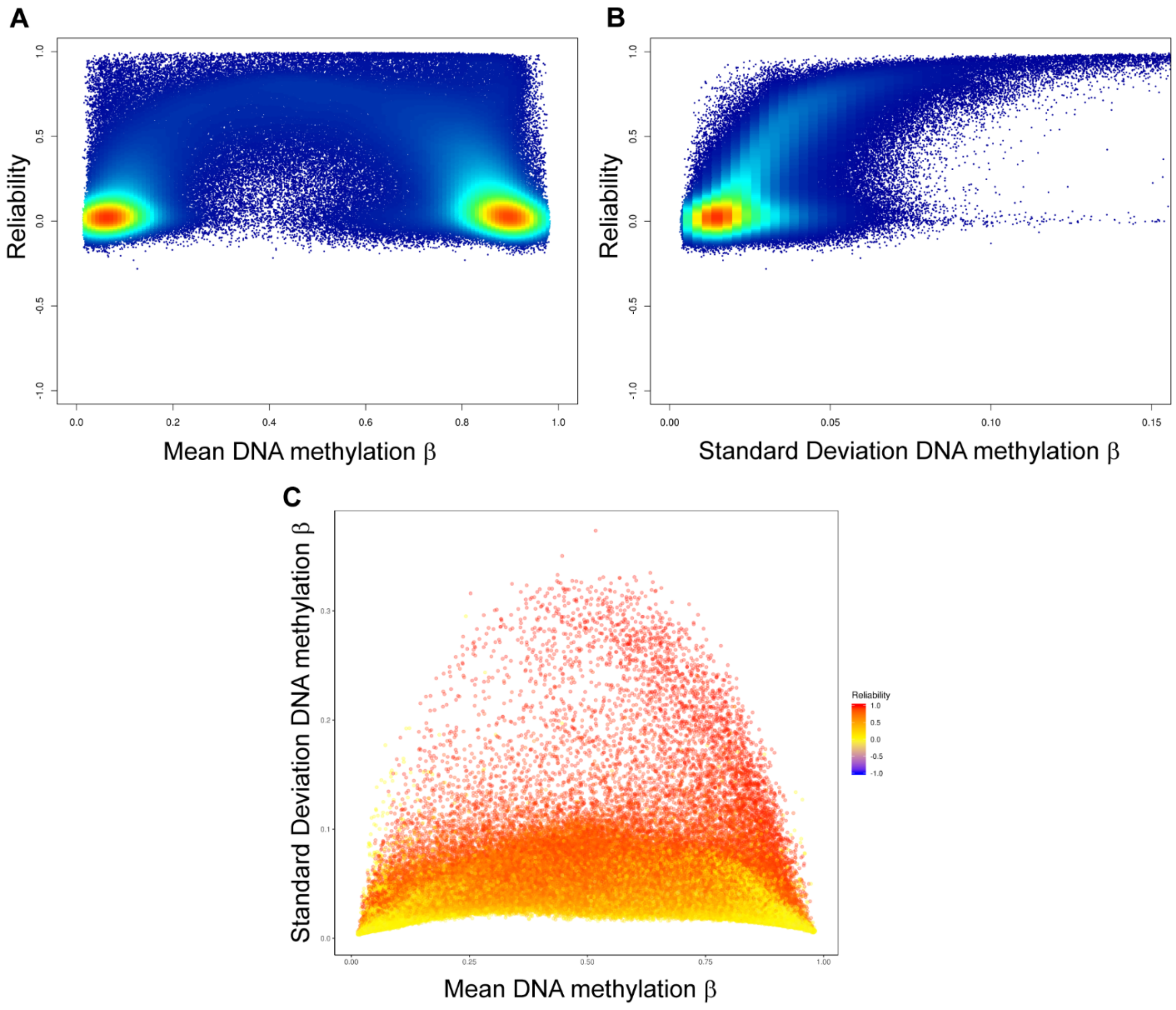

Figure S4: Probe-specific characteristics are related to the distribution of probe reliability. (A) shows a density heatmap of mean DNA methylation level (methylation β, range = 0-1; x-axis) plotted against reliability (Y-axis). This distribution follows an inverted U-shaped curve, where lowest reliabilities tend to be observed where mean β levels are close to either extreme, whereas the highest reliability probes were concentrated in the intermediate range of the distribution. **(B)** shows a density heatmap of the standard deviations of DNA methylation (x-axis) plotted against reliability (Y-axis). Lowest reliabilities tend to be observed where variation in β-levels is the lowest. **(C)** shows means (x-axis) and standard deviations (y-axis) of methylation β-values plotted as a function of reliability (color; red = highest, blue = lowest). Methylation β-level means and SDs are correlated (r=0.15, P<0.01) and show an inverse-U relationship with variability; the most variable probes tend to have mean levels of methylation around the center of the distribution. These variable, intermediately-methylated probes also tend to be most reliable.

1.3 Genomic annotation of probes is related to differential reliability. **Figure S5A** shows that there are regional differences in the distribution of probe reliability (**Data S1**). The transcription start site (TSS) had the highest aggregation of unreliable probes; the intergenic region had the lowest. In addition, CpG islands had a higher aggregation of unreliable probes than CpG shores (**Figure S5B**), a pattern consistent with previous reports[4,5]. This could be due to the fact that sites within CpG islands are more likely to be unmethylated[6] and are therefore more likely to be unreliably measured, or it could be because the proportion of Type I Infinium probes in CpG islands is greater than in CpG shores[7] (vs. Type II; the two probe types differ in the chemistry used to quantify methylation level), and Type I probes are more unreliable than Type II[4,5] (**Figure S5C**).

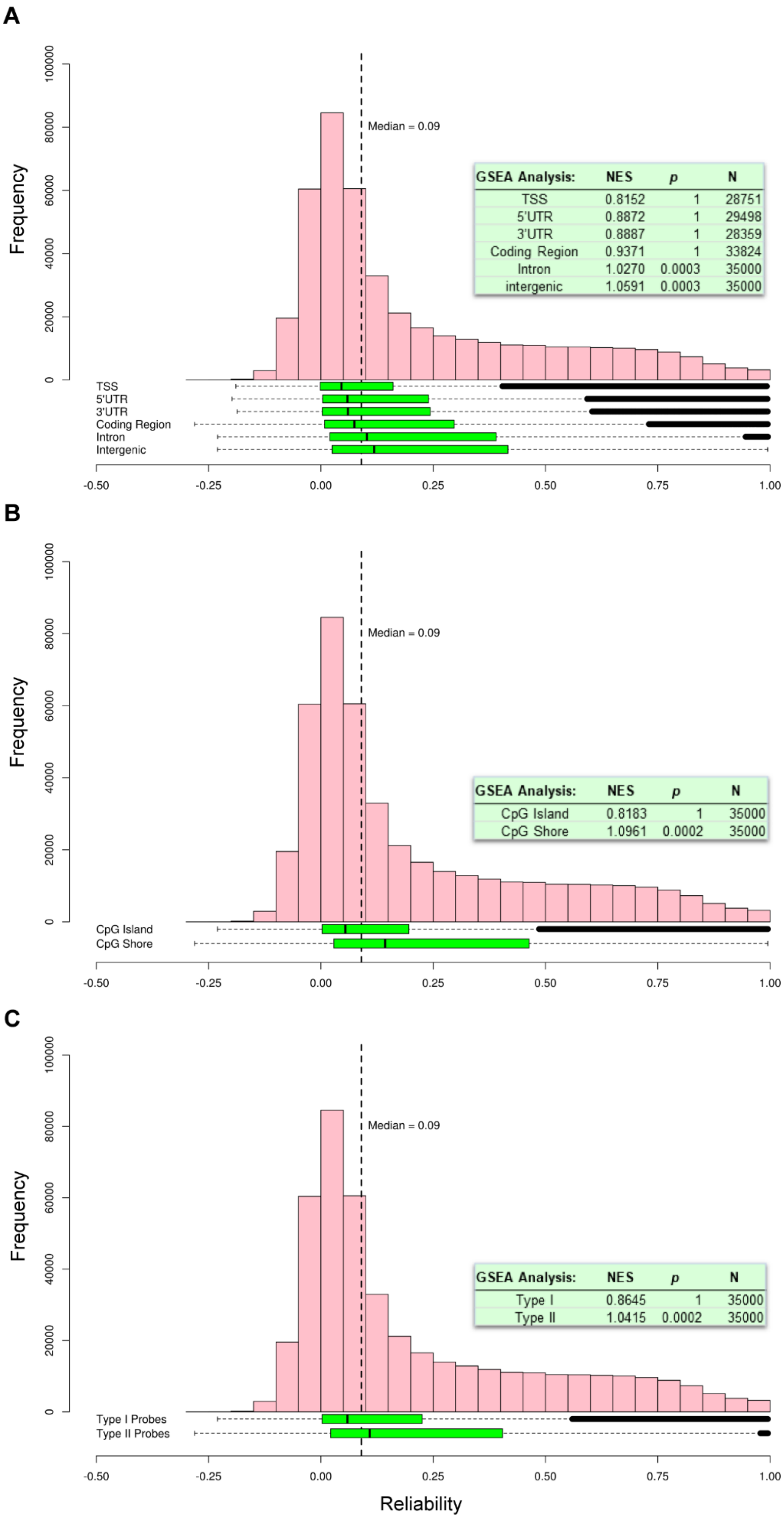
A
Frequency
Median = 0.09
GSEA Analysis: NES p N
TSS 0.8152 1 28751
5'UTR 0.8872 1 29498
3'UTR 0.8887 1 28359
Coding Region 0.9371 1 33824
Intron 1.0270 0.0003 35000
intergenic 1.0591 0.0003 35000
TSS
5'UTR
3'UTR
Coding Region
Intron
Intergenic
B
Frequency
Median = 0.09
GSEA Analysis: NES p N
CpG Island 0.8183 1 35000
CpG Shore 1.0961 0.0002 35000
CpG Island
CpG Shore
C
Frequency
Median = 0.09
GSEA Analysis: NES p N
Type I 0.8645 1 35000
Type II 1.0415 0.0002 35000
Type I Probes
Type II Probes
Reliability

Figure S5: Reliabilities of probes as a function of spatial characteristics. (A) plots the distributions of reliability coefficients as box and whisker plots for probes annotated to one of six genic regions: transcription start site (TSS), 5' untranslated region (5'UTR), 3' untranslated region ('3UTR), coding region, intronic region, and intergenic region. Boxes correspond to Inter-quartile range (IQR), and whiskers extend to 1.5 * IQR. Observations beyond the whiskers (outliers) are represented by individual points. The TSS has the greatest proportion of unreliable probes, the intergenic region the least. **(B)** shows the distribution of reliability coefficients for probes localized to CpG islands or CpG shores. Unreliable probes are more common in CpG islands than CpG shores. Also shown is the distribution of reliability correlations as a function of Infinium probe type; older Type I probes are less reliable than Type II probes **(C).** This could be due to the fact that sites within CpG islands are more likely to be unmethylated and are therefore more likely to be unreliably measured, or it could be because the proportion of Type I Infinium probes in CpG islands is greater than in CpG shores (vs. Type II; the two probe types differ in the chemistry used to quantify methylation level), and Type I probes are more unreliable than Type II. As a reference, the distribution (pink bars) and median (vertical dashed line) of all ~440,000 probe reliabilities in the E-Risk reliability dataset are shown above the box and whisker plots. The text box shows the results of Gene Set Enrichment Analysis (GSEA) for the each set of features; NES= Normalized Enrichment Score, *p*= p-value, N = number of probes. NESs greater than 1 indicate enrichment for reliable probes.

1.4: Low reliability is not artefactual. Previous methodological studies have drawn attention to three factors that might compromise the quality of methylation BeadChip data: probe invariance[8-10], potential probe hybridization problems[11], and skewness. We tested whether these features are sufficient to capture unreliability. They are not. **Figure S6A** and **S6B** document that probe unreliability exists in probes that are variable, and do not have potential probe hybridization problems. **Figure S6C** demonstrates that probe reliabilities calculated on β-values resemble the reliabilities of M-values, a method for transforming skewed probe distributions[12].

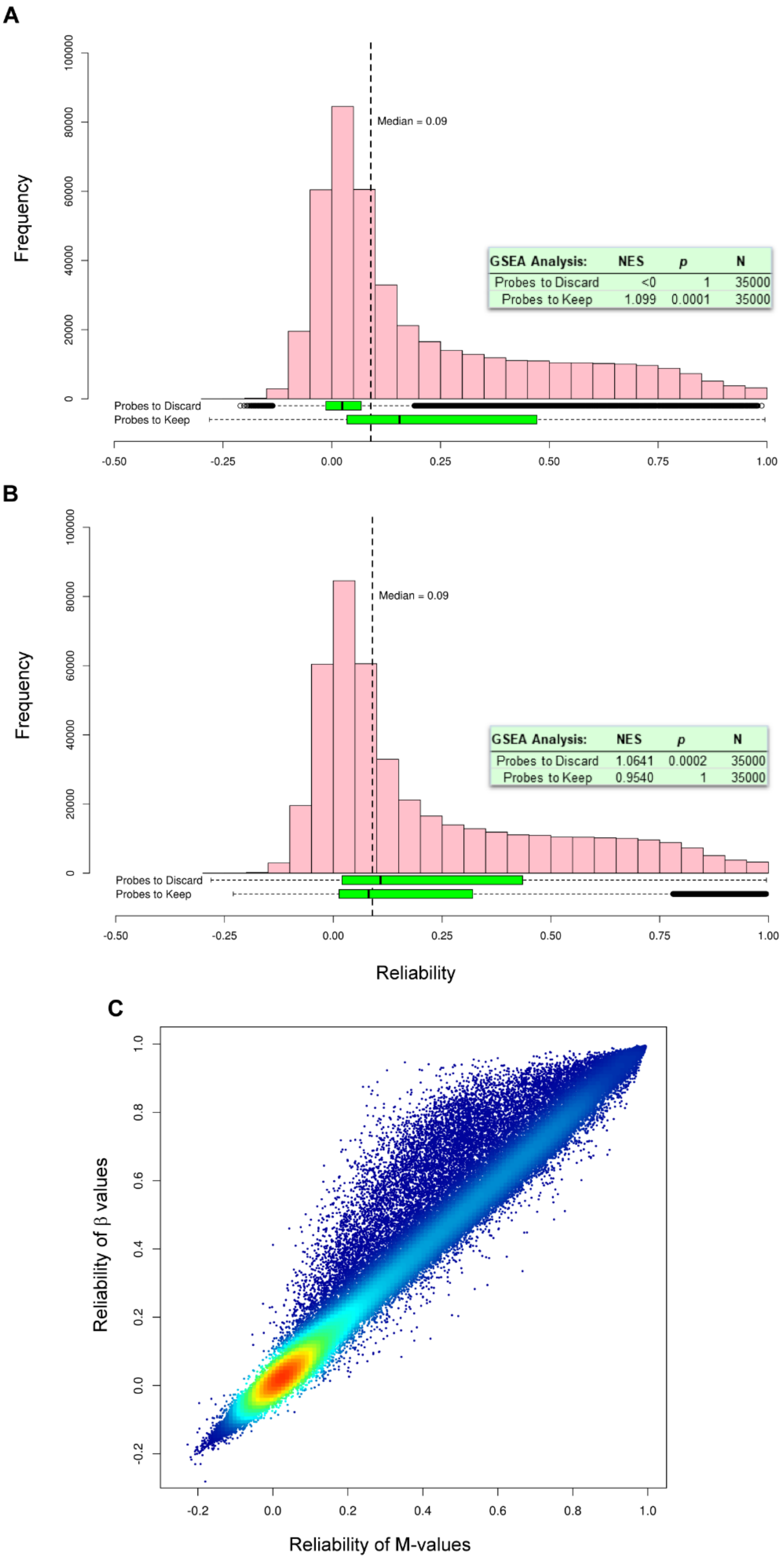
A
Frequency
Median = 0.09
GSEA Analysis: NES p N
Probes to Discard <0 1 35000
Probes to Keep 1.099 0.0001 35000
Probes to Discard
Probes to Keep
B
Frequency
Median = 0.09
GSEA Analysis: NES p N
Probes to Discard 1.0641 0.0002 35000
Probes to Keep 0.9540 1 35000
Probes to Discard
Probes to Keep
Reliability
C
Reliability of β values
Reliability of M-values

Figure S6: The distribution of reliabilities of probes identified as potentially problematic in previous studies. Distributions are depicted as box and whisker plots of the reliability coefficients of the probes identified as variant/invariant by Edgar *et al.* (**A;** probes to discard are invariant probes) or having potential hybridization problems as described by Naaem *et al.* (**B;** probes to discard are probes with hybridization problems). Boxes correspond to Inter-quartile range (IQR), and whiskers extend to 1.5 * IQR. Observations beyond the whiskers (outliers) are represented by individual points. Both variant and non-problematic probe lists ('probes to keep') contain unreliable probes, suggesting these factors alone are not sufficient to index reliability. As a reference, the distribution (pink bars) and median (vertical dashed line) of all ~440,000 probe reliabilities in the E-Risk dataset are shown above the box and whisker plots. The text box shows the results of Gene Set Enrichment Analysis (GSEA) for the suggested set of probes to keep or discard in each situation (NES= Normalized Enrichment Score, *p*= p-value, N = number of probes). NESs greater than 1 indicate enrichment for reliable probes. **(C)** compares the reliability of probes computed using β values against those using M-values. Transforming β values to M-values has little effect on estimates of reliability. These three methods of accounting for unreliable probe data are not fully satisfactory.

In summary, we replicated previous reports of low reliability across probes common to the 450K and EPIC BeadChips, demonstrating that, paradoxically, poor reliability is reproducible. Moreover, factors commonly thought to account for unreliability (such as genomic location, invariance and skewness) do not provide a satisfactory account of its ubiquity.

Section S2: Testing the sensitivity of associations with reliability in light of probe variability

This section relates to Main Text Discussion Section:

'Approaches to improve replicability via reliability assessment.'

We demonstrated that probe reliability is related to various properties of probe measurements (e.g. probe variability, **section S1.2** above). These observations might lead one to ask: are these properties the major drivers of reliability, such that it is unreasonable to assess reliability without their adequate consideration?

We tested this assumption using variability as a case in point. Our reasoning was that If variability is the major driver of reliability, then it follows that exclusion of invariant probes should increase the power to detect associations between reliability and the factors we outline in the main text of the manuscript. We subset our data to only those probes identified as not invariant in blood by Edgar *et al.*[8]. We then repeated our analysis of a) the association between probe reliability and estimates of genetic and environmental influences on DNA methylation, b) the association with mQTL probes, and c) the association with the extent of concordance in DNA methylation levels between blood and brain tissue.

We first tested if the probes identified as invariant by Edgar *et al.*[8] had the same distribution of reliabilities as probes that we independently determine as invariant within our own data. As shown in **Figure S7** (below), the overlap of reliabilities of the probes listed by Edgar *et al.*[8] and probes identified within our data is very high, suggesting that characteristics of individual probes (such as probe variance) are highly reproducible and unlikely to result from experimental-specific artifacts. As such, we went forward to subset our data to only those probes that were not invariant (i.e. 'variant') and repeated our tests of association outlined above.

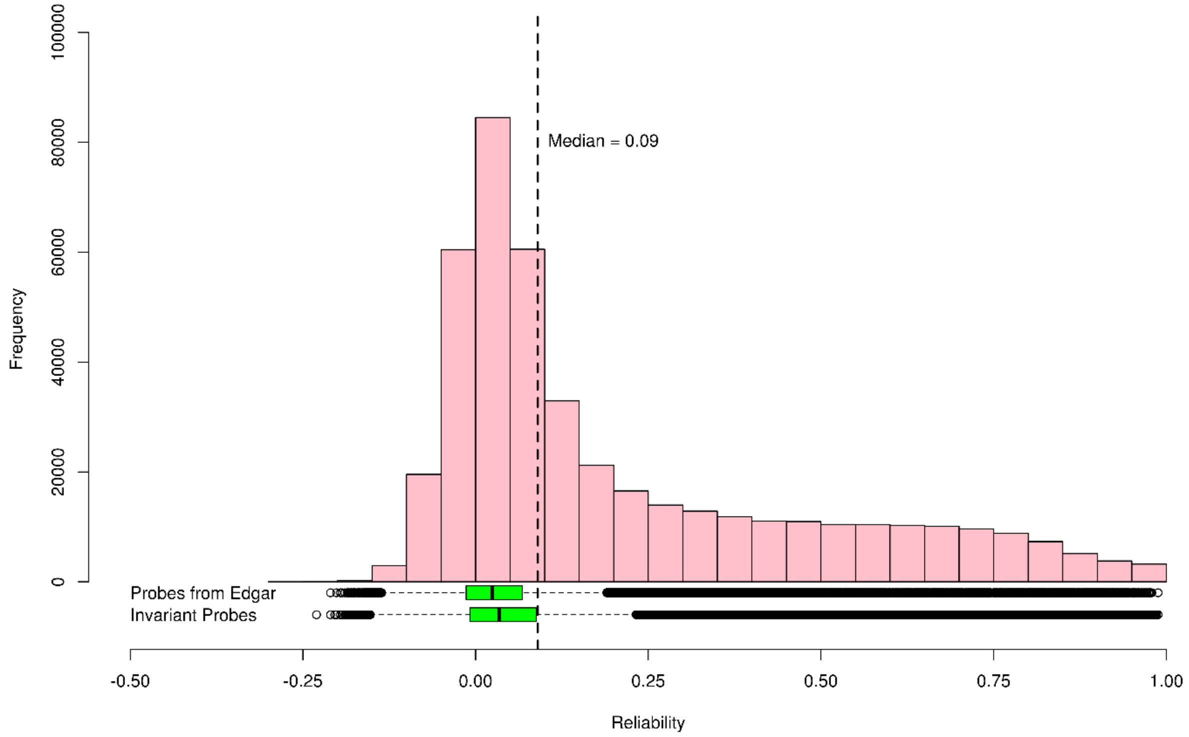

Figure S7. Comparison of reliabilities of invariant probes. Distributions are depicted as box and whisker plots of the reliability coefficients of the probes identified as invariant by Edgar *et al.,* (top box) or identified as invariant based on our own data (bottom box). Boxes correspond to Inter-quartile range (IQR), and whiskers extend to 1.5 * IQR. Observations beyond the whiskers (outliers) are represented by individual points. The distribution of reliability in both sets of invariant probes are similar, suggesting the lists are highly conserved across studies. As a reference, the distribution (pink bars) and median (vertical dashed line) of all ~440,000 probe reliabilities in the E-Risk dataset are shown above the box and whisker plots.

2.1: Associations between probe reliability and estimates of genetic and environmental influences on DNA methylation. In our manuscript, we report that estimates of additive genetic variation were positively correlated with reliability, and estimates of non-shared environmental variation (which also inlcudes measurement error) were negatively associated with reliabilty.

When restricting analysis to just those probes that are variable, we find little attenuation of the association between reliability and these estimates (**Table S1**, below). It is not purely variability driving the associations, since excluding invariant probes does not improve the power to detect associations.

Table S1. Correlations of reliability and ACE parameters

	**All probes (*N* = 430,802)**		**Variant probes only (*N* = 292,127)**	
	r	95% CI	*r*	95% CI
**Additive genetic variation (A)**	0.702	0.701, 0.0704	0.705	0.703, 0.706
**Shared environmental variation (C)**	-0.073	-0.076, -0696	-0.039	-0.042, -0.035
**Non-shared environmental variation (E)**	-0.583	-0.584, -0.5805	-0.657	-0.659, -0.655

<u>2.2: Associations between probe reliability and mQTL probes</u>. In our manuscript, we report that methylation Quantitative Trait Loci (mQTLs)--DNA sequence variants that are associated with differential DNA methylation--are more likely to be associated with reliable probes than unreliable probes.

When restricting our analysis to just those probes that are variable, we find little change in the extent to which the list of mQTL-associated probes is enriched for reliable probes (**Table S2**, below). It is not purely variability driving the ability to detect associations between sequence variants and differential DNA methylation.

Table S2. **GSEA (enrichment) analysis of mQTL- and non mQTL indexing probes**

	**All probes (*N* = 438,593)**		**Variant probes only (*N* = 334,449)**	
	Normalized Enrichment Score	*p* value	Normalized Enrichment Score	*p* value
**mQTL probes**	1.477	0.002	1.525	0.0002
**non-mQTL probes**	0.867	1.00	0.850	1.00

<u>2.3: Associations of probe reliability with the extent of concordance in DNA methylation levels between blood and brain tissue.</u> In our manuscript, we report that probes that show similar levels of DNA methylation in blood and any of four different brain regions ('blood-brain' concordance) are more likely to be reliably measured.

When restricting our analysis to just those probes that are variable, we find little attenuation of the association between reliability and blood-brain concordance (**Table S3**, below). It is not purely variability driving the ability to detect blood-brain concordance.

Table S3. correlations of reliability and concordance of methylation values between blood and each of four brain regions

	**All probes (*N* = 438,593)**		**Variant probes only (*N* = 334,449)**	
**Blood-brain region concordance**	*rho*	95% CI	*rho*	95% CI
**Prefrontal Cortex**	0.348	0.345, 0.351	0.362	0.359, 0.365
**Entorhinal Cortex**	0.315	0.312, 0.317	0.360	0.357, 0.363
**Superior Temporal Gyrus**	0.376	0.373, 0.379	0.390	0.387, 0.393
**Cerebellum**	0.218	0.215, 0.222	0.218	0.215, 0.221

In summary, variability, though highly related to reliability, is not sufficient to account for the challenges posed by unreliable DNA methylation measurement.

Section S3: Additional Experimental Procedures

S3.1: Sample description and data production

Environmental Risk (E-Risk) Longitudinal Twin Study

Sample Description. Participants were members of E-Risk, which tracks the development of a 1994-95 birth cohort of 2,232 British children[13]. Briefly, the E-Risk sample was constructed in 1999-2000, when 1,116 families (93% of those eligible) with same-sex 5-year-old twins participated in home-visit assessments. This sample comprised 56% monozygotic (MZ) and 44% dizygotic (DZ) twin pairs; sex was evenly distributed within zygosity (49% male). The study sample represents the full range of socioeconomic conditions in Great Britain, as reflected in the families' distribution on a neighborhood-level socioeconomic index (ACORN [A Classification of Residential Neighbourhoods], developed by CACI Inc. for commercial use): 25.6% of E-Risk families live in "wealthy achiever" neighborhoods compared to 25.3% nationwide; 5.3% vs. 11.6% live in "urban prosperity" neighborhoods; 29.6% vs. 26.9% in "comfortably off" neighborhoods; 13.4% vs. 13.9% in "moderate means" neighborhoods; and 26.1% vs. 20.7% in "hard-pressed" neighborhoods. E-Risk underrepresents "urban prosperity" neighborhoods because such households are often childless.

Home visits were conducted when participants were aged 5, 7, 10, 12 and most recently, 18 years (93% participation). The Joint South London and Maudsley and the Institute of Psychiatry Research Ethics Committee approved each phase of the study. Parents gave informed written consent and twins gave written assent between 5-12 years and then informed written consent at age 18.

At age 18, 2,066 participants were assessed, each twin by a different interviewer. The average age at the time of assessment was 18.4 years (SD = 0.36); all interviews were conducted after the 18th birthday.

Genome-wide quantification of DNA methylation. Our epigenetic study used DNA from a single tissue: blood. At age 18, whole blood was collected from 82% (N=1700) of the participants in 10mL K_2EDTA tubes. DNA was extracted from the buffy coat using a Flexigene DNA extraction kit (Qiagen, Hilden, Germany) following manufacturer's instructions. Study members who did not provide blood provided buccal swabs, but these were not included in our methylation analysis to avoid tissue-source confounds. Assays were run by the Complex Disease Epigenetics Group at the University of Exeter Medical School, and as described in full in previous publications[9,14]. 450K BeadChip data were available for 1658 study members.

Reliability dataset. For our reliability analysis we selected 350 individuals to assay with the EPIC BeadChip. ~500ng of DNA from each sample was treated with sodium bisulfite using the EZ-96 DNA Methylation kit (Zymo Research, CA, USA). DNA methylation was quantified using the Infinium MethylationEPIC ('EPIC') BeadChip run on an Illumina iScan System (Illumina, CA, USA) by the Complex Disease Epigenetics Group at the University of Exeter Medical School.

Reliability Dataset Processing and Normalization. The EPIC and 450K BeadChip data that comprise the reliability dataset were imported into the minfi Bioconductor package[15,16]. Probes were excluded if they had a detection p-value > 0.05 in at least 10% of the samples in either the EPIC or the 450K BeadChip datasets. Data were processed using the subset-quantile within array normalization

(SWAN) approach to eliminate systematic differences across the arrays. This method was chosen because it is currently one of the very few methods that allows normalization of 450K and EPIC BeadChip data together. Probes were kept for subsequent analysis if they passed the detection p-value threshold in both technologies, were shared between the two array platforms, and did not map to a sex chromosome.

Low reliability might arise through experimental factors not related solely to poor probe performance. We therefore tested two ways in which normalization might affect reliability estimates. First, low reliability could be due to data handling differences between datasets. To test this, we compared reliability coefficients after normalizing the datasets in two ways: (a) where data from 450K and EPIC BeadChips were normalized as separate datasets and (b) where they were normalized together as one dataset. The different normalization strategies had little effect on reliability estimates (**Figure S8A,** r = 1.00, $p < 0.01$), suggesting differential probe reliability was not a product of data-handling practices. The ‘normalized separately’ set is used for all analyses unless otherwise noted.

Second, low reliability could be due to differences in relative ranks of probes induced through use of specific normalization methods. To test this, we re-normalized our data using an alternative method (‘’Quantile”) to that we have employed (“SWAN”), and compared the reliabilities generated using each. Normalization method had little effect on reliability measures (**Figure S8B**, r = 0.98, $p < 0.01$), suggesting our results are not affected by normalization strategy.

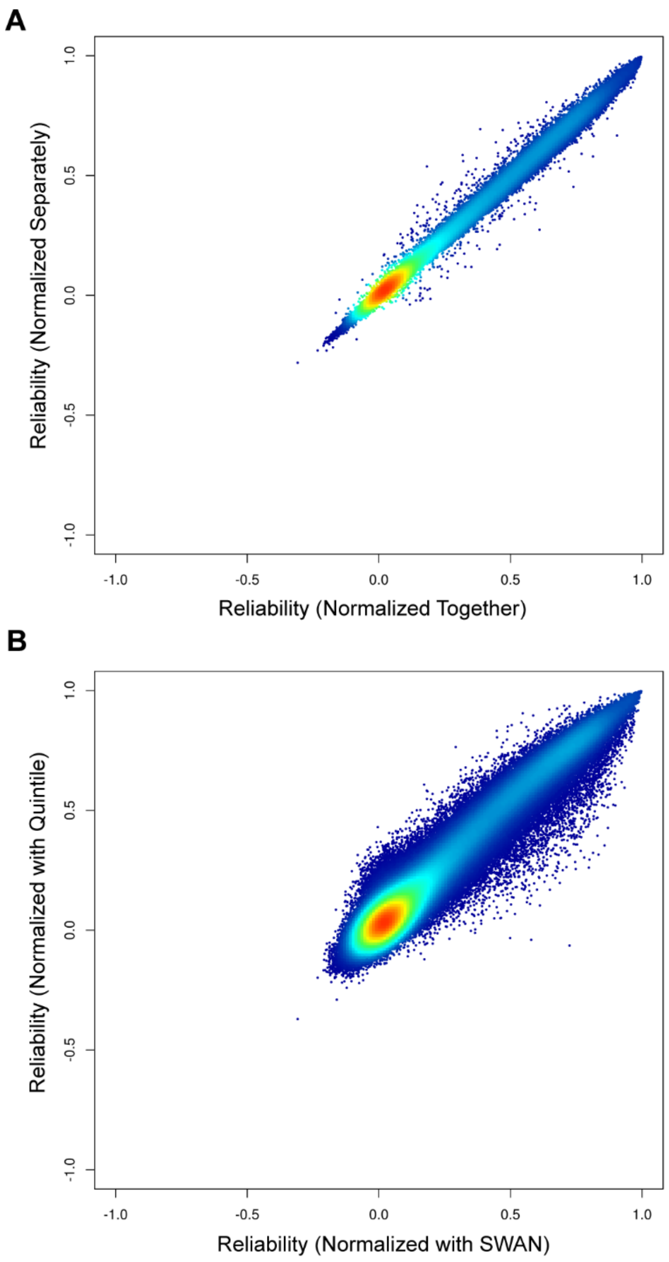

Figure S8: Reliability correlations for probes common to the 450K and EPIC BeadChips. **(A)** compares the reliability correlations generated when data for each BeadChip type were normalized together (x-axis) or normalized separately (y-axis). **(B)** compares the reliability correlations generated using ‘SWAN’, as reported in the main text of the manuscript (x-axis), and those generated using data normalized with ‘Quantile’ (y-axis). In either case, normalization strategy seems to have little effect on the distribution of probe-probe reliability correlations.

Dunedin Longitudinal Study

Sample description. Participants were members of the Dunedin Multidisciplinary Health and Development Study, a longitudinal investigation of health and behavior in a representative birth cohort[17]. Study members (n = 1,037; 91% of eligible births; 52% male) were all individuals born between April 1972 and March 1973 in Dunedin, New Zealand, who were eligible for the longitudinal study based on residence in the province at 3 years of age and who participated in the first follow-up assessment at 3 years of age. The cohort represented the full range of socioeconomic status on NZ's South Island. On adult health, the cohort matches the NZ National Health and Nutrition Survey (e.g., BMI, smoking, GP visits)[17]. The cohort is primarily white (93%); genetic analyses were restricted to non-Maori participants. Assessments were carried out at birth and at ages 3, 5, 7, 9, 11, 13, 15, 18, 21, 26, 32, 38 and 45 years, when 94% of the 997 study members still alive took part. The Otago Ethics Committee approved each phase of the study and informed consent was obtained from all study members.

Genome-wide quantification of DNA methylation using 450K BeadChips. Our epigenetic study used DNA from a single tissue: blood. Whole blood was collected in 10mL K_2EDTA tubes from N = 857 participants at age 38. DNA was extracted from the buffy coat using standard procedures[18,19]. Study members who did not provide blood provided buccal swabs, but these were not included in our methylation analysis to avoid tissue-source confounds.

We assayed 835 blood samples (out of 857); 22 samples were not useable. ~500ng of DNA from each sample was treated with sodium bisulfite using the EZ-96 DNA Methylation kit (Zymo Research, CA, USA). DNA methylation was quantified using the Illumina Infinium HumanMethylation450 BeadChip ("Illumina 450K BeadChip") run on an Illumina iScan System (Illumina, CA, USA) at the Molecular Genomics Core at the Duke Molecular Physiology Institute and are described in full in previous publications[14].

Genome-wide quantification of DNA methylation using EPIC BeadChips. To assay within-array reliability of the EPIC BeadChip, we selected 28 individuals from the Age 45 data collection phase of the Dunedin Study and assayed their DNA twice. DNA was collected from blood and extracted as above. ~500ng of DNA from each sample was treated with sodium bisulfite using the EZ-96 DNA Methylation kit (Zymo Research, CA, USA). DNA methylation was quantified using the Infinium MethylationEPIC ('EPIC') BeadChip run on an Illumina iScan System (Illumina, CA, USA) at the Molecular Genomics Core at the Duke Molecular Physiology Institute. Data were processed, underwent quality control filtering, and normalized as described above for the 350-sample reliability dataset.

Gene Expression. Expression data were generated from whole-blood RNA using the Affymetrix PrimeView Human Gene Chip (Affymetrix, CA, USA). Briefly, these arrays simultaneously interrogate more than 38,000 gene transcripts across the entire genome. Whole-blood RNA samples collected via PaxGene Blood RNA tubes (Qiagen, CA, USA) at age 38 were assayed. Samples were arranged into batches of 60. Array processing was performed by the Duke University Microarray Core Facility using the Affymetrix GeneChip system (Affymetrix). Prior to hybridization, total RNA was assessed for quality with Agilent 2100 Bioanalyzer G2939A (Agilent Technologies, Santa Clara, CA)) and Nanodrop 8000 spectrophotometer (Thermo Scientific/Nanodrop, Wilmington, DE). Samples with RIN ≥ 6 were then subject to globin mRNA depletion using the GLOBINClear –human kit (Ambion, Thermo Fisher Scientific, MA, USA). RNA samples from 843 individuals were assayed. Data quality control and RMA normalization were carried out using the *affy* Bioconductor package[20] in the R statistical programming environment. After QC, expression data were available for 836 individuals.

S3.2: Data analysis

Data analysis was performed in the R statistical programming environment, often using Bioconductor packages. Data handling was performed using the package *dplyr* [21] and descriptives were generated using the package *psych*[22]. Plots were produced in R using the packages *ggplot2*[23] and *ggpubr*[24] where appropriate. Density heatmaps were generated using the *KernSmooth* package[25]. Unless otherwise noted, correlations are reported as two-tailed Pearson product-moment correlation coefficients. Intraclass correlation was calculated using the *irr* package[26].

Probe reliabilities. Probe reliabilities are computed using Intraclass Correlations (ICC), calculated for each autosomal probe present on both the EPIC and 450K BeadChip (*N*=438,593). ICCs are an oft-used metric to assess reliability in test-retest situations[27], and many different models exist depending on the way in which the test-retest data are generated. Here, we calculated ICCs based on a mean-rating (k=2), absolute-agreement, 2-way random-effects model. We chose this model using the guidelines outlined in Koo and Li[27], where mean-rating (k=2) relates to the number of repeated measures (i.e., BeadChips per sample); absolute agreement requires that not only do the values across BeadChips correlate, but that values are in agreement; and 2-way random effects relates to the generalizability of the ICCs to any subsequent similarly characterized rater (where rater = BeadChip probe). To compare whether test-retest model choice had an effect on reliability estimates, we also computed Pearson product-moment correlation coefficients. Pearson correlation coefficients and ICC estimates of reliability were highly similar (r=1.00, P=<1×10^{-4}; **Figure S9**).

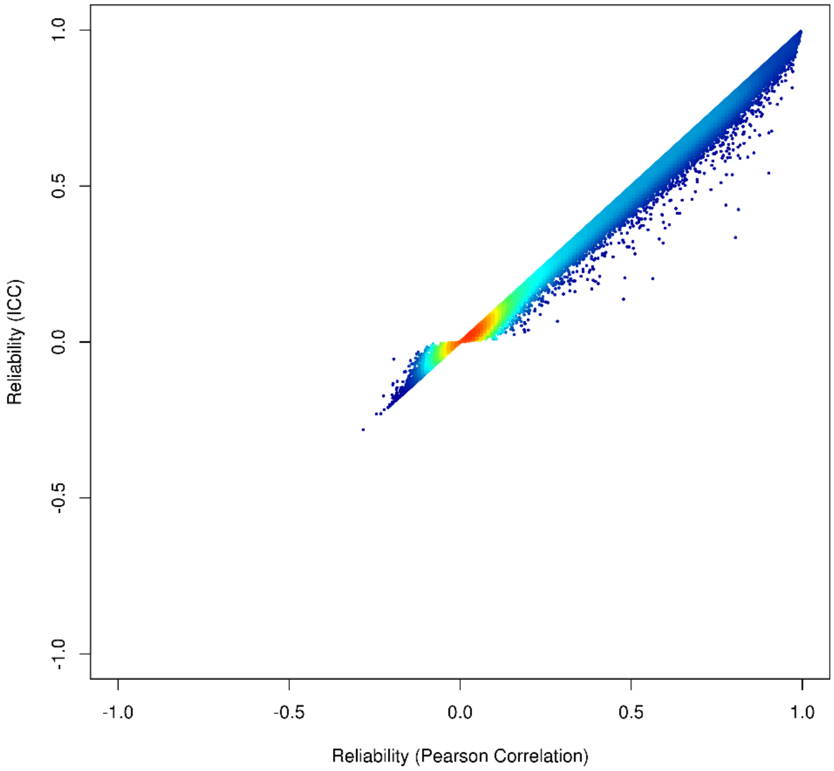

Figure S9: Reliabilities expressed as Pearson correlation coefficients and Intra-Class Coefficients are similar (Refers to Main Text Experimental Procedures section "Probe Reliabilities"). The y-axis plots probe reliabilities as Pearson correlation coefficients and the x-axis plots the Intra-Class Coefficients (ICC. Reliabilities were highly correlated (r = 1.00).

Gene Set Enrichment Analysis. Gene Set Enrichment Analysis (GSEA) was performed using the *fgsea* Bioconductor package[28] with 10,000 permutations. We tested if each probe list was significantly enriched for more highly reliable probes. Due to computing constraints, if a list had more than 35000 probes, it was truncated down to a random sampling of 35000 probes for the analysis.

Structural equation modelling. Biometrical modelling was applied to every probe passing QC on the Illumina 450K array. Specifically, an ACE model was fitted to calculate the proportion of variance in DNA methylation explained by additive genetic (A), shared environmental (C) and unshared or unique environmental (E) factors, the latter which also includes measurement error. The assumptions behind this model are that additive genetic factors are perfectly correlated between MZ twins (i.e. genetic correlation = 1) but are only 50% correlated between DZ twins (i.e. genetic correlation = 0.5) and that shared non-heritable influences are equally similar between MZ and DZ twin pairs. The model was fitted using structural equation modelling implemented with functions from the *OpenMx* R package[29,30].

Identification of Smoking-related DNA methylation probes. We identified 22 studies that reported an epigenome-wide analysis of current vs never smoking using the 450K BeadChip platform[31-37]. For each study, we obtained lists of probe IDs and direction-of-effect for probes that were significantly associated with current smoking (as determined by the study authors; total number of probes=3,724; *N* probes per study=84-2,441). We then determined the extent to which individual probes replicated across the 22 studies by summing the number of times each probe was listed with consistent direction-of-effect (i.e., consistent cross-study increases or decreases in methylation in response to smoking). Descriptions of the studies included are found in **Table S4**.

Table S4. Descriptions of the studies included in analysis of consistency of replication for DNA methylation-smoking associations (Refers to Main text Result item "*Probe reliability impacts association testing*"). Descriptives are derived from the original publications. Information on the 16 studies included in the meta-analysis by Joehanes *et al.,* (2016) is individually listed.

Publication	*Cohort*	*Sample Origin*	*N (% smokers)*	*% male*	*Age; mean (SD), where available*	*N probes significant**	*N probes with available reliability data*	*Additional Notes*
**Zellinger *et al.,* (2013)**	KORA F3 and F4	Whole Blood	1011 (26.0) and 468 (50.4)	60.3 and 49.4	56.96 (46-76)	187	174	sites replicated across F3 and F4
**Besingi *et al.,* (2014)**	NSPHS	Whole Blood	421 (10.2)	53.0	14 - 94	95	84	
**Dogan *et al.,* (2014)**	FACHS	PBMCs	111 (45.0)	0.0	48.1 +/- 7	910	840	African American participants
**Guida *et al.,* (2015)**	EPIC and NOWAC	Buffy coat	745 (23.8)	0.0	53.1 (7.4); 55.4 (4.3)	461	431	
**Dogan *et al.,* (2017)**	FHS	Buffy coat	1597 (7.6)	54.9	62.0 - 67.7 (6.5- 8.6)	525	482	current vs non-smoker
**Wilson *et al.,* (2017)**	KORA S4/F4	whole blood	1344 (20.38)	58.1	50.8 (7.8) - 55.1 (9.0)	590	557	
**Joehanes *et al.,* (2016); meta-analysis comprising 16 cohorts (listed individually); each cohort treated as an individual study for current analysis**						2,623**	2,441	
	ARIC	Buffy coat	2848 (25.3)	36.4	56.2 (5.8)			African American participants
	GTP	Whole Blood	286 (32.9)	29.0	43.4 (11.7)			African American participants
	CHS AA	Whole Blood	192 (15.6)	34.9	70.4 (4.9)			African American participants
	GENOA	Buffy coat	420 (18.3)	28.8	58.7 (7.9)			African American participants
	FHS	Whole Blood	2648 (10.3)	45.7	62.5 (7.8)			European American participants
	KORA F4	Whole Blood	1797 (14.6)	48.7	57.0 (7.0)			European American participants
	GOLDN	CD4+	992 (7.4)	47.8	44 (13)			European American participants
	LBC 1921	Whole Blood	445 (7.0)	39.6	79.2 (0.5)			European American participants

Publication	***Cohort***	***Sample Origin***	***N (% smokers)***	***% male***	***Age; mean (SD), where available***	***N probes significant****	***N probes with available reliability data***	***Additional Notes***
	LBC 1936	Whole Blood	920 (11.2)	50.5	69.5 (0.7)			European American participants
	NAS	Whole Blood	644 (4.0)	100.0	68.2 (6.1)			European American participants
	Rotterdam	Whole Blood	686 (24.6)	43.6	58.0 (6.8)			European American participants
	Inchianti	Whole Blood	508 (9.8)	45.1	58.9 / 16.8			European American participants
	CHS EA	Whole Blood	184 (12.5)	44.0	74.1 (4.2)			European American participants
	EPIC-Norfolk	Buffy coat	1183 (16.1)	49.6	58.3 (8.4)			European American participants
	MESA	CD14+	1256 (9.1)	48.6	65 (8)			European American, African American and Hispanic participants
	EPIC	Buffy coat	898 (21.8)	0.0	48.9 (8.8)			European American participants

** as identified by Study Authors*
***significant at α = $1x10^{-7}$ level*

Correlation of methylation with gene expression. Each probe in the Dunedin 450K methylation dataset was correlated with each probeset from the Dunedin PrimeView gene expression dataset using Spearman's rank correlation approach. To control for technical variation in the gene expression data, we regressed out the following microarray-based quality metrics described by Peters *et al.*[38]: mean of positive match probesets, mean of positive control probesets, standard deviation of positive control probesets, mean of negative control probesets, standard deviation of negative control probesets, mean of all probesets, standard deviation of all probesets, and relative log expression mean of all probesets, along with sex, array batch and RIN. To control for technical variation in the methylation data, we regressed out the first 32 principal components calculated from the control probes on the arrays. For both datasets, we controlled for cell type composition by regressing out white cell-type counts measured using flow cytometry (Sysmex Corporation, Japan) in whole blood samples taken concurrently with the DNA and RNA samples. Methylation probes that overlapped the transcription start site of at least one isoform of each gene represented by a gene expression probeset were kept for subsequent analysis. For each methylation probe, the gene expression probeset with the highest Spearman correlation coefficient was retained as the representative probeset for the expression level of that gene. Thus, each methylation probe is reported as correlated with a single gene expression probeset. A methylation-expression correlation coefficient was considered significant if it had a *p*-value <= $1x10^{-7}$.

Determination of the number of replicates needed to identify reliable probes. The 350 samples used for the reliability analysis were randomly ordered. Reliability was calculated on growing subsets of the data that were needed to consistently identify probes that had a reliability >= 0.75 in the full set of 350 samples (**Figure S10**).

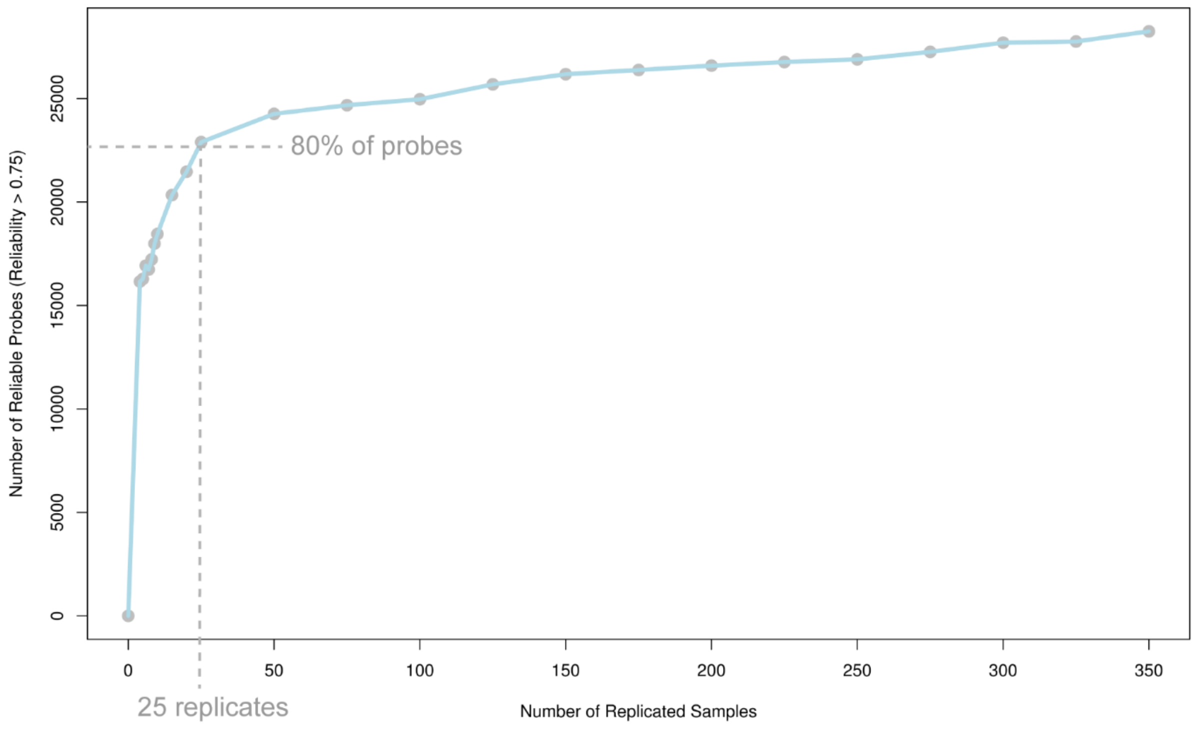

Figure S10: Simulation of the number of replicate BeadChips needed to identify reliable probes. Simulations suggests that 25 replicates would be sufficient to capture 80% of the probes with reliability > 0.75 observed in the dataset of 350.

References

1. Cicchetti, D.V., and Sparrow, S.A. (1981). Developing criteria for establishing interrater reliability of specific items: applications to assessment of adaptive behavior. Am J Ment Defic 86, 127-137.

2. Logue, M.W., Smith, A.K., Wolf, E.J., Maniates, H., Stone, A., Schichman, S.A., McGlinchey, R.E., Milberg, W., and Miller, M.W. (2017). The correlation of methylation levels measured using Illumina 450K and EPIC BeadChips in blood samples. Epigenomics 9, 1363-1371.

3. Dugue, P.A., English, D.R., MacInnis, R.J., Jung, C.H., Bassett, J.K., FitzGerald, L.M., Wong, E.M., Joo, J.E., Hopper, J.L., Southey, M.C.*, et al.* (2016). Reliability of DNA methylation measures from dried blood spots and mononuclear cells using the HumanMethylation450k BeadArray. Sci Rep 6, 30317.

4. Bose, M., Wu, C., Pankow, J.S., Demerath, E.W., Bressler, J., Fornage, M., Grove, M.L., Mosley, T.H., Hicks, C., North, K.*, et al.* (2014). Evaluation of microarray-based DNA methylation measurement using technical replicates: the Atherosclerosis Risk In Communities (ARIC) Study. BMC Bioinformatics 15, 312.

5. Solomon, O., MacIsaac, J., Quach, H., Tindula, G., Kobor, M.S., Huen, K., Meaney, M.J., Eskenazi, B., Barcellos, L.F., and Holland, N. (2018). Comparison of DNA methylation measured by Illumina 450K and EPIC BeadChips in blood of newborns and 14-year-old children. Epigenetics 13, 655-664.

6. Bibikova, M., Le, J., Barnes, B., Saedinia-Melnyk, S., Zhou, L., Shen, R., and Gunderson, K.L. (2009). Genome-wide DNA methylation profiling using Infinium(R) assay. Epigenomics 1, 177-200.

7. Dedeurwaerder, S., Defrance, M., Calonne, E., Denis, H., Sotiriou, C., and Fuks, F. (2011). Evaluation of the Infinium Methylation 450K technology. Epigenomics 3, 771-784.

8. Edgar, R.D., Jones, M.J., Robinson, W.P., and Kobor, M.S. (2017). An empirically driven data reduction method on the human 450K methylation array to remove tissue specific non-variable CpGs. Clin Epigenetics 9, 11.

9. Hannon, E., Knox, O., Sugden, K., Burrage, J., Wong, C.C.Y., Belsky, D.W., Corcoran, D.L., Arseneault, L., Moffitt, T.E., Caspi, A.*, et al.* (2018). Characterizing genetic and environmental influences on variable DNA methylation using monozygotic and dizygotic twins. PLoS Genet 14, e1007544.

10. van Dongen, J., Ehli, E.A., Slieker, R.C., Bartels, M., Weber, Z.M., Davies, G.E., Slagboom, P.E., Heijmans, B.T., and Boomsma, D.I. (2014). Epigenetic variation in monozygotic twins: a genome-wide analysis of DNA methylation in buccal cells. Genes (Basel) 5, 347-365.

11. Naeem, H., Wong, N.C., Chatterton, Z., Hong, M.K., Pedersen, J.S., Corcoran, N.M., Hovens, C.M., and Macintyre, G. (2014). Reducing the risk of false discovery enabling identification of biologically significant genome-wide methylation status using the HumanMethylation450 array. BMC Genomics 15, 51.

12. Du, P., Zhang, X., Huang, C.C., Jafari, N., Kibbe, W.A., Hou, L., and Lin, S.M. (2010). Comparison of Beta-value and M-value methods for quantifying methylation levels by microarray analysis. BMC Bioinformatics 11, 587.

13. Moffitt, T.E., and E-Risk Study Team (2002). Teen-aged mothers in contemporary Britain. J Child Psychol Psychiatry 43, 727-742.

14. Marzi, S.J., Sugden, K., Arseneault, L., Belsky, D.W., Burrage, J., Corcoran, D.L., Danese, A., Fisher, H.L., Hannon, E., Moffitt, T.E., *et al.* (2018). Analysis of DNA Methylation in Young People: Limited Evidence for an Association Between Victimization Stress and Epigenetic Variation in Blood. Am J Psychiatry 175, 517-529.

15. Aryee, M.J., Jaffe, A.E., Corrada-Bravo, H., Ladd-Acosta, C., Feinberg, A.P., Hansen, K.D., and Irizarry, R.A. (2014). Minfi: a flexible and comprehensive Bioconductor package for the analysis of Infinium DNA methylation microarrays. Bioinformatics 30, 1363-1369.

16. Huber, W., Carey, V.J., Gentleman, R., Anders, S., Carlson, M., Carvalho, B.S., Bravo, H.C., Davis, S., Gatto, L., Girke, T., *et al.* (2015). Orchestrating high-throughput genomic analysis with Bioconductor. Nat Methods 12, 115-121.

17. Poulton, R., Moffitt, T.E., and Silva, P.A. (2015). The Dunedin Multidisciplinary Health and Development Study: overview of the first 40 years, with an eye to the future. Soc Psychiatry Psychiatr Epidemiol 50, 679-693.

18. Bowtell, D.D. (1987). Rapid isolation of eukaryotic DNA. Anal Biochem 162, 463-465.

19. Jeanpierre, M. (1987). A rapid method for the purification of DNA from blood. Nucleic Acids Res 15, 9611.

20. Gautier, L., Cope, L., Bolstad, B.M., and Irizarry, R.A. (2004). affy--analysis of Affymetrix GeneChip data at the probe level. Bioinformatics 20, 307-315.

21. Wickham, H., François, R., Henry, L., and Müller, K. (2018). dplyr: A Grammar of Data Manipulation. R package version 0.7.4. .

22. Revelle, W. (2018). psych: Procedures for Personality and Psychological Research. R package version 1.7.8.

23. Wickham, H. (2009). ggplot2 (New York, New York, USA: Springer-Verlag New York).

24. Kassambara, A. (2018). ggpubr: 'ggplot2' Based Publication Ready Plots. R package version 0.1.6.9999

25. Wand, M. (2015). KernSmooth: Functions for Kernel Smoothing Supporting Wand & Jones (1995). R package version 2.23-15.

26. Gamer, M., Lemon, J., Fellows, I., and Singh, P. (2019). irr: Various Coefficients of Interrater Reliability and Agreement. R package version 0.84.1.

27. Koo, T.K., and Li, M.Y. (2016). A Guideline of Selecting and Reporting Intraclass Correlation Coefficients for Reliability Research. J Chiropr Med 15, 155-163.

28. Sergushichev, A.A. (2016). An algorithm for fast preranked gene set enrichment analysis using cumulative statistic calculation (bioRxiv).

29. Boker, S., Neale, M., Maes, H., Wilde, M., Spiegel, M., Brick, T., Spies, J., Estabrook, R., Kenny, S., Bates, T., *et al.* (2011). OpenMx: An Open Source Extended Structural Equation Modeling Framework. Psychometrika 76, 306-317.

30. Neale, M.C., Hunter, M.D., Pritikin, J.N., Zahery, M., Brick, T.R., Kirkpatrick, R.M., Estabrook, R., Bates, T.C., Maes, H.H., and Boker, S.M. (2016). OpenMx 2.0: Extended Structural Equation and Statistical Modeling. Psychometrika 81, 535-549.

31. Dogan, M.V., Beach, S.R.H., and Philibert, R.A. (2017). Genetically contextual effects of smoking on genome wide DNA methylation. Am J Med Genet B Neuropsychiatr Genet 174, 595-607.

32. Besingi, W., and Johansson, A. (2014). Smoke-related DNA methylation changes in the etiology of human disease. Hum Mol Genet 23, 2290-2297.

33. Guida, F., Sandanger, T.M., Castagne, R., Campanella, G., Polidoro, S., Palli, D., Krogh, V., Tumino, R., Sacerdote, C., Panico, S., *et al.* (2015). Dynamics of smoking-induced genome-wide methylation changes with time since smoking cessation. Hum Mol Genet 24, 2349-2359.

34. Joehanes, R., Just, A.C., Marioni, R.E., Pilling, L.C., Reynolds, L.M., Mandaviya, P.R., Guan, W., Xu, T., Elks, C.E., Aslibekyan, S., *et al.* (2016). Epigenetic Signatures of Cigarette Smoking. Circ Cardiovasc Genet 9, 436-447.

35. Wilson, R., Wahl, S., Pfeiffer, L., Ward-Caviness, C.K., Kunze, S., Kretschmer, A., Reischl, E., Peters, A., Gieger, C., and Waldenberger, M. (2017). The dynamics of smoking-related disturbed methylation: a two time-point study of methylation change in smokers, non-smokers and former smokers. BMC Genomics 18, 805.

36. Dogan, M.V., Shields, B., Cutrona, C., Gao, L., Gibbons, F.X., Simons, R., Monick, M., Brody, G.H., Tan, K., Beach, S.R., *et al.* (2014). The effect of smoking on DNA methylation of peripheral blood mononuclear cells from African American women. BMC Genomics 15, 151.

37. Zeilinger, S., Kuhnel, B., Klopp, N., Baurecht, H., Kleinschmidt, A., Gieger, C., Weidinger, S., Lattka, E., Adamski, J., Peters, A., *et al.* (2013). Tobacco smoking leads to extensive genome-wide changes in DNA methylation. PLoS One 8, e63812.

38. Peters, M.J., Joehanes, R., Pilling, L.C., Schurmann, C., Conneely, K.N., Powell, J., Reinmaa, E., Sutphin, G.L., Zhernakova, A., Schramm, K., *et al.* (2015). The transcriptional landscape of age in human peripheral blood. Nat Commun 6, 8570.
